# Distributed Statistical Analyses: A Scoping Review and Examples of Operational Frameworks Adapted to Health Analytics

**DOI:** 10.2196/53622

**Published:** 2024-11-14

**Authors:** Félix Camirand Lemyre, Simon Lévesque, Marie-Pier Domingue, Klaus Herrmann, Jean-François Ethier

**Affiliations:** 1 GRIIS, Université de Sherbrooke, 2500, Boul de l'Université, Sherbrooke, QC, J1K 2R1, Canada, 1-819-821-8000 ext 74977; 2 Département de mathématiques, Faculté des sciences, Université de Sherbrooke, Sherbrooke, QC, Canada; 3 Health Data Research Network Canada, Vancouver, BC, Canada; 4 Chaire MEIE Québec - Le numérique au service des systèmes de santé apprenants, Université de Sherbrooke, Sherbrooke, QC, Canada; 5 Département de médecine, Faculté de médecine et des sciences de la santé, Université de Sherbrooke, Sherbrooke, QC, Canada

**Keywords:** distributed algorithms, generalized linear models, horizontally partitioned data, GLMs, learning health systems, distributed analysis, federated analysis, data science, data custodians, algorithms, statistics, synthesis, review methods, searches, scoping

## Abstract

**Background:**

Data from multiple organizations are crucial for advancing learning health systems. However, ethical, legal, and social concerns may restrict the use of standard statistical methods that rely on pooling data. Although distributed algorithms offer alternatives, they may not always be suitable for health frameworks.

**Objective:**

This study aims to support researchers and data custodians in three ways: (1) providing a concise overview of the literature on statistical inference methods for horizontally partitioned data, (2) describing the methods applicable to generalized linear models (GLMs) and assessing their underlying distributional assumptions, and (3) adapting existing methods to make them fully usable in health settings.

**Methods:**

A scoping review methodology was used for the literature mapping, from which methods presenting a methodological framework for GLM analyses with horizontally partitioned data were identified and assessed from the perspective of applicability in health settings. Statistical theory was used to adapt methods and derive the properties of the resulting estimators.

**Results:**

From the review, 41 articles were selected and 6 approaches were extracted to conduct standard GLM-based statistical analysis. However, these approaches assumed evenly and identically distributed data across nodes. Consequently, statistical procedures were derived to accommodate uneven node sample sizes and heterogeneous data distributions across nodes. Workflows and detailed algorithms were developed to highlight information sharing requirements and operational complexity.

**Conclusions:**

This study contributes to the field of health analytics by providing an overview of the methods that can be used with horizontally partitioned data by adapting these methods to the context of heterogeneous health data and clarifying the workflows and quantities exchanged by the methods discussed. Further analysis of the confidentiality preserved by these methods is needed to fully understand the risk associated with the sharing of summary statistics.

## Introduction

### Health Analytics at Scale

Learning health systems (LHSs) are coming of age and are being deployed to address important health challenges at different scales. The framework starts by leveraging health data created across various activities. It obviously includes data points from clinics and hospitals, but the perimeter of data required to meaningfully and optimally address important problems is much wider and includes research cohorts, biobanks, quantified self-data, environmental exposures, and social service delivery.

While some questions might be addressed at the scale of an individual organization, LHSs focus on system interactions and often require the analysis of processes and outcomes from various organizations. For example, to fully understand a cancer care trajectory, multiple data sources from multiple organizations will need to be examined to cover all relevant aspects (both within the traditional health system and in the community). This often implies organizations that are at least regional or of a wider scope (provinces, states, and countries), such as in the context of the Health Data Research Network Canada or Health Data Research United Kingdom. Similarly, comparing various approaches is often a fruitful way to identify the best approaches and understand what works, why, and how to scale the promising projects. It can also be a way to amass a critical number of observations in the context of rarer diseases. Nevertheless, working with data from multiple sources, from multiple organizations, and located in multiple jurisdictions poses significant challenges.

Traditionally, the analytical methods used by researchers in health-related and other domains have relied on data pooling (sometimes referred to as data centralization)—all required data are physically copied to a single location where analysis can take place. However, when working with data from multiple jurisdictions (even when part of the same country, such as the Canadian provinces and territories), data pooling is often very difficult, if not impossible, for ethical, legal, and social acceptability reasons.

Therefore, there is a pressing need to offer analytical methods allowing for the analysis of such data without the need to physically copy the data in a central location.

The primary intent of this study was to lay the foundations for future practical assessments of the feasibility of conducting distributed statistical analyses in health-related contexts. We achieved this by reviewing the literature on existing methods, evaluating which ones could be applied to a class of widely used regression models, and precisely identifying their operational and information sharing requirements in a notational framework that allows for straight comparisons. This unified framework enables the description of methods’ operational workflows, quantities exchanged, and algorithmic implementation and operates under assumptions commonly satisfied in health analytics.

This paper is structured as follows. We begin with a formal description of the distributed analytical framework considered in this study, followed by a discussion on the challenges associated with its implementation in health analytics. Next, we state our specific objectives and outline the methodology used to achieve them, including a scoping review and our approach to establishing the common notational framework for method description and comparison. After presenting the results pertaining to each objective, we discuss our findings and remaining challenges regarding distributed statistical analyses for health analytics.

### Distributed Analysis

#### Overview

This study was concerned with frameworks in which the data needed for a statistical analysis consisted of the data about *n* individuals (referred to as the *analytical data set*), which are not all stored in a single source but are partitioned among *K* locations that will be called *nodes* hereafter. Therefore, the mereological sum of all the data held at each node forms the analytical data set. Data can be partitioned horizontally or vertically (or in a mixed way).

A horizontal partition implies that all data pertaining to a given individual can be found in a single node. If we assume that patients receive care only in 1 province, Canadian provincial health administrative data sets hosted by organizations such as Population Data BC, the Institute for Clinical Evaluative Sciences in Ontario, or the Manitoba Centre for Health Policy in Manitoba can be part of a horizontal partition. A clinical trial in which each recruiting site captures all data for a given participant is another example.

A vertical partition occurs when all data of a certain type are available in a single node for a group of individuals. A classic example is a hospital with its various information systems. All pathology results can be found in the pathology system, all billing information can be extracted from the finance system, and all x-rays are accessible in the picture archiving and communication system. However, to obtain the full picture of the care received by a patient, multiple systems need to be interrogated. Similarly, in the research setting, health administrative data may be in a provincial data center, and genomics data could be held in a research institute.

A mixed partition occurs when both principles partly apply—some individuals may have their data spread out across nodes, and different individuals may be present in different nodes.

#### Assumptions

The difficulties in conducting analyses on a large scale mentioned previously are often associated with horizontally partitioned data, and this work focused on this type of partition. Therefore, the methods presented in this paper might not be directly applicable to vertically partitioned data.

One group of approaches often labeled as *distributed analysis* involves calculations at each participating node and exchanges of the resulting aggregated statistics with a *coordinating center* (CC), which can itself also perform additional calculations based on the received aggregated statistics. The CC can be an organization not responsible for a data node or a data node taking on the additional role of CC for a given analysis.

It is important to note that, whether in the more traditional way of data pooling or using distributed approaches (in which the data are not copied centrally), data sources will be different on multiple levels. They will represent information using data models with significant variability in terms of structure and technology but also in terms of semantics. This situation also leads to heterogeneous data in which the presence of predictors and outcomes is likely to be different in different nodes. Different approaches (eg, data mediation or extract, transform, and load) have been developed to address these issues, and this work assumed that one of them was applied so that the data nodes mentioned hereafter are assumed to share the same structure, the same technological syntax, and the same semantics, as well as no missing data.

#### Horizontally Partitioned Statistical Analytics

In what follows, the field that pertains to the statistical analysis of horizontally partitioned and semantically homogeneous data that cannot be consolidated into a central location will be called *horizontally partitioned statistical analytics* (HPSA).

Methodological contributions to this field have arisen from several streams of literature. Meta-analysis and meta-regression methods [1] can be viewed as part of HPSA (eg, by considering that each node-specific data set belongs to a different pseudostudy). However, their scope is narrower compared to that of HPSA because they typically assume that only established study-level estimates are available as data. Conversely, HPSA allows for the sharing of additional summary statistics between the nodes and the CC, such as gradients and Hessians, to ensure the best possible performance at the global level. As meta-analysis does not leverage any supplementary information that could be obtained from studies with access to patient-level data, it can be susceptible to biased estimation, especially in settings with rare outcomes or in the presence of data nodes with limited sample sizes [2]. As meta-analysis and meta-regression methods have been extensively covered in the literature, approaches specifically designed for the analysis of already established study-level estimates will not be discussed hereafter.

An important research community that has generated a significant amount of analytical contributions is concerned with the massive data setting. There, a data set often cannot be processed by a single server and, therefore, is split across multiple machines, which are then considered as nodes able to perform computations and send aggregated results to a CC tasked with fitting a global model from them. The methodological avenues proposed in this setting share similarities with the ones designed for the multi–research facility setting involved in LHSs but also have important differences. For example, in a massive data setting, the experimenter has control over the distribution of individuals across nodes, which is typically not the case in multi–research facility studies. Thus, while these approaches share mechanistic similarities and have been suggested as options to consider in the health domain, some hypotheses may not hold. In regression settings, it is often reasonable to assume that the regression link between the response and covariate predictors is the same across nodes. However, assuming that the sampling distribution of covariates involved is equal across nodes is unrealistic in the health domain, particularly due to the presence of data centers that may systematically involve different types of patients. For example, certain clinics may predominantly serve older individuals. While this may not affect the estimation of parameter values, it can have implications for computing CIs to ensure the validity of inferences.

So far, 2 reviews discussing methods applicable to horizontally partitioned data have been published [3,4]. However, their focus is on the massive data setting, which works almost invariably under the assumption of even sampling distribution of covariates and equal sample sizes across nodes, and statistical inference tasks beyond parameter estimation are barely covered. This makes them less helpful for health analytics purposes as most studies involving data analyses rely on CIs or hypothesis testing in settings in which predictors’ distribution and sample sizes vary across nodes.

### Contemporary Challenges in HPSA

#### Overview

The problem is 3-fold. First, there is a need to raise awareness regarding the existence of HPSA approaches among researchers aiming at undertaking statistical analyses from horizontally partitioned data, especially in health analytics. The reflex is often to request data pooling because it is perceived as the sole option. This has been the tendency of requests made by researchers to the Health Data Research Network Canada. Practitioners are usually concerned with finding the most appropriate statistical model that will take into account as many of the features of their specific context of application as possible. Consequently, a clear and unifying mapping of the state of the HPSA field is needed for them to be informed of the scope of existing methods available for their analyses to see whether alternatives to pooling exist.

Second, as underlined previously, methodological contributions came from research fields whose working assumptions can be fundamentally different from the ones researchers would be willing to make in health analytics. To ensure proper use of statistical inference techniques, it is necessary that the underlying assumptions of existing methods be adequately identified and understood. If necessary, these methods should be adapted to suit the specific requirements of health applications, thereby ensuring accurate and reliable results.

Third, data custodians have to be properly informed on data sharing requirements entailed by the use of a specific HPSA method applicable to a given research setting. While HPSA avoids the complexities of pooling data, there are still flows of information that have to be acceptable to data stewards. However, even in basic statistical scenarios, available methods are often presented in a way that makes them challenging to compare in terms of information sharing requirements and operational complexity. Therefore, there is a need for clearer and more accessible presentations of these methods to facilitate decision-making regarding data sharing and operational implementation.

Although it would be ideal to offer managers a comprehensive operational workflow for each identified method to evaluate the information shared and execution complexity, with their accompanying underlying modeling assumptions, the abundance and diversity of available approaches make it unfeasible to accomplish this in a single paper. In fact, methods often differ in terms of their targeted application beyond their distributed aspect. For example, differences may exist in the studied model (eg, linear, logistic or Cox regression, and additive models), the dimensionality and sparsity of the predictor variable space, the use of regularization or shrinkage, the presence of missingness, confounders, imbalances, and heterogeneity.

#### Objectives

The objectives of this study were as follows:

1. To identify and map, from the literature, methodological approaches that make it possible to perform CI estimation and hypothesis testing from a horizontally partitioned data set
2. Among the approaches identified, to describe the ones that allow for the conduct of generalized linear model (GLM) analyses and identify their distributional assumptions
3. On the basis of the approaches identified for GLM-based inferences, to present methods adapted to the setting of uneven sampling distributions across nodes and compare them in terms of information sharing requirements and operational complexity

A scoping review methodology was chosen to achieve objective 1 of mapping the state of the field of HPSA that pertains to inference procedures. For our second objective (objective 2), we identified from the articles selected from the literature search the ones that presented a methodological framework for conducting statistical inference procedures from a GLM with horizontally partitioned data. We then used these frameworks to derive and describe GLM estimators that are applicable to horizontally partitioned data sets. For each identified method, we analyzed and reported its communication workflow and the distributional assumptions. For our third objective (objective 3), we first used statistical theory to adapt the identified procedures to the unequal sample size and uneven covariate distribution setting. Algorithms and mathematical expressions for the quantities involved are reported. For conciseness, we present mathematical formulas for estimation procedures of CIs only. Expressions involved for hypothesis testing are similar and can be deduced following the close connection between CIs and hypothesis tests in GLMs (eg, see Agresti [5]).

The mathematical description of the GLM setting considered for this analysis is described in the following section along with the mathematical notations to be used.

### Mathematical Foundations and Notation

#### Notation

In the following, lowercase letters in bold will represent vector-valued quantities, whereas uppercase letters in bold will denote matrices. The *j*th element of any vector ***a*** ∈ ℝ*^p^* will be denoted as [***a***]*_j_*. Similarly, the entry at position (*j*, *l*) of any matrix ***A***∈ ℝ*^p×p^*will be denoted as [***A***]*_jl_*. If *g* is a real-valued and invertible function, we will use *g*^(–1)^ to represent its inverse. In addition, if *f*_**θ**_ is a real-valued function that depends on a parameter vector ***θ*** and is twice continuously differentiable, ∇_**θ**_*f*_**θ**_ and ∇^2^_**θ**_*f*_**θ**_ will, respectively, indicate the gradient and Hessian matrix of *f*_**θ**_ with respect to ***θ***.

#### Model Mathematical Assumptions

A mathematical depiction of the horizontally partitioned data framework studied in this paper is as follows. There are *n* individuals horizontally partitioned across *K* data storage nodes. Each node’s data set is denoted by $D^{\left(k\right)}=\{z_{i}^{\left(k\right)}={\left(x_{1i}^{\left(k\right)},\ldots ,x_{pi}^{\left(k\right)},y_{i}^{\left(k\right)}\right)}^{⊤}{\}}_{i=1}^{n^{\left(k\right)}}$, where 1≤ *k*≤*K*. Here, $z_{i}^{\left(k\right)}$ represents the measurements on the *i*th individual at node *k*, where $y_{i}^{\left(k\right)}\in R$ denotes their response variable and ${\left[x_{1i}^{\left(k\right)},\ldots ,x_{pi}^{\left(k\right)}\right]}^{⊤}\in R^{p}$ denotes their covariate vector. The total sample size at node *k* is denoted by *n*^(*k*)^. The combined data set $D^{\left(1\right)},...,D^{\left(K\right)}$ make up the whole data set without any duplicated individuals, indicating that $\sum_{k=1}^{K}n^{\left(k\right)}=n$.

Throughout the analysis, it is assumed that each $z_{i}^{\left(k\right)}$ is independent across 1≤ *i*≤*n*^(^*^k^*^)^ and 1≤ *k*≤*K* and there are no missing data. In addition, the size of the covariate space (ie, the dimension of ${\left[x_{1i}^{\left(k\right)},\ldots ,x_{pi}^{\left(k\right)}\right]}^{⊤}$, which is equal to *p* representing the number of features to include as predictors in the GLM) is assumed to be low, eliminating the need for regularization or variable selection. Finally, it is assumed that each node possesses a nonnegligible proportion of the whole data set. Specifically, for each *k* ∈ {1, ... , *K*}, the quantity *n*^(*k*)^/*n* is bounded away from 0 and 1 as the sample size *n* tends to infinity, denoted as *n*^(*k*)^/*n* → *p*^(^*^k^*^)^ ∈ (0,1).

#### Mathematical Description of the GLM Framework

The formulation of the GLM considered in this paper encompasses various commonly used regression models, such as linear regression, logistic regression, Poisson regression, and probit models. It assumes that the density or probability mass function of each response variable (known as the random components) belongs to the exponential family of distributions. Within this formulation, the mean of the response variable is expressed as a function of a linear combination of the corresponding covariate vector. Formally, it assumes that there exist unknown parameters $\beta^{⋆}\in R^{p+1}$ and $\phi^{⋆}§gt;0$ and known model-specific functions *b*, *c*, *g*, and *h* such that, with $x_{i}^{\left(k\right)}={\left[x_{0i}^{\left(k\right)},x_{1i}^{\left(k\right)},\ldots ,x_{pi}^{\left(k\right)}\right]}^{⊤}$ and $x_{0i}^{\left(k\right)}=1$, $y_{i}^{\left(k\right)}|x_{i}^{\left(k\right)}∼f\left(\cdot \;;\;x_{i}^{\left(k\right)},\beta^{⋆},\phi^{⋆}\right)\;,$ where, for any $\beta ={\left[\beta_{0},\beta_{1},\ldots ,\beta_{p}\right]}^{⊤}\in R^{p+1}$ and ϕ,


(1) $$f\left(y;x_{i}^{\left(k\right)},\beta ,\phi \right)=exp\left[\frac{yh\left(\beta^{⊤}x_{i}^{\left(k\right)}\right)-b\left\{h\left(\beta^{⊤}x_{i}^{\left(k\right)}\right)\right\}}{\phi }+c\left(y,\phi \right)\right].$$

In formula 1, *b* is such that $b\prime \left\{h\left(\beta^{⊤}x_{i}^{\left(k\right)}\right)\right\}=E\left(y_{i}^{\left(k\right)}|x_{i}^{\left(k\right)}\right)=g^{\left(-1\right)}\left(\beta^{⊤}x_{i}^{\left(k\right)}\right)$, with *b*’(*x*)=∂*b*(*x*)/∂*x*. In this framework, *g* is called *link function*, the term $h\left(\beta^{⊤}x_{i}^{\left(k\right)}\right)$ is usually referred to as the *natural parameter*, and *b* is referred to as the *cumulant* function. ϕ is often called the *dispersion parameter* and is either known (eg, with ϕ=1) or unknown. When *h*(*x*)=*x* (ie, *h* is the identity function), the link *g* is called *canonical*.

The logistic regression model is obtained upon taking ϕ=1, *h*(*x*)=*x*, *b*(*x*)=log(1+ *e^x^*), $c\left(y,\phi \right)=0$, and *g*(*x*)=log{*x*/(1 – *x*)}. The linear regression model with homoscedastic residual error variance ϕ is derived upon setting *h*(*x*)=*x*, *b*(*x*)=*x*^2^/2, $c\left(y,\phi \right)=-y^{2}/\left(2\phi \right)-log\left(2\pi \phi \right)/2$, and *g*(*x*)=*x*. Hence, both the logistic and the linear regression models rely on a canonical link function in the exponential family distribution.

## Methods

### Methodology Related to Objective 1

#### Overview

Scoping reviews are well suited to efficiently map key concepts within a research area [6]. They are widely acknowledged for their ability to clarify working definitions and conceptual boundaries in a specific topic or field [7], facilitating a shared understanding among researchers regarding the status of the research area. These considerations make the scoping review methodology well designed to achieve objective 1.

Scoping studies use systematic searches of relevant databases, using specific keywords to define the boundaries of the research field. However, identifying these keywords can be challenging, particularly when relevant papers are scattered across different research streams or in independent clusters that do not reference each other. To address the risk of overlooking significant methodological contributions due to a limited number of keywords, a snowballing literature search was initially conducted to generate a comprehensive list of keywords related to HPSA. The scoping review then proceeded with a systematic literature search using the identified keywords. It is worth noting that, as the planning of the scoping review is independent of the search approach, the guidelines presented in the work by Arksey and O’Malley [6] are still appropriate.

#### Methodology Pertaining to the Snowballing Keyword Search

Snowballing is generally used as a literature search method aimed at identifying papers belonging to a given field [8]. It typically consists of three steps: (1) initiate searches in prominent journals or conference proceedings to gather an initial set of papers, (2) conduct a backward review by examining the reference lists of the relevant articles discovered in step 1 (continue iterating until no new papers are found), and (3) perform a forward search by identifying articles that cite the papers identified in the previous steps.

To avoid selection bias, the initial set of papers for the snowballing approach in step 1 is sometimes generated through a search in Google Scholar [9]. The latter strategy was used in this study, too.

As mentioned previously, in this review, the snowballing search strategy was used in preparation for the application of the scoping review protocol with the goal of identifying relevant keywords. Specifically, the starting set of papers was assembled by screening titles and abstracts from the first 50 papers generated through a Google Scholar search using the strings *distributed inference* and *federated inference*. The main inclusion criterion was “presents, applies or discusses a statistical inference method to analyse horizontally partitioned data.” The backward and forward snowballing step approaches were then applied.

From the set of keywords found in the selected papers, a list of those relevant to HPSA but not directly associated with any specific method was retained for the scoping review step. It is worth noting that, as the objective of this scoping review was to identify statistical inference methods for horizontally partitioned data, keywords linked to method identifiers had to be excluded from the retained list to avoid preselection bias in the scoping review phase of this project.

The selected keywords that were identified from the snowballing literature search were *distributed algorithms*, *distributed estimation*, *distributed inference*, *distributed learning*, *distributed regression*, *federated inference*, *federated estimation*, *federated learning*, *privacy-protecting algorithm*, *privacy-preserving algorithm*, and *aggregated inference*.

#### Methodology Pertaining to the Scoping Review

The scoping review methodological framework by Levac et al [10] (see also the work by Arksey and O’Malley [6]) was followed. The steps are briefly described in this section. A detailed protocol is available in Multimedia Appendix 1 [2,4,6,10-19].

We conducted a comprehensive search across 4 bibliographic databases—MEDLINE, Scopus, MathSciNet, and zbMATH—to encompass the interdisciplinary nature of the topic and identify relevant research articles. Our search strategies were based on 2 key concepts: distributed data and statistical inference. In addition to the keywords obtained from the snowballing step, we incorporated terms such as *confidence interval* to target articles focusing specifically on statistical inference. To ensure the inclusion of recent advancements, our search was limited to papers published from 2000 onward. This cutoff date was chosen to account for the emergence of distributed data, the prevalence of massive data sets, and advancements in technology. It was set conservatively to capture any early developed methods and ensure comprehensive coverage of the topic.

After completing the primary search, a 2-stage selection process was used. Initially, 2 authors (MPD and FCL) collaborated to screen all articles identified through the search strategy based on their titles and abstracts. Subsequently, the full texts of the selected articles were independently reviewed by both authors to finalize the selection. This rigorous approach ensured a thorough evaluation of each article’s relevance and eligibility for inclusion.

The primary inclusion criterion for the selection process was as follows: *presents a solution for conducting inferential statistics on horizontally partitioned data*. This criterion was used to ensure that the chosen articles specifically addressed methods associated with performing statistical inference on horizontally partitioned data.

The following exclusion criteria were derived directly from objective 1: (1) does not address inferential statistics, including CIs, hypothesis testing, or asymptotic normality; (2) does not provide a methodological contribution; and (3) presents a solution for encryption or secret sharing.

To ensure the inclusion of validated approaches, the selection process only considered published papers that had full-text availability in English or French. Discussion papers were excluded as they do not present novel methods or approaches.

Exclusion was considered if any of the exclusion criteria were met or if any of the inclusion criteria were not met.

Finally, the references of each included article from the databases were assessed to identify any relevant articles that may not have been captured during the initial screening due to specific keywords. This additional step in the selection process was necessary given the broad range of vocabulary used to describe applicable approaches in our context.

Data extraction for the included articles was conducted by one author (MPD) and followed a collectively developed data-charting form. Model type (*parametric regression*, *semiparametric regression*, *nonparametric regression*, or *not specific to regression*) and number of communications from the CC to the nodes (0 or ≥1) were among the data extracted. All methods from the included articles were subsequently classified according to their specified characteristics, as outlined in the protocol. In addition, as part of the analysis, we conducted a screening of the general distributed approaches commonly used across all specific methods.

### Methodology Related to Objective 2

#### Overview

To achieve objective 2, a total of 3 steps were taken. First, we identified methodological approaches from articles included in this scoping review that enable parameter and CI estimations from horizontally partitioned data within a standard GLM framework. Methods designed specifically for the particular cases of linear or logistic regression were also reported but were not analyzed in detail. Second, we extracted workflows for each approach to determine the information exchanged between data storage nodes and the CC. Third, we analyzed the mathematical assumptions necessary for parameter estimation and the consistency of CI procedures. We specifically reported the assumptions related to the distribution of node-specific covariates.

#### Identification of the Approaches

To identify approaches that enabled the fitting of any GLM using horizontally partitioned data, 2 authors (FCL and MPD) independently assessed all articles included in this scoping review. The reviewers specifically looked for articles that discussed approaches applicable to the GLM class described in the *Mathematical Foundations and Notation* section, including likelihood-based methods, M-estimation, and estimating equations. In addition, we identified and reported articles that specifically focused on regression settings for linear or logistic regression. However, unless the method described was considered easily adaptable to the GLM framework, these articles were not retained for detailed analysis.

A method was selected if it provided an algorithm for fitting GLMs using horizontally partitioned data, aligning with the characteristics outlined in the *Mathematical Foundations and Notation* section. In cases in which an article presented asymptotic normality results for the estimators but did not provide an estimator for the asymptotic variance-covariance matrix, the article was still retained, and an estimator for the asymptotic variance was derived using the available calculated quantities.

As our GLM framework assumes no missing values, low dimensionality, and a small number of nodes relative to the total sample size, any terms related to these specific conditions mentioned in an article’s methodology were disregarded. Consequently, the calculations for CIs were adjusted accordingly. If an article solely focused on one of these aspects without contributing to the overall methodology, it was not included in the final selection.

Methodological components regarding parameter estimation and CI procedures were extracted from the screened articles. Specifically, the focus was on understanding how parameters should be estimated within a horizontally partitioned framework and how CIs should be computed for these parameters. For each article, the formulas related to quantities shared among the nodes and quantities calculated by the CC were derived and analyzed. These formulas were examined within a workflow that indicated the necessary circulation of information for the procedure to be executed. The derived workflows constituted the first part of our defined unified framework for HPSA approach comparisons in GLM settings.

For the reported results, the rationale behind each method that was deemed suitable for fitting GLMs was documented, along with the corresponding reference to the paper included in this scoping review in which the method was introduced or discussed.

Articles that discussed approaches specifically applicable to the cases of linear or logistic regression were also mentioned but not elaborated on in detail.

### Methodology Related to Objective 3

In most statistical settings with horizontally partitioned data, it is commonly assumed that the sample sizes of the data nodes are equal and that the distribution of covariates is the same across all nodes. However, when the number of nodes is fixed and relatively small compared to the sample sizes, it is possible to adapt a particular approach to handle situations in which the sample sizes and covariate distributions vary across nodes. This can be achieved by combining the theoretical arguments presented in the original article on the method in question with the principles of asymptotic statistics theory concerning maximum likelihood estimation.

To adapt a given approach for situations in which sample sizes and covariate distributions differ across nodes, the following steps were taken:

1. The formulas for the relevant quantities were modified to emphasize the changes caused by this scenario. It was ensured that the adapted quantities were equivalent to their counterparts presented in the original article for an equal sample size setting.
2. Using asymptotic theory, an asymptotic normality result was derived for the estimators of interest considering a set of assumptions that accommodated potential variations in sample sizes and covariate sampling distribution across nodes while still enabling meaningful theoretical arguments.
3. Formulas for the asymptotic variances were derived. Statistical theory on maximum likelihood estimation was used to obtain consistent estimators for asymptotic variances. The latter estimators were derived under the constraint that they had to be calculated without requiring any additional communication round between the CC and the nodes. Thus, throughout the adaptation process, the communication workflow remained unchanged compared to the original method.

These steps ensured the mathematical correctness of adapting the approaches to handle different sample sizes and covariate distributions across nodes. Importantly, these adaptations maintained consistency with the original method’s communication workflows. To adapt and compare the methods based on their information sharing requirements, common assumptions and a unified mathematical and algorithmic notation were necessary. Ultimately, these assumptions and the notation, along with the workflow types derived for objective 2, enabled us to establish a unified notational framework for approach comparisons when performing HPSA based on GLMs in the context of health analytics.

We describe the statistical estimates of interest. A standard GLM typically includes 1 or 2 unknown parametric components. The first are the ***β*** parameters, which are commonly assumed to be unknown. The second component is the nuisance parameter ϕ, which can be either known (eg, in logistic models) or unknown (eg, in linear models). In practical applications, when ϕ is unknown, its estimated value is often not the main focus, although the latter is necessary to estimate the asymptotic variance of the ***β*** parameter estimates.

In the upcoming analysis, we will assume that the parameter ϕ is unknown and estimated using the recommended approach in the selected methods. However, in cases in which ϕ is known, the process becomes simpler. This involves substituting the known value of ϕ and disregarding the estimation step. It is important to highlight that estimating ϕ requires additional information to be shared between the nodes and the CC but it does not necessitate any extra communication round between them.

The estimation processes for both the ***β*** and ϕ parameters are discussed. In addition, we explain how to compute an estimator for the asymptotic variance specifically for the estimator of $\beta^{⋆}$. It is important to note that the results presented in the following section can be modified and extended to develop a similar procedure for estimating $\phi^{⋆}$.

Using these results, based on an estimator of ***β*** (eg, $\hat{\beta }$) and a formula for the estimator of the asymptotic variance-covariance matrix involved in its associated asymptotic normality result (eg, $\hat{\Sigma }$), Wald-type (1–α) CIs can be computed for each component of $\beta^{⋆}$ using the following formula:


$${\left[\hat{\beta }\right]}_{j}\pm z_{1-\alpha /2}\sqrt{{\left[\hat{\Sigma }\right]}_{jj}/n}\;\;\;\text{for }\;j\in \left\{1,...,p+1\right\}$$

Regarding the reported results, for each approach considered, we present the formulas necessary to compute the final estimates of the ***β*** parameters and their corresponding CIs. The presentation of these formulas was designed to emphasize the communication workflow. Furthermore, a comprehensive algorithm is provided outlining the step-by-step process.

In addition, the asymptotic normality of the ***β*** parameter estimators is stated accompanied by the formula for the asymptotic variance and its consistent estimator. Detailed proofs for these results can be found in Multimedia Appendix 2 [20-22].

## Results

### Results Related to Objective 1

#### Search Outcomes From the Scoping Review

**Figure 1.**
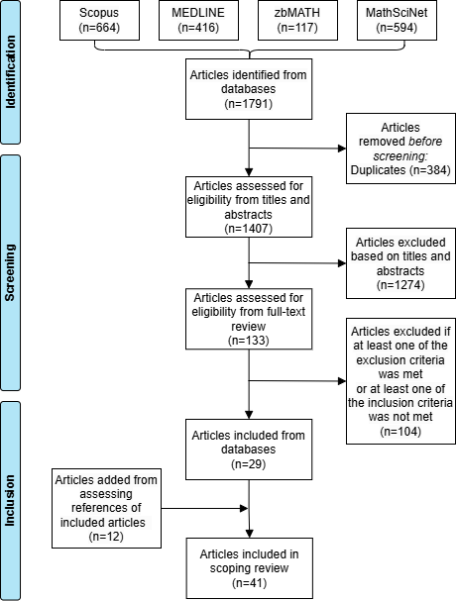
Article selection process for the scoping review. Detailed inclusion and exclusion criteria are described in the text and in the protocol.

As presented in Figure 1, a total of 1407 articles were initially identified across all 4 databases after removing duplicates. Subsequently, most of these articles (1274/1407, 90.55%) were excluded based on the evaluation of titles and abstracts, leaving 9.45% (133/1407) of the articles for eligibility assessment through full-text review. Following this assessment, 29 articles were included from the databases. In addition, by reviewing the references of the included articles, 12 more articles were identified and added to the study.

Regarding the additional 12 articles obtained through the assessment of references of the included articles, it was observed that most of them did not mention statistical inference or related terms in their abstracts [2,11,23]. Consequently, these articles were not captured in the initial database search results. Furthermore, some articles directly referred to the specific method used without including any keywords related to horizontally partitioned data in their abstracts or titles [24,25], which greatly reduced the chance of initially identifying them. However, during the process of reviewing the references of the included articles, all the relevant papers that were initially identified through the snowballing strategy were eventually retrieved either through the search strategy or the selection process based on the references of the included articles.

#### Results of the Scoping Review

Each article included in this scoping review put forth one or multiple methodological approaches pertaining to objective 1. The similarities and differences regarding the communication schemes involved and their background of origin are summarized in this section.

**Figure 2.**
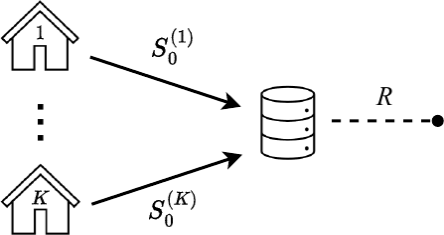
Workflow I: each node calculates summary statistics from its own samples. Results are sent to the coordinating center, which combines the information provided by each node to produce the final estimates.

**Figure 3.**
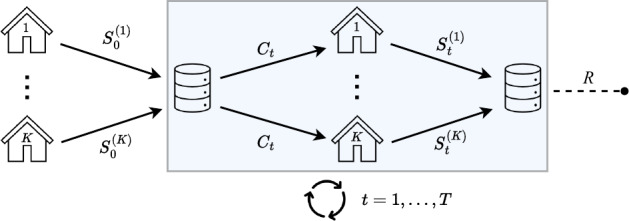
Workflow II: multiple communication rounds are allowed between the coordinating center and the data storage nodes.

**Figure 4.**
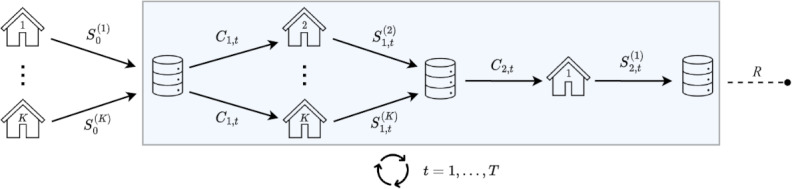
Workflow III: multiple communication rounds are allowed between the coordinating center and the data storage nodes, with node 1 following a distinct communication pattern compared to the other nodes.

**Figure 5.**
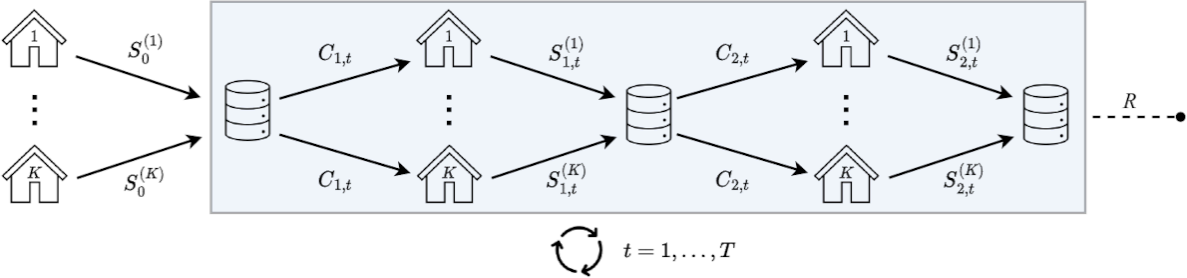
Workflow IV: multiple communication rounds are allowed between the coordinating center (CC) and the data storage nodes, with 2 back-and-forth distinct communication exchanges between each node and the CC at each iteration.

First, all the selected articles discussed one or more statistical procedures that operate on horizontally partitioned data using one of the communication schemes depicted in Figures 2-5.

In workflow I, as shown in Figure 2, each node calculates summary statistics from its own samples, and the results are sent to the CC. The CC combines the information provided by each node to produce the final estimates. This communication approach is commonly referred to as “one-shot” or “noniterative” in the literature, although not always consistently.

In workflow II, as shown in Figure 3, multiple communication rounds are allowed between the CC and the data storage nodes. This allows for iterative interactions between the nodes and the CC to refine the estimates.

Some approaches fundamentally differ from the 2 previous workflows by assigning a different role to one of the nodes (eg, node 1) compared to the others. These approaches operate using workflow III, as illustrated in Figure 4, where node 1 follows a distinct communication pattern compared to the other nodes. In the papers included in this scoping review that discussed these approaches, node 1 was invariably designated as the CC. However, in the context of this paper, their roles were differentiated. The additional step performed by the CC, which involves data aggregation, can be particularly well suited for privacy protection purposes in practice.

The particular setting shown in workflow IV in Figure 5 requires 2 back-and-forth communication exchanges between each node and the CC at each iteration. This communication pattern distinguishes this workflow from the others.

In light of the preceding discussion, from an operational standpoint, 2 categories of workflows emerge. On the one hand, there are workflows that do not necessitate any communication from the CC to the nodes, which are captured in workflow I. On the other hand, there are workflows that involve one or more communication exchanges from the CC to the nodes, which are captured in workflows II, III, and IV.

To emphasize similarities among the methods presented in the articles in this review and facilitate the identification of methods suitable for specific purposes, a systematic classification is presented in Table 1. The articles are categorized based on the type of models used and the number of communications from the CC to the individual nodes.

**Table 1.** Classification of the articles included in this scoping review.

Type of model	0 communication from CC^a^ to nodes	≥1 communication exchange from CC to nodes
Parametric regression	Basiri et al [26] Battey et al [27] Fan et al [28] Guo et al [29] Chen and Xie [25] Lin and Xi [30] Rosenblatt and Nadler [23] Zhang et al [31] Chang et al [32] Wu et al [33] Hector and Song [34]	Huang and Huo [12] Jordan et al [13] Mozafari-Majd and Koivunen [35,36] Yue et al [37] Duan et al [38] Duan et al [2] Tong et al [39] Di et al [40] Edmondson et al [41] Luo and Li [42] Shu et al [43]
Semiparametric regression	Zhao et al [44] Park et al [14]	Luo et al [45] Duan et al [11]
Nonparametric regression	Liu et al [46] Zhang et al [47] Volgushev et al [48]	Wang et al [49]
Not specific to regression	Atta-Asiamah and Yuan [50] Minsker [51] Lin and Xi [52] Bruce et al [53] Chen and Peng [54] Nezakati and Pircalabelu [55] Banerjee et al [24] Shi et al [56] Wu et al [57]	Lai et al [58]

a CC: coordinating center.

Most of the methods were published the big or massive data and multi-machine methodological setting, whereas some were reported within the context of health analytics. Within the big or massive data and multi-machine methodological setting, many methods involved an initial step of random data partitioning among multiple machines. However, certain methods assumed a scenario in which data were already stored on separate machines, as observed in the studies by Fan et al [28] and Jordan et al [13]. Furthermore, it is worth noting that no articles published before 2010 were included, aligning with our initial hypothesis regarding the identification of contemporary methodological settings. Most of the included articles (30/41, 73%) were published after the year 2018.

Most articles (33/41, 81%) addressed a setting in which a CC existed external to the nodes, as exemplified by articles such as those by Lin and Xi [52], Volgushev et al [48], and Yue et al [37]. In contrast, as mentioned previously, some studies (8/41, 19%) designate one of the nodes to assume this central role, as demonstrated in the study by Chang et al [32].

The methods identified through our search strategy shared a common characteristic of using a global model that incorporated population-level parameters. In some cases, these parameters may also include node-specific components to accommodate node-specific statistical heterogeneity in the outcome-predictors relationship, which captures deviations from the population-level conditional probability distribution of the outcome given the predictors.

A few of the reported methods had the capability to yield results identical to those obtained if the individual line data were pooled from all nodes [33,43].

While most articles (31/41, 76%) featured methods related to regression models, including semiparametric and nonparametric designs, a few (10/41, 24%) reported results for other modeling frameworks. These included methods for M-estimation, U-statistics, symmetrical statistics, and natural parameter estimation, some of which encompassed regression models as a specific instance.

### Results Related to Objective 2

In total, 6 approaches were selected as applicable to the standard GLM framework. They all assumed that nodes had equal sample sizes and identical distributions for the covariates.

#### Simple Averaging

One of the simplest methods for horizontally partitioned data analysis, often referred to as the “simple averaging method” or the “divide-and-conquer” approach, has been extensively studied in the literature, for example, the studies by Zhang et al [31] and Shamir et al [59], which were included in our scoping review. This method operates through workflow I in Figure 2. In this approach, node-level model estimates are gathered and averaged at the CC to generate the final estimates.

In the context of GLM, each node is initially tasked with calculating the maximum likelihood estimator (MLE) of the $\beta^{⋆}$ and $\phi^{⋆}$ parameters using their respective data. In addition, the Hessian matrix of the log-likelihood function with respect to the ***β*** parameters must be computed for constructing Wald-type CIs. The estimated parameters and the computed Hessian matrix are then transmitted to the CC.

The final parameter estimates of are obtained by averaging the node-specific estimates while the local Hessians and estimates of $\phi^{⋆}$ are used to compute an estimator for the asymptotic variance.

#### Single Distributed Newton-Raphson Updating

The single distributed Newton-Raphson updating method is an iterative procedure that includes an additional communication round between the CC and the nodes compared to the simple averaging method. It was originally proposed as the “distributed one-step” method in the study by Beyan et al [60], but in this study, it is referred to by a different term to avoid any confusion regarding communication complexity. This method operates using workflow II, as depicted in Figure 3, with *t*=1 (where T represents the number of cycles in the iteration scheme). It enhances the simple averaging estimators by incorporating a single distributed Newton-Raphson updating step.

In the context of GLM, each node first calculates the MLE of $\beta^{⋆}$ and $\phi^{⋆}$ and transmits them to the CC. The CC aggregates these estimates using averaging and sends the result back to the nodes. The nodes then compute the gradient and the Hessian matrix of the log-likelihood function, evaluated at the received $\beta^{⋆}$ and $\phi^{⋆}$ estimates. Subsequently, the gradient and the Hessian matrix are sent back to the CC, which averages them and computes a Newton-Raphson updating step based on the simple averaging estimates. An estimator for the asymptotic variance can be calculated by using the received Hessian matrices and the updated estimate of $\phi^{⋆}$.

#### Multiple Distributed Newton-Raphson Updatings

The multiple distributed Newton-Raphson updating method leverages the fact that, for standard GLMs, the algorithm typically used to calculate the MLE of $\beta^{⋆}$ and $\phi^{⋆}$ in a centralized pooled setting can be executed in a distributed manner without any loss of information. This is possible because the algorithm relies on Newton-Raphson updatings (or, sometimes, Fisher scoring updatings) that are expressed using 2 sums of node-specific summary statistics, namely, local gradients and local Hessian matrices of the log-likelihood function, evaluated at the $\beta^{⋆}$ and $\phi^{⋆}$ estimates from the previous iteration. A version of this method is proposed in the study by Wu et al [33] under the logistic regression framework. It operates through workflow II in Figure 3 for a general *T*≥1.

#### Distributed Estimating Equation

The class of estimating equation estimators is vast and encompasses a broad range of statistical estimation techniques, including likelihood-based approaches that rely on searching for critical points. The fundamental idea behind estimating equation methods is to establish a system of equations that involve both the sample data and the unknown model parameters. These equations are then solved to determine the parameter estimates. MLEs, which are obtained by setting the gradient of the log-likelihood function with respect to the unknown parameters equal to 0, belong to the class of estimating equation estimators.

The distributed estimating equation approach involves gathering summary statistics from nodes at the CC level, enabling the reconstruction of the estimating equations, or, more commonly, an approximation of them that would have been obtained in a pooled centralized setting. This method is discussed in the study by Lin and Xi [30] and operates using workflow I, as depicted in Figure 2.

In the context of GLMs, the distributed estimating equation approach involves initially assigning each node the task of computing and sending their local MLEs and the Hessian matrix of their local log-likelihood, evaluated at those MLEs, to the CC. The CC uses these received quantities to reconstruct the global estimating equations or an approximation thereof. This reconstruction ultimately leads to an analytical solution for obtaining the resulting estimates. CIs are computed using a combination of the Hessian matrices and the final estimator of $\phi^{⋆}$.

It is important to note that, when this approach is applied in the context of linear regression, it enables the acquisition of $\beta^{⋆}$parameter estimates that are identical to those obtained in a pooled centralized setting.

#### Distributed Estimation Using a Single Gradient-Enhanced Log-Likelihood

This method differs fundamentally from the ones discussed thus far as it involves a distinct role for one particular node in obtaining model parameter estimates. It operates using workflow III, as depicted in Figure 4, and was proposed in the study by Jordan et al [13] under the name “Surrogate likelihood.” This approach relies on an approximation of the global likelihood by viewing it as an analytic function. It expands the global likelihood into an infinite series around an initial guess ${\hat{\beta }}_{\text{SGE},\text{0}}$ and replaces the higher-order derivatives (order of ≥2) of the global likelihood with those of a Taylor expansion of a node’s (eg, node *k*=1) local likelihood around the same value. By following this procedure, the so-called surrogate likelihood can be solved using data from node *k*=1and aggregated gradients from nodes *k* ∈ {2, ..., *K*}.

In the context of GLM, the CC first collects the necessary information to compute initial estimates for the parameters $\beta^{⋆}$ and $\phi^{⋆}$. These initial estimates can be obtained through various approaches, such as a simple averaging estimator or the MLEs computed using data from node 1. These initial estimates are then transmitted to nodes *k* ∈ {2, ..., *K*}. Each of these nodes calculates the gradient of the log-likelihood function, evaluated at the received estimates, and sends it back to the CC. The CC averages these gradients and sends the result to node 1. Node 1 solves a gradient-enhanced log-likelihood using its own data and the received average gradient. The resulting estimate is sent back to the CC as the final estimate. To compute CIs, each node must send the Hessian matrix of its local log-likelihood function, evaluated at the initial received estimate.

The steps related to estimation can be repeated multiple times.

#### Distributed Estimation Using Multiple Gradient-Enhanced Log-Likelihoods

This method is in the spirit of the *distributed estimation using a single gradient-enhanced log-likelihood* approach described previously except that all nodes have to solve a gradient-enhanced log-likelihood instead of only one of them. Results pertaining to statistical inference are discussed in the study by Fan et al [28] under a penalized setting. A nonpenalized version of this method was introduced in the study by Shamir et al [59], although the latter did not discuss CIs or hypothesis testing and, hence, was not included in our scoping review. It operates through workflow IV depicted in Figure 5.

The following subsection presents the 6 approaches described in objective 2 within a unified notational framework that accounts for the peculiarities commonly encountered in health analytics. Algorithms were derived using this common notation to rigorously describe the methods and enable their comparison. Theoretical results regarding the estimators involved are detailed and proven in Multimedia Appendix 2. While this section is necessary to increase trust in HPSA methods by transparently showing precisely what information is shared with the CC through comprehensive mathematical formulas, the summary provided in Table 2 suffices for a high-level understanding of the overall picture.

**Table 2.** Quantities shared in each adapted method’s communication workflow.

Method number	Method	Exchanged quantities from nodes to CC^a^		Exchanged quantities from CC to nodes
		$S_{0}^{\left(k\right)}$	$S_{t}^{\left(k\right)}$, *t*≥1	*C_t_*
1	Simple averaging	${\hat{\beta }}_{\text{MLE}}^{\left(k\right)}$; ${\hat{\phi }}_{\text{MLE}}^{\left(k\right)}$; $V_{\text{MLE}}^{\left(k\right)}$	—^b^	—
2	Single distributed Newton-Raphson updating	${\hat{\beta }}_{\text{MLE}}^{\left(k\right)}$; ${\hat{\phi }}_{\text{MLE}}^{\left(k\right)}$	$D_{\text{NR},\text{1}}^{\left(k\right)}$; $V_{\text{NR},\text{1}}^{\left(k\right)}$; $E_{\text{NR},\text{1}}^{\left(k\right)}$; $F_{\text{NR},\text{1}}^{\left(k\right)}$	${\hat{\beta }}_{\text{NR},\text{0}}$; ${\hat{\phi }}_{\text{NR},\text{0}}$
3	Multiple distributed Newton-Raphson updatings (with *T* Newton-Raphson updatings)	${\hat{\beta }}_{\text{MLE}}^{\left(k\right)}$; ${\hat{\phi }}_{\text{MLE}}^{\left(k\right)}$	$D_{\text{NR},\text{t}}^{\left(k\right)}$; $V_{\text{NR},\text{t}}^{\left(k\right)}$; $E_{\text{NR},\text{t}}^{\left(k\right)}$; $F_{\text{NR},\text{t}}^{\left(k\right)}$	${\hat{\beta }}_{\text{NR},\text{t-1}}$; ${\hat{\phi }}_{\text{NR},\text{t-1}}$
4	Distributed estimating equations	${\hat{\beta }}_{\text{MLE}}^{\left(k\right)}$; ${\hat{\phi }}_{\text{MLE}}^{\left(k\right)}$; $V_{\text{MLE}}^{\left(k\right)}$; $F_{\text{MLE}}^{\left(k\right)}$	—	—
5	Distributed single gradient-enhanced log-likelihood	${\hat{\beta }}_{\text{MLE}}^{\left(k\right)}$; ${\hat{\phi }}_{\text{MLE}}^{\left(k\right)}$	Nodes 2 to *K*: $D_{\text{SGE},\text{1}}^{\left(k\right)}$, $V_{\text{SGE},\text{1}}^{\left(k\right)}$, and $E_{\text{SGE},\text{1}}^{\left(k\right)}$; node 1: ${\hat{\beta }}_{\text{SGE},\text{1}}$, ${\hat{\phi }}_{\text{SGE},\text{1}}$, and $V_{\text{SGE},\text{1}}^{\left(1\right)}$	${\hat{\beta }}_{\text{SGE},\text{0}}$; ${\hat{\phi }}_{\text{SGE},\text{0}}$; to node 1 only: $D_{\text{SGE},\text{1}}$ and $E_{\text{SGE},\text{1}}$
6	Distributed multiple gradient-enhanced log-likelihood	${\hat{\beta }}_{\text{MLE}}^{\left(k\right)}$; ${\hat{\phi }}_{\text{MLE}}^{\left(k\right)}$	$D_{\text{MGE},\text{1}}^{\left(k\right)}$; $V_{\text{MGE},\text{1}}^{\left(k\right)}$; $E_{\text{MGE},\text{1}}^{\left(k\right)}$; ${\hat{\beta }}_{\text{MGE},\text{1}}^{\left(k\right)}$; ${\hat{\phi }}_{\text{MGE},\text{1}}^{\left(k\right)}$	${\hat{\beta }}_{\text{MGE},\text{0}}$; ${\hat{\phi }}_{\text{MGE},\text{0}}$; ${\bar{D}}_{\text{MGE},\text{1}}$; ${\bar{E}}_{\text{MGE},\text{1}}$

a CC: coordinating center.

b No exchanged quantities.

### Results Related to Objective 3

#### Notation for Shared Quantities

In what follows, let the log-likelihood of the data stored in node *k* (using *D*^(*k*)^) be denoted by


$$l^{\left(k\right)}\left(\beta ,\phi \right)=\frac{1}{n^{\left(k\right)}}\sum_{i=1}^{n^{\left(k\right)}}\left\{\frac{y_{i}^{\left(k\right)}h\left(\beta^{⊤}x_{i}^{\left(k\right)}\right)-b\left\{h\left(\beta^{⊤}x_{i}^{\left(k\right)}\right)\right\}}{\phi }+c\left(y_{i}^{\left(k\right)},\phi \right)\right\}.$$

In addition, let **D**^(*k*)^ (***β***) ∈ R *^p^*^+1^ be such that


(2) $$D^{\left(k\right)}\left(\beta \right)=\frac{1}{n^{\left(k\right)}}\sum_{i=1}^{n^{\left(k\right)}}x_{i}^{\left(k\right)}h\prime \left(\beta^{⊤}x_{i}^{\left(k\right)}\right)\left[y_{i}^{\left(k\right)}-b\prime \left\{h\left(\beta^{⊤}x_{i}^{\left(k\right)}\right)\right\}\right]$$

and define the (*p*+1)×(*p*+1) matrix **V**^(*k*)^ (***β***) as


(3) $$\begin{array}{l} V^{\left(k\right)}(\beta )=\frac{1}{n^{\left(k\right)}}\sum_{i=1}^{n^{\left(k\right)}}x_{i}^{\left(k\right)}{\left(x_{i}^{\left(k\right)}\right)}^{⊤}\left(h^{\prime }{\left(\beta^{⊤}x_{i}^{\left(k\right)}\right)}^{2}\;b^{\prime \prime }\{h\left(\beta^{⊤}x_{i}^{\left(k\right)}\right)\}\;-h^{\prime \prime }\left(\beta^{⊤}x_{i}^{\left(k\right)}\right)\left[y_{i}^{\left(k\right)}-b^{\prime }\{h\left(\beta^{⊤}x_{i}^{\left(k\right)}\right)\}\right]\right). \end{array}$$

As $D^{\left(k\right)}\left(\beta \right)=\phi \nabla_{\beta }l^{\left(k\right)}\left(\beta ,\phi \right)$, solving the equation $D^{\left(k\right)}\left(\beta \right)=0$ yields the node-specific MLE of ***β***, denoted hereafter using ${\hat{\beta }}_{\text{MLE}}^{\left(k\right)}$. The matrix $V^{\left(k\right)}\left(\beta \right)$ is equal to $-\nabla_{\beta }D^{\left(k\right)}\left(\beta \right)$ and relates to the Fisher information matrix through the equation $V^{\left(k\right)}\left(\beta \right)=-\phi \nabla_{\beta }^{2}l^{\left(k\right)}\left(\beta ,\phi \right)$.

Finally, set


(4) $$E^{\left(k\right)}\left(\phi ,\beta \right)=\frac{1}{n^{\left(k\right)}}\sum_{i=1}^{n^{\left(k\right)}}\left[y_{i}^{\left(k\right)}h\left(\beta^{⊤}x_{i}^{\left(k\right)}\right)-b\left\{h\left(\beta^{⊤}x_{i}^{\left(k\right)}\right)\right\}\right]-\frac{\phi^{2}}{n^{\left(k\right)}}\sum_{i=1}^{n^{\left(k\right)}}\frac{\partial }{\partial \phi }c\left(y_{i}^{\left(k\right)},\phi \right)$$

and


(5) $$F^{\left(k\right)}\left(\phi \right)=\frac{2\phi }{n^{\left(k\right)}}\sum_{i=1}^{n^{\left(k\right)}}\frac{\partial }{\partial \phi }c\left(y_{i}^{\left(k\right)},\phi \right)+\frac{\phi^{2}}{n^{\left(k\right)}}\sum_{i=1}^{n^{\left(k\right)}}\frac{\partial^{2}}{\partial \phi^{2}}c\left(y_{i}^{\left(k\right)},\phi \right).$$

Because $E^{\left(k\right)}\left(\phi ,\beta \right)=-\phi^{2}\left(\partial /\partial \phi \right)l^{\left(k\right)}\left(\beta ,\phi \right)$, when ϕ is unknown, solving the equation $E^{\left(k\right)}\left(\phi ,{\hat{\beta }}_{\text{MLE}}^{\left(k\right)}\right)=0$ for ϕ yields its node-specific MLE of $\phi^{⋆}$. We have $F^{\left(k\right)}\left(\phi \right)=-(\partial /\partial \phi )E^{\left(k\right)}\left(\phi ,\beta \right)$.

#### Simple Averaging

The simple averaging method follows upon execution of algorithm 1 (Textbox 1). First, each data node computes their local maximum by solving successively $D^{\left(k\right)}\left(\beta \right)=0$ and $E^{\left(k\right)}\left(\phi ,{\hat{\beta }}_{\text{MLE}}^{\left(k\right)}\right)=0$. To compute the CIs at the CC level, the entries of the (*p*+1)×(*p*+1) matrix $V_{\text{MLE}}^{\left(k\right)}=V^{\left(k\right)}\left({\hat{\beta }}_{\text{MLE}}^{\left(k\right)}\right)$ have to be computed from formula 3 with $\beta ={\hat{\beta }}_{\text{MLE}}^{\left(k\right)}$. Then, the set


(6) $$S_{0}^{\left(k\right)}=\left\{{\hat{\beta }}_{MLE}^{\left(k\right)},{\hat{\phi }}_{\text{MLE}}^{\left(k\right)},V_{\text{MLE}}^{\left(k\right)}\right\}$$

is sent to the CC. The parameter estimates are then aggregated by the CC through averaging. Specifically, the CC computes


(7) $${\hat{\beta }}_{\text{SA}}=\sum_{k=1}^{K}w^{\left(k\right)}{\hat{\beta }}_{\text{MLE}}^{\left(k\right)}\;\text{and}\;{\hat{\phi }}_{\text{SA}}=\sum_{k=1}^{K}w^{\left(k\right)}{\hat{\phi }}_{\text{MLE}}^{\left(k\right)},$$

where *w*^(*1*)^, ..., *w*^(*K*)^ are weights (ie, *w*^(^*^k^*^)^≥0 and $\sum_{k=1}^{K}w^{\left(k\right)}=1$) used to combine each node’s contribution. Often, weights can be taken proportional to local sample sizes, leading to the choice *w*^(*k*)^=*n*^(*k*)^/*n*.

Textbox 1. **Algorithm 1—simple averaging inference procedure.**

Input at the coordinating center (CC) level: Weight *w*^(*1*)^, ..., *w*^(*K*)^ attributed to each node’s contribution

Step required from each node *k* ∈ {1, …, *K*}:

Using data in $D^{\left(k\right)}$, compute the following quantities:

- MLE ${\hat{\beta }}_{\text{MLE}}^{\left(k\right)}$ of $\beta^{⋆}$ by solving $D^{\left(k\right)}\left(\beta \right)=0$;
- MLE $\phi_{\text{MLE}}^{\left(k\right)}$ of $\phi^{⋆}$ by solving $E^{\left(k\right)}\left(\phi ,{\hat{\beta }}_{\text{MLE}}^{\left(k\right)}\right)=0$;
- $V_{\text{MLE}}^{\left(k\right)}=V^{\left(k\right)}\left({\hat{\beta }}_{\text{MLE}}^{\left(k\right)}\right)$ using formula (3) with $\beta ={\hat{\beta }}_{\text{MLE}}^{\left(k\right)}$

*Send to the CC:*$S_{0}^{\left(k\right)}=\left\{{\hat{\beta }}_{\text{MLE}}^{\left(k\right)},{\hat{\phi }}_{\text{MLE}}^{\left(k\right)},V_{\text{MLE}}^{\left(k\right)}\right\}$.

Step required from the CC:

Using the received sets of quantities $S_{0}^{\left(1\right)},\ldots ,S_{0}^{\left(K\right)}$, calculate

- The simple averaging estimators ${\hat{\beta }}_{\text{SA}}=\sum_{k=1}^{K}w^{\left(k\right)}{\hat{\beta }}_{\text{MLE}}^{\left(k\right)}$ and ${\hat{\phi }}_{\text{SA}}=\sum_{k=1}^{K}w^{\left(k\right)}{\hat{\phi }}_{\text{MLE}}^{\left(k\right)}$;
- The estimator of the variance-covariance matrix ${\hat{\Sigma }}_{SA}={\hat{\phi }}_{\text{SA}}\sum_{k=1}^{K}\frac{nw^{\left(k\right)2}}{n^{\left(k\right)}}{\left(V_{\text{MLE}}^{\left(k\right)}\right)}^{-1}$

Output from the CC:

Final estimates: $R=\left\{{\hat{\beta }}_{\text{SA}},{\hat{\Sigma }}_{\text{SA}}\right\}$

Wald-type CIs for $\beta^{⋆}$ can be constructed based on the fact that the sequence $\sqrt{n}\left({\hat{\beta }}_{\text{SA}}-\beta^{⋆}\right)$ converges in distribution to a centered normal random variable with covariance matrix


$$\Sigma_{SA}=\phi^{⋆}\sum_{k=1}^{K}\frac{w^{\left(k\right)2}}{p^{\left(k\right)}}{\left(T_{\beta^{⋆}}^{\left(k\right)}\right)}^{-1},\;\text{where}\;T_{\beta^{⋆}}^{\left(k\right)}=E\left\{V^{k}\left(\beta^{⋆}\right)\right\}.$$

As $T_{\beta^{⋆}}^{\left(k\right)}$ is consistently estimated using $V_{\text{MLE}}^{\left(k\right)}$ and $\phi^{⋆}$ by ${\hat{\phi }}_{\text{SA}}$, and as *p*^(*k*)^ can be estimated using *n*^(*k*)^/*n*, it follows that a consistent estimator for **Σ**_SA_ is given by


$${\hat{\Sigma }}_{SA}={\hat{\phi }}_{\text{SA}}\sum_{k=1}^{K}\frac{nw^{\left(k\right)2}}{n^{\left(k\right)}}{\left(V_{\text{MLE}}^{\left(k\right)}\right)}^{-1}.$$

The simple averaging final estimates are then given by


$$R=\left\{{\hat{\beta }}_{\text{SA}},{\hat{\Sigma }}_{\text{SA}}\right\}.$$

#### Single Distributed Newton-Raphson Updating

The single distributed Newton-Raphson updating method follows upon execution of algorithm 2 (Textbox 2) with *T*=1. First, the CC gathers summary statistics to compute the simple averaging estimators of $\beta^{⋆}$ and $\phi^{⋆}$ without their accompanying CI. Hence, for *k*∈{1, ..., *K*}, and with ${\hat{\beta }}_{\text{MLE}}^{\left(k\right)}$ as mentioned previously (equation 6), node *k* sends to the CC the quantities


(8) $$S_{0}^{\left(k\right)}=\left\{{\hat{\beta }}_{\text{MLE}}^{\left(k\right)},{\hat{\phi }}_{\text{MLE}}^{\left(k\right)}\right\},$$

and the CC uses them to compute ${\hat{\beta }}_{\text{SA}}$ and ${\hat{\phi }}_{\text{SA}}$ using the formulas in equation 7.

Textbox 2. **Algorithm 2—distributed Newton-Raphson updatings procedure.**

Input at the coordinating center (CC) level: Weight *w*^(1)^, …, *w*^(*K*)^ attributed to each node’s contribution

Step required from each node *k* ∈ {1, …, *K*}:

Using data in $D^{\left(k\right)}$, compute

- ${\hat{\beta }}_{\text{MLE}}^{\left(k\right)}$ by solving ***D***^(*k*)^(***β***)=0;
- ${\hat{\phi }}_{\text{MLE}}^{\left(k\right)}$ by solving $E^{\left(k\right)}\left(\phi ,{\hat{\beta }}_{\text{MLE}}^{\left(k\right)}\right)=0$

*Send to the CC:*$S_{0}^{\left(k\right)}=\left\{{\hat{\beta }}_{\text{MLE}}^{\left(k\right)},{\hat{\phi }}_{\text{MLE}}^{\left(k\right)}\right\}$.

Step required from the CC:

Using the received quantities $S_{0}^{\left(1\right)},\ldots ,S_{0}^{\left(K\right)}$:

- Calculate ${\hat{\beta }}_{\text{SA}}=\sum_{k=1}^{K}w^{\left(k\right)}{\hat{\beta }}_{\text{MLE}}^{\left(k\right)}$ and ${\hat{\phi }}_{\text{SA}}=\sum_{k=1}^{K}w^{\left(k\right)}{\hat{\phi }}_{\text{MLE}}^{\left(k\right)}$;
- Initialize ${\hat{\beta }}_{\text{NR},t=0}={\hat{\beta }}_{\text{SA}}$ and ${\hat{\phi }}_{\text{NR},t=0}={\hat{\phi }}_{\text{SA}}$.

Execute for *t*=1, ..., *T*:

*Step required from the CC:*

*Broadcast to nodes:* $C_{t}=\left\{{\hat{\beta }}_{\text{NR,t-1}},{\hat{\phi }}_{\text{NR,t-1}}\right\}$

*Step required from each node k* ∈ {1,…,*K*}:

Using data in *D*^(*k*)^ and quantities in *C_t_*, compute:

- $D_{\text{NR},\text{t}}^{\left(k\right)}$ using formula (2) with $\beta ={\hat{\beta }}_{\text{NR},\text{t-1}}$;
- $V_{\text{NR},\text{t}}^{\left(k\right)}$ using formula (3) with $\beta ={\hat{\beta }}_{\text{NR},\text{t-1}}$;
- $E_{\text{NR},\text{t}}^{\left(k\right)}$ using formula (4) with $\phi ={\hat{\phi }}_{\text{NR},\text{t-1}}$ and $\beta ={\hat{\beta }}_{\text{NR},\text{t-1}}$;
- $F_{\text{NR},\text{t}}^{\left(k\right)}$ using formula (5) with $\phi ={\hat{\phi }}_{\text{NR},\text{t-1}}$ and $\beta ={\hat{\beta }}_{\text{NR},\text{t-1}}$.

*Send to the CC:*$S_{t}^{\left(k\right)}=\left\{D_{\text{NR},\text{t}}^{\left(k\right)},V_{\text{NR},\text{t}}^{\left(k\right)},E_{\text{NR},\text{t}}^{\left(k\right)},F_{\text{NR},\text{t}}^{\left(k\right)}\right\}$.

*Step required from the CC:*

Using the quantities in $S_{t}^{\left(k\right)}$, compute

-
${\bar{D}}_{\text{NR},\text{t}}=\sum_{k=1}^{K}w^{\left(k\right)}D_{\text{NR},\text{t}}^{\left(k\right)}$

-
${\bar{V}}_{\text{NR},\text{t}}=\sum_{k=1}^{K}w^{\left(k\right)}V_{\text{NR},\text{t}}^{\left(k\right)}$

-
${\bar{E}}_{\text{NR},\text{t}}=\sum_{k=1}^{K}w^{\left(k\right)}E_{\text{NR},\text{t}}^{\left(k\right)}$

-
${\bar{F}}_{\text{NR},\text{t}}=\sum_{k=1}^{K}w^{\left(k\right)}F_{\text{NR},\text{t}}^{\left(k\right)}$

Using, ${\hat{\beta }}_{\text{NR},\text{t-1}}$, ${\hat{\phi }}_{\text{NR},\text{t-1}}$ and the aggregated quantities, update previous parameter estimates

-
${\hat{\beta }}_{\text{NR},\text{t}}={\hat{\beta }}_{\text{NR},\text{t-1}}+{\bar{V}}_{\text{NR},\text{t}}^{-1}{\bar{D}}_{\text{NR},\text{t}}$

-
${\hat{\phi }}_{\text{NR},\text{t}}={\hat{\phi }}_{\text{NR},\text{t}}+\frac{{\bar{E}}_{\text{NR},\text{t}}}{{\bar{F}}_{\text{NR},\text{t}}}$

Step required from the CC:

Compute ${\hat{\Sigma }}_{\text{NR}}={\left({\bar{V}}_{\text{NR},T}\right)}^{-1}\left\{{\hat{\phi }}_{\text{NR},T}\sum_{k=1}^{K}\frac{nw^{(k{)}^{2}}}{n^{\left(k\right)}}V_{\text{NR},T}^{\left(k\right)}\right\}{\left({\bar{V}}_{\text{NR},T}\right)}^{-1}$

Output from the CC:

Estimates $R=\left\{{\hat{\beta }}_{\text{NR},\text{T}},{\hat{\Sigma }}_{\text{NR}}\right\}$

For reasons of convenience that will become clear later, the notation ${\hat{\beta }}_{\text{NR},\text{0}}$ and ${\hat{\phi }}_{\text{NR},\text{0}}$ will be used instead of ${\hat{\beta }}_{\text{SA}}$ and ${\hat{\phi }}_{\text{SA}}$, respectively. In this notation, the set of values


$$C_{1}=\left\{{\hat{\beta }}_{\text{NR},\text{0}},{\hat{\phi }}_{\text{NR},\text{0}}\right\}$$

is broadcasted to data nodes, which are then tasked with computing and sending back the quantities


$$\begin{array}{c} S_{1}^{\left(k\right)}=\left\{D_{\text{NR},\text{1}}^{\left(k\right)},V_{\text{NR},\text{1}}^{\left(k\right)},E_{\text{NR},\text{1}}^{\left(k\right)},F_{\text{NR},\text{1}}^{\left(k\right)}\right\}\;, \end{array}$$

where, for any integer *t*≥1, one defines


(9) $$\begin{array}{l} D_{NR,t}^{\left(k\right)}=D^{\left(k\right)}({\hat{\beta }}_{NR,t-1})\\ V_{NR,t}^{\left(k\right)}=V^{\left(k\right)}({\hat{\beta }}_{NR,t-1})\\ E_{NR,t}^{\left(k\right)}=E^{\left(k\right)}({\hat{\phi }}_{NR,t-1},{\hat{\beta }}_{NR,t-1})\\ F_{NR,t}^{\left(k\right)}=F^{\left(k\right)}({\hat{\phi }}_{NR,t-1},{\hat{\beta }}_{NR,t-1}) \end{array}$$

Upon receiving the $S_{1}^{\left(k\right)}$s from each node, the CC calculates the following weighted averages:


$$\begin{array}{c} {\bar{D}}_{\text{NR},\text{1}}=\sum_{k=1}^{K}w^{\left(k\right)}D_{\text{NR},\text{1}}^{\left(k\right)},\;\text{and}\;{\bar{V}}_{\text{NR},\text{1}}=\sum_{k=1}^{K}w^{\left(k\right)}V_{\text{NR},\text{1}}^{\left(k\right)},\\ {\bar{E}}_{\text{NR},\text{1}}=\sum_{k=1}^{K}w^{\left(k\right)}E_{\text{NR},\text{1}}^{\left(k\right)},\;\text{and}\;{\bar{F}}_{\text{NR},\text{1}}=\sum_{k=1}^{K}w^{\left(k\right)}F_{\text{NR},\text{1}}^{\left(k\right)}. \end{array}$$

This enables the CC to execute Newton-Raphson updates from ${\hat{\beta }}_{\text{NR},\text{0}}$ and ${\hat{\phi }}_{\text{NR},\text{0}}$, respectively:


(10) $$\begin{array}{l} {\hat{\beta }}_{NR,1}={\hat{\beta }}_{NR,0}+{\bar{V}}_{NR,1}^{-1}{\bar{D}}_{NR,1}\\ {\hat{\phi }}_{NR,1}={\hat{\phi }}_{NR,0}+{\bar{F}}_{NR,1}^{-1}{\bar{E}}_{NR,1}. \end{array}$$

It is shown in the Multimedia Appendix 2 that


$$\begin{array}{c} \sqrt{n}\left({\hat{\beta }}_{\text{NR},1}-\beta^{⋆}\right)\to N\left(0,\Sigma_{\text{NR}}\right)\\ \;\;\text{where}\;\Sigma_{\text{NR}}={\left(\sum_{k=1}^{K}w^{\left(k\right)}T_{\beta^{⋆}}^{\left(k\right)}\right)}^{-1}\left\{\phi^{⋆}\sum_{k=1}^{K}\frac{w^{(k{)}^{2}}}{p^{\left(k\right)}}T_{\beta^{⋆}}^{\left(k\right)}\right\}{\left(\sum_{k=1}^{K}w^{\left(k\right)}T_{\beta^{⋆}}^{\left(k\right)}\right)}^{-1}. \end{array}$$

As $T_{\beta^{⋆}}^{\left(k\right)}$ is consistently estimated using $V_{\text{NR},\text{1}}^{\left(k\right)}$ and $\phi^{⋆}$ by ${\hat{\phi }}_{\text{NR},\text{1}}$, and as $p^{\left(k\right)}$ can be estimated using $n^{\left(k\right)}/n$, it follows that a consistent estimator for $\Sigma_{\text{NR}}$ is given by


$$\begin{array}{c} {\hat{\Sigma }}_{\text{NR}}={\left(\sum_{k=1}^{K}w^{\left(k\right)}V_{\text{NR},1}^{\left(k\right)}\right)}^{-1}\left\{{\hat{\phi }}_{\text{NR},1}\sum_{k=1}^{K}\frac{nw^{(k{)}^{2}}}{n^{\left(k\right)}}V_{\text{NR},1}^{\left(k\right)}\right\}{\left(\sum_{k=1}^{K}w^{\left(k\right)}V_{\text{NR},1}^{\left(k\right)}\right)}^{-1}\\ ={\left({\bar{V}}_{\text{NR},1}\right)}^{-1}\left\{{\hat{\phi }}_{\text{NR},1}\sum_{k=1}^{K}\frac{nw^{(k{)}^{2}}}{n^{\left(k\right)}}V_{\text{NR},1}^{\left(k\right)}\right\}{\left({\bar{V}}_{\text{NR},1}\right)}^{-1}. \end{array}$$

The method’s final estimates are then given by


$$R=\left\{{\hat{\beta }}_{\text{NR},\text{1}},{\hat{\Sigma }}_{\text{NR}}\right\}.$$

#### Multiple Distributed Newton-Raphson Updatings

The multiple distributed Newton-Raphson updatings method follows upon execution of algorithm 2 with *T*>1.

The first communication cycle follows the same procedure described previously for the single distributed Newton-Raphson updating method. It involves distributively computing a simple averaging estimator and then performing a Newton-Raphson iteration starting from this estimator. The Newton descent is calculated as described in equation 10.

Formally, the algorithm begins with each data node *k* sending the set of quantities $S_{0}^{\left(k\right)}$, as described in equation 8, to the CC. Next, the CC calculates the simple averaging estimators using formula 7 and uses them to initialize ${\hat{\beta }}_{\text{NR,Step=0}}={\hat{\beta }}_{\text{SA}}$ and ${\hat{\phi }}_{\text{NR,Step=0}}={\hat{\phi }}_{\text{SA}}$.

The following steps are then repeated for a certain number of iterations. At iteration t, starting from t=1, the CC broadcasts the values $C_{t}=\left({\hat{\beta }}_{\text{NR},\text{t-1}},{\hat{\phi }}_{\text{NR},\text{t-1}}\right)$ to the data nodes. The data nodes compute the quantities $D_{\text{NR},\text{t}}^{\left(k\right)}$, $V_{\text{NR},\text{t}}^{\left(k\right)}$, $E_{\text{NR},\text{t}}^{\left(k\right)}$, and $F_{\text{NR},\text{t}}^{\left(k\right)}$ as defined in equation 9 and send them back to the CC.

The CC then uses these quantities to perform a Newton update. Specifically, it calculates ${\hat{\beta }}_{\text{NR},\text{t}}={\hat{\beta }}_{\text{NR},\text{t-1}}+V_{\text{NR},\text{t}}^{-1}D_{\text{NR},\text{t}}$ and ${\hat{\phi }}_{\text{NR},\text{t}}={\hat{\phi }}_{\text{NR},\text{t-1}}+E_{\text{NR},\text{t}}/F_{\text{NR},\text{t}}$.

If the iterative cycle is repeated until convergence, the resulting estimates of $\beta^{⋆}$ are equivalent to the MLEs derived from pooled data. This is because, in GLMs, for MLEs, if both the pooled and distributed algorithms are initialized using the same values for ${\hat{\beta }}_{\text{NR,Step=0}}$ and ${\hat{\phi }}_{\text{NR,Step=0}}$, then, at each subsequent iteration, the distributed Newton update computed by the CC will be identical to the update obtained in a pooled setting.

For the method to yield consistent estimates, it is not necessary to initialize it using simple averaging estimators. However, using simple averaging estimators as initialization may speed up convergence, particularly in large sample sizes, as these estimators are $\sqrt{n}$-consistent.

Let ${\hat{\beta }}_{\text{NR},\text{T}}$ denote the estimator obtained at convergence. As it is (nearly) equal to the pooled MLE of $\beta^{⋆}$, we can deduce from the Multimedia Appendix 2 that


(11) $$\begin{array}{c} \sqrt{n}\left({\hat{\beta }}_{\text{NR},T}-\beta^{⋆}\right)\to N\left(0,\Sigma_{\text{NR}}\right)\\ \;\;\text{where}\;\Sigma_{\text{NR}}={\left(\sum_{k=1}^{K}w^{\left(k\right)}T_{\beta^{⋆}}^{\left(k\right)}\right)}^{-1}\left\{\phi^{⋆}\sum_{k=1}^{K}\frac{w^{(k{)}^{2}}}{p^{\left(k\right)}}T_{\beta^{⋆}}^{\left(k\right)}\right\}{\left(\sum_{k=1}^{K}w^{\left(k\right)}T_{\beta^{⋆}}^{\left(k\right)}\right)}^{-1}. \end{array}$$

Following the same reasoning used previously for the single distributed Newton-Raphson updating method, we can consistently estimate the variance-covariance matrix as


$${\hat{\Sigma }}_{\text{NR}}={\left({\bar{V}}_{\text{NR},T}\right)}^{-1}\left\{{\hat{\phi }}_{\text{NR},T}\sum_{k=1}^{K}\frac{nw^{(k{)}^{2}}}{n^{\left(k\right)}}V_{\text{NR},T}^{\left(k\right)}\right\}{\left({\bar{V}}_{\text{NR},T}\right)}^{-1}.$$

#### Distributed Estimating Equation

The distributed estimating equation method follows upon execution of algorithm 3 (Textbox 3). First, each node is responsible for computing the MLEs ${\hat{\beta }}_{\text{MLE}}^{\left(k\right)}$ and ${\hat{\phi }}_{\text{MLE}}^{\left(k\right)}$ of $\beta^{⋆}$ and $\phi^{⋆}$, respectively, using its own data. These estimators, along with the Hessian matrix $V_{\text{MLE}}^{\left(k\right)}=V^{\left(k\right)}\left({\hat{\beta }}_{\text{MLE}}^{\left(k\right)}\right)$ and $F_{\text{MLE}}^{\left(k\right)}=F^{\left(k\right)}\left({\hat{\phi }}_{\text{MLE}}^{\left(k\right)}\right)$, are then sent to the CC. The set


(12) $$\begin{array}{c} S_{0}^{\left(k\right)}=\left\{{\hat{\beta }}_{\text{MLE}}^{\left(k\right)},{\hat{\phi }}_{\text{MLE}}^{\left(k\right)},V_{\text{MLE}}^{\left(k\right)},F_{\text{MLE}}^{\left(k\right)}\right\} \end{array}$$

is transmitted to the CC. The CC calculates the weighted average of the Hessians and the $F_{\text{MLE}}^{\left(k\right)}$ values as follows:


(13) $$\begin{array}{c} {\bar{V}}_{\text{EE}}=\sum_{k=1}^{K}w^{\left(k\right)}V_{\text{MLE}}^{\left(k\right)}\;\text{and}\;{\bar{F}}_{\text{EE}}=\sum_{k=1}^{K}w^{\left(k\right)}F_{\text{MLE}}^{\left(k\right)}. \end{array}$$

The parameter estimates can then be calculated as


(14) $$\begin{array}{c} {\hat{\beta }}_{\text{EE}}={\bar{V}}_{\text{EE}}^{-1}\sum_{k=1}^{K}w^{\left(k\right)}V_{\text{MLE}}^{\left(k\right)}{\hat{\beta }}_{\text{MLE}}^{\left(k\right)}\;\;{\hat{\phi }}_{\text{EE}}={\bar{F}}_{\text{EE}}^{-1}\sum_{k=1}^{K}w^{\left(k\right)}F_{\text{MLE}}^{\left(k\right)}{\hat{\phi }}_{\text{MLE}}^{\left(k\right)}. \end{array}$$

It is shown in the Multimedia Appendix 2 that $\sqrt{n}\left({\hat{\beta }}_{\text{EE}}-\beta^{⋆}\right)$ converges in distribution to a centered normal random variable with the variance-covariance matrix given by


$$\Sigma_{\text{EE}}={\left(\sum_{k=1}^{K}w^{\left(k\right)}T_{\beta^{⋆}}^{\left(k\right)}\right)}^{-1}\left\{\phi^{⋆}\sum_{k=1}^{K}\frac{w^{(k)2}}{p^{\left(k\right)}}T_{\beta^{⋆}}^{\left(k\right)}\right\}{\left(\sum_{k=1}^{K}w^{\left(k\right)}T_{\beta^{⋆}}^{\left(k\right)}\right)}^{-1}$$

It can be consistently estimated by


$${\hat{\Sigma }}_{\text{EE}}={\left({\bar{V}}_{\text{EE}}\right)}^{-1}\left\{{\hat{\phi }}_{\text{EE}}\sum_{k=1}^{k}\frac{nw^{(k{)}^{2}}}{n^{\left(k\right)}}V_{\text{MLE}}^{\left(k\right)}\right\}{\left({\bar{V}}_{\text{EE}}\right)}^{-1}$$

Textbox 3. **Algorithm 3—distributed estimating equation inference procedure.**

Input at the coordinating center (CC) level: Weights *w*^(1)^, …, *w*^(*K*)^ attributed to each node’s contribution

Step required from each node *k* ∈ {1, …, *K*}:

Using data in $D^{\left(k\right)}$, compute the following quantities:

- MLE ${\hat{\beta }}_{\text{MLE}}^{\left(k\right)}$ of $\beta^{⋆}$ by solving $D^{\left(k\right)}\left(\beta \right)=0$;
- MLE ${\hat{\phi }}_{\text{MLE}}^{\left(k\right)}$ of $\phi^{⋆}$ by solving $E^{\left(k\right)}\left(\phi ,{\hat{\beta }}_{\text{MLE}}^{\left(k\right)}\right)=0$;
- $V_{\text{MLE}}^{\left(k\right)}=V^{\left(k\right)}\left({\hat{\beta }}_{\text{MLE}}^{\left(k\right)}\right)$ using formula (3) with $\beta ={\hat{\beta }}_{\text{MLE}}^{\left(k\right)}$;
- $F_{\text{MLE}}^{\left(k\right)}=F^{\left(k\right)}\left({\hat{\phi }}_{\text{MLE}}^{\left(k\right)}\right)$ using formula (5) with $\phi ={\hat{\phi }}_{\text{MLE}}^{\left(k\right)}$.

*Send to the CC:*$S_{0}^{\left(k\right)}=\left\{{\hat{\beta }}_{\text{MLE}}^{\left(k\right)},{\hat{\phi }}_{\text{MLE}}^{\left(k\right)},V_{\text{MLE}}^{\left(k\right)},F_{\text{MLE}}^{\left(k\right)}\right\}$.

Step required from the CC:

Using the received sets of quantities $S_{0}^{\left(1\right)},\ldots ,S_{0}^{\left(K\right)}$, calculate

- Aggregated quantities ${\bar{V}}_{\text{EE}}=\sum_{k=1}^{K}w^{\left(k\right)}V_{\text{MLE}}^{\left(k\right)}$ and ${\bar{F}}_{\text{EE}}=\sum_{k=1}^{K}w^{\left(k\right)}F_{\text{MLE}}^{\left(k\right)}$
- EE estimators ${\hat{\beta }}_{\text{EE}}={\bar{V}}_{\text{EE}}^{-1}\sum_{k=1}^{K}w^{\left(k\right)}V_{\text{MLE}}^{\left(k\right)}{\hat{\beta }}_{\text{MLE}}^{\left(k\right)}$ and ${\hat{\phi }}_{\text{EE}}={\bar{F}}_{\text{EE}}^{-1}\sum_{k=1}^{K}w^{\left(k\right)}F_{\text{MLE}}^{\left(k\right)}{\hat{\phi }}_{\text{MLE}}^{\left(k\right)}$; the variance-covariance matrix ${\hat{\Sigma }}_{\text{EE}}={\left({\bar{V}}_{\text{EE}}\right)}^{-1}\left\{{\hat{\phi }}_{\text{EE}}\sum_{k=1}^{K}\frac{nw^{(k)2}}{n^{\left(k\right)}}V_{\text{MLE}}^{\left(k\right)}\right\}{\left({\bar{V}}_{\text{EE}}\right)}^{-1}$.

Output from the CC:

Parameter estimates and CIs $R=\left\{{\hat{\beta }}_{\text{SA}},{\hat{\Sigma }}_{\text{EE}}\right\}$

#### Distributed Estimation Using a Single Gradient-Enhanced Log-Likelihood

##### Overview

This method operates through algorithm 4 (Textbox 4). First, the necessary information is collected by the CC to compute the initial estimates of ***β*** and ϕ, denoted as ${\hat{\beta }}_{\text{SGE},\text{0}}$ and ${\hat{\phi }}_{\text{SGE},\text{0}}$. In what follows, we assume that these estimates are obtained using the simple averaging estimators calculated through algorithm 1.

Textbox 4. **Algorithm 4—inference procedure based on the distributed estimation using a single gradient-enhanced log-likelihood method.**

Input at the coordinating center (CC) level: Weights *w*^(1)^, …, *w*^(*K*)^ attributed to each node’s contribution

Step required from each node *k* ∈ {1, …, *K*}:

Using data in $D^{\left(k\right)}$, compute

- ${\hat{\beta }}_{\text{MLE}}^{\left(k\right)}$ by solving $D^{\left(k\right)}\left(\beta \right)=0$;
- ${\hat{\phi }}_{\text{MLE}}^{\left(k\right)}$ by solving $E^{\left(k\right)}\left(\phi ,{\hat{\beta }}_{\text{MLE}}^{\left(k\right)}\right)=0$

*Send to the CC:*$S_{0}^{\left(k\right)}=\left\{{\hat{\beta }}_{\text{MLE}}^{\left(k\right)},{\hat{\phi }}_{\text{MLE}}^{\left(k\right)}\right\}$.

Step required from the CC:

Using the received sets of quantities $S_{0}^{\left(1\right)},\ldots ,S_{0}^{\left(K\right)}$, calculate

- simple averaging estimators ${\hat{\beta }}_{\text{SA}}=\sum_{k=1}^{K}w^{\left(k\right)}{\hat{\beta }}_{\text{MLE}}^{\left(k\right)}$ and ${\hat{\phi }}_{\text{SA}}=\sum_{k=1}^{K}w^{\left(k\right)}{\hat{\phi }}_{\text{MLE}}^{\left(k\right)}$
- initialize ${\hat{\beta }}_{\text{SGE},\text{0}}={\hat{\beta }}_{\text{SA}}$ and ${\hat{\phi }}_{\text{SGE},\text{0}}={\hat{\phi }}_{\text{SA}}$.

*Broadcast to nodes:*$C_{1,1}=\left\{{\hat{\beta }}_{\text{SGE},\text{0}},{\hat{\phi }}_{\text{SGE},\text{0}}\right\}$.

Step required from each node *k* ∈ {2, …, *K*}:

Using data in $D^{\left(k\right)}$, compute the following quantities:

- $D_{\text{SGE},\text{1}}^{\left(k\right)}=D^{\left(k\right)}\left({\hat{\beta }}_{\text{SGE},\text{0}}\right)$ using formula (2) with $\beta ={\hat{\beta }}_{\text{SGE},\text{0}}$;
- $V_{\text{SGE},\text{1}}^{\left(k\right)}=V^{\left(k\right)}\left({\hat{\beta }}_{\text{SGE},\text{0}}\right)$ using formula (3) with $\beta ={\hat{\beta }}_{\text{SGE},\text{0}}$;
- $E_{\text{SGE},\text{1}}^{\left(k\right)}=E^{\left(k\right)}\left({\hat{\phi }}_{\text{SGE},\text{0}},{\hat{\beta }}_{\text{SGE},\text{0}}\right)$ using formula (4) with $\left(\phi ,\beta \right)=\left({\hat{\phi }}_{\text{SGE},\text{0}},{\hat{\beta }}_{\text{SGE},\text{0}}\right)$

*Send to the CC:* $S_{1,1}^{\left(k\right)}=\left\{D_{\text{SGE},\text{1}}^{\left(k\right)},V_{\text{SGE},\text{1}}^{\left(k\right)},E_{\text{SGE},\text{1}}^{\left(k\right)}\right\}$

Step required from the CC:

Using the received sets of quantities $S_{1,1}^{\left(2\right)},\ldots ,S_{1,1}^{\left(K\right)}$, calculate

-
$D_{\text{SGE},\text{1}}=\sum_{k=2}^{K}w^{\left(k\right)}D_{\text{SGE},\text{1}}^{\left(k\right)}$

-
$E_{\text{SGE},\text{1}}=\sum_{k=2}^{K}w^{\left(k\right)}E_{\text{SGE},\text{1}}^{\left(k\right)}$

*Broadcast to node k*=1: *C*_2,1_ = {**D**_SGE,1_ , ***E***_SGE,1_}

Step required from node *k=*1:

Using data in $D^{\left(1\right)}$, calculate

- $D_{\text{SGE},\text{1}}^{\left(1\right)}=D^{\left(1\right)}\left({\hat{\beta }}_{\text{SGE},\text{0}}\right)$ using formula (2) with $\beta ={\hat{\beta }}_{\text{SGE},\text{0}}$;
- $V_{\text{SGE},\text{1}}^{\left(1\right)}=V^{\left(1\right)}\left({\hat{\beta }}_{\text{SGE},\text{0}}\right)$ using formula (3) with $\beta ={\hat{\beta }}_{\text{SGE},\text{0}}$;
- $E_{\text{SGE},\text{1}}^{\left(1\right)}=E^{\left(1\right)}\left({\hat{\phi }}_{\text{SGE},\text{0}},{\hat{\beta }}_{\text{SGE},\text{0}}\right)$ using formula (4) with $\left(\phi ,\beta \right)=\left({\hat{\phi }}_{\text{SGE},\text{0}},{\hat{\beta }}_{\text{SGE},\text{0}}\right)$
-
${\bar{D}}_{\text{SGE},\text{1}}=D_{\text{SGE},\text{1}}+w^{\left(1\right)}D_{\text{SGE},\text{1}}^{\left(1\right)}$

-
${\bar{E}}_{\text{SGE},\text{1}}=E_{\text{SGE},\text{1}}+w^{\left(1\right)}E_{\text{SGE},\text{1}}^{\left(1\right)}$

*Send to the CC:* $S_{2,1}^{\left(1\right)}=\left\{{\hat{\beta }}_{\text{SGE},\text{1}},{\hat{\phi }}_{\text{SGE},\text{1}},V_{\text{SGE},\text{1}}^{\left(1\right)}\right\}$

Step required from the CC:

Compute

- ${\hat{A}}_{\text{SGE},\text{1}}^{\left(k\right)}=w^{\left(k\right)}I_{p+1}+\left\{V_{\text{SGE},\text{1}}^{\left(1\right)}-\left(\sum_{k^{\prime }=1}^{K}w^{\left(k^{\prime }\right)}V_{\text{SGE},\text{1}}^{\left(k^{\prime }\right)}\right)\right\}{\left(V_{\text{SGE},\text{1}}^{\left(k\right)}\right)}^{-1}$ for *k* ∈ {1, …, *K*}
-
${\hat{\Sigma }}_{\text{SGE},\text{1}}={\hat{\phi }}_{\text{SGE},\text{1}}{\left(V_{\text{SGE},\text{1}}^{\left(1\right)}\right)}^{-1}\left\{\sum_{k=1}^{K}\frac{n}{n^{\left(k\right)}}{\left({\hat{A}}_{\text{SGE},\text{1}}^{\left(k\right)}\right)}^{⊤}V_{\text{SGE},\text{1}}^{\left(k\right)}{\hat{A}}_{\text{SGE},\text{1}}^{\left(k\right)}\right\}{\left(V_{\text{SGE},\text{1}}^{\left(1\right)}\right)}^{-1}$

Output from the CC:

Parameter estimates $R=\left\{{\hat{\beta }}_{\text{SGE},\text{1}},{\hat{\Sigma }}_{\text{SGE},\text{1}}\right\}$

Subsequently, the CC broadcasts $C_{1,1}=\left\{{\hat{\beta }}_{\text{SGE},\text{0}},{\hat{\phi }}_{\text{SGE},\text{0}}\right\}$ to node *k* ∈ {1, …, *K*}. Each node is then requested to compute and transmit back the following quantities:


$$\begin{array}{c} S_{1,1}^{\left(k\right)}=\left\{D_{\text{SGE},\text{1}}^{\left(k\right)},V_{\text{SGE},\text{1}}^{\left(k\right)},E_{\text{SGE},\text{1}}^{\left(k\right)}\right\}, \end{array}$$

with $D_{\text{SGE},\text{1}}^{\left(k\right)}=D^{\left(k\right)}\left({\hat{\beta }}_{\text{SGE},\text{0}}\right),V_{\text{SGE},\text{1}}^{\left(k\right)}=V^{\left(k\right)}\left({\hat{\beta }}_{\text{SGE},\text{0}}\right),and\;E_{\text{SGE},\text{1}}^{\left(k\right)}=E^{\left(k\right)}\left({\hat{\phi }}_{\text{SGE},\text{0}},{\hat{\beta }}_{\text{SGE},\text{0}}\right)$

The CC aggregates the $D^{\left(k\right)}$s and the $E^{\left(k\right)}$s using averaging by calculating


$$\begin{array}{c} D_{\text{SGE},\text{1}}=\sum_{k=2}^{K}w^{\left(k\right)}D_{\text{SGE},\text{1}}^{\left(k\right)}\;\text{and}\;E_{\text{SGE},\text{1}}=\sum_{k=2}^{K}w^{\left(k\right)}E_{\text{SGE},\text{1}}^{\left(k\right)}\;. \end{array}$$

The $V^{\left(k\right)}$s are momentarily stored and will be used later to compute the estimator for the asymptotic variance-covariance matrix of the final estimator of $\beta^{⋆}$. The quantities


$$C_{2,1}=\left\{D_{\text{SGE},\text{1}},E_{\text{SGE},\text{1}}\right\}$$

are then sent to node k=1. Node k=1 computes the global average of the $D^{\left(k\right)}$s by adding its own counterpart; that is, it first computes


$$\begin{array}{c} {\bar{D}}_{\text{SGE},\text{1}}=D_{\text{SGE},\text{1}}+w^{\left(1\right)}D_{\text{SGE},\text{1}}^{\left(1\right)},\;\text{and}\;{\bar{E}}_{\text{SGE},\text{1}}=E_{\text{SGE},\text{1}}+w^{\left(1\right)}E_{\text{SGE},\text{1}}^{\left(1\right)}, \end{array}$$

and then solves the surrogate likelihood function. Formally, it finds successively the values ${\hat{\beta }}_{\text{SGE},\text{1}}^{\left(1\right)}$ and ${\hat{\phi }}_{\text{SGE},\text{1}}^{\left(1\right)}$ that solve


$$\begin{array}{c} D^{\left(1\right)}\left(\beta \right)+{\bar{D}}_{\text{SGE},\text{1}}-D_{\text{SGE},\text{1}}^{\left(1\right)}=0\\ E^{\left(1\right)}\left(\phi ,{\hat{\beta }}_{\text{SGE},\text{1}}\right)+{\bar{E}}_{\text{SGE},\text{1}}-E_{\text{SGE},\text{1}}^{\left(1\right)}=0\;. \end{array}$$

The results are sent back to the CC, along with $V_{\text{SGE},\text{1}}^{\left(1\right)}$, yielding


$$S_{2,1}^{\left(1\right)}=\left\{{\hat{\beta }}_{\text{SGE},\text{1}},{\hat{\phi }}_{\text{SGE},\text{1}},V_{\text{SGE},\text{1}}^{\left(1\right)}\right\}.$$

If simple averaging estimators for ${\hat{\beta }}_{\text{SGE},\text{0}}$ and ${\hat{\phi }}_{\text{SGE},\text{0}}$ are chosen, then $\sqrt{n}\left({\hat{\beta }}_{\text{SGE},\text{1}}-\beta^{⋆}\right)$ converges in distribution to a mean-zero multivariate normal random variable with a variance-covariance matrix given by


$$\begin{array}{cc} \Sigma_{\text{SGE},\text{1}}= & \phi^{⋆}{\left(T_{\beta^{⋆}}^{\left(1\right)}\right)}^{-1}\left\{\sum_{k=1}^{K}\frac{w^{(k{)}^{2}}}{p^{\left(k\right)}}{\left(A_{\beta^{⋆}}^{\left(k\right)}\right)}^{⊤}T_{\beta^{⋆}}^{\left(k\right)}A_{\beta^{⋆}}^{\left(k\right)}\right\}{\left(T_{\beta^{⋆}}^{\left(1\right)}\right)}^{-1}\\ \;\;\text{where}\;\;A_{\beta^{⋆}}^{\left(k\right)}=\sqrt{p^{\left(k\right)}}I_{p+1}+\left(T_{\beta^{⋆}}^{\left(1\right)}-T_{\beta^{⋆}}\right){\left(T_{\beta^{⋆}}^{\left(k\right)}\right)}^{-1}, \end{array}$$

with $I_{p+1}$, the *p*+1 square identity matrix, and $T_{\beta^{⋆}}=\sum_{k=1}^{K}w^{\left(k\right)}T_{\beta^{⋆}}^{\left(k\right)}$. The Multimedia Appendix 2 contains the proofs. The latter can be consistently estimated by


$${\hat{\Sigma }}_{\text{SGE},1}={\hat{\phi }}_{\text{SGE},1}{\left(V_{\text{SGE},1}^{\left(1\right)}\right)}^{-1}\left\{\sum_{k=1}^{K}\frac{w^{(k)2}n}{n^{\left(k\right)}}{\left({\hat{A}}_{\text{SGE},1}^{\left(k\right)}\right)}^{⊤}V_{\text{SGE},1}^{\left(k\right)}{\hat{A}}_{\text{SGE},1}^{\left(k\right)}\right\}{\left(V_{\text{SGE},1}^{\left(1\right)}\right)}^{-1}$$

where


$${\hat{A}}_{\text{SGE},\text{1}}^{\left(k\right)}=\sqrt{\frac{n^{\left(k\right)}}{n}}I_{p+1}+\left\{V_{\text{SGE},\text{1}}^{\left(1\right)}-{\hat{T}}_{\beta^{⋆}}\right\}{\left(V_{\text{SGE},\text{1}}^{\left(k\right)}\right)}^{-1}$$

With ${\hat{T}}_{\beta^{⋆}}=\sum_{k=1}^{K}w^{\left(k\right)}V_{\text{SGE},\text{1}}^{\left(k\right)}$.

##### Remark

In the study by Jordan et al [13], where the method described previously was originally proposed, the authors discuss a version in which the latter process is repeated multiple times. Their version assumes that the data are uniformly and randomly split across nodes. Under this assumption, the resulting estimator of $\beta^{⋆}$ is asymptotically equivalent to the pooled estimator regardless of the number of iterations executed. This equivalence occurs because, when the predictors’ distribution is the same across nodes and the node sample sizes are equal, then $T_{\beta^{⋆}}^{\left(k\right)}\equiv T_{\beta^{⋆}}$ and $p^{\left(k\right)}\equiv 1/K$. By choosing $w^{\left(k\right)}=1/K$, it follows that $A_{\beta^{⋆}}^{\left(k\right)}=I_{p+1}$, resulting in the following expression for $\Sigma_{\text{SGE},\text{1}}$: $\begin{array}{cc} \Sigma_{\text{SGE},\text{1}}= & \phi^{⋆}{\left(T_{\beta^{⋆}}\right)}^{-1}. \end{array}$ The aforementioned variance-covariance matrix is also the same as that of the simple averaging estimator in the setting of equal sample sizes and even predictor distributions. Consequently, at each iteration, the probability distribution of the resulting estimator remains unchanged. However, in a more general setting in which predictor distributions and sample sizes vary across nodes, these cancellations no longer occur. Therefore, in this case, the probability distribution of the obtained estimator changes after each iteration, and tracking these changes falls beyond the scope of objective 3 (see the Multimedia Appendix 2). Hence, this presentation focused on the case in which only 1 iteration is executed.

### Distributed Estimation Using Multiple Gradient-Enhanced Log-Likelihood

This method operates through algorithm 5 (Textbox 5). First, the CC collects the necessary information to compute the initial estimates, denoted as ${\hat{\beta }}_{\text{MGE},\text{0}}$ and ${\hat{\phi }}_{\text{MGE},\text{0}}$. In this case, we assume that these estimates are obtained using the simple averaging estimators calculated through algorithm 1.

Textbox 5. **Algorithm 5—inference procedure based on the distributed estimation using a multiple gradient-enhanced log-likelihood method.**

Input at the coordinating center (CC) level: Weight *w*^(1)^, …, *w*^(*K*)^ attributed to each node’s contribution

Step required from each node *k* ∈ {1, …, *K*}:

Using data in $D^{\left(k\right)}$, compute

- ${\hat{\beta }}_{\text{MLE}}^{\left(k\right)}$ by solving $D^{\left(k\right)}\left(\beta \right)=0$;
- ${\hat{\phi }}_{\text{MLE}}^{\left(k\right)}$ by solving $E^{\left(k\right)}\left(\phi ,{\hat{\beta }}_{\text{MLE}}^{\left(k\right)}\right)=0$

*Send to the CC:*$S_{0}^{\left(k\right)}=\left\{{\hat{\beta }}_{\text{MLE}}^{\left(k\right)},{\hat{\phi }}_{\text{MLE}}^{\left(k\right)}\right\}$.

Step required from the CC:

Using the received sets of quantities $S_{0}^{\left(1\right)},\ldots ,S_{0}^{\left(K\right)}$, calculate

- simple averaging estimators ${\hat{\beta }}_{\text{SA}}=\sum_{k=1}^{K}w^{\left(k\right)}{\hat{\beta }}_{\text{MLE}}^{\left(k\right)}$ and ${\hat{\phi }}_{\text{SA}}=\sum_{k=1}^{K}w^{\left(k\right)}{\hat{\phi }}_{\text{MLE}}^{\left(k\right)}$
- initialize ${\hat{\beta }}_{\text{MGE},\text{0}}={\hat{\beta }}_{\text{SA}}$ and ${\hat{\phi }}_{\text{MGE},\text{0}}={\hat{\phi }}_{\text{SA}}$.

*Broadcast to nodes:*$C_{1,1}=\left\{{\hat{\beta }}_{\text{MGE},\text{0}},{\hat{\phi }}_{\text{MGE},\text{0}}\right\}$.

Step required from each node *k* ∈ {1, …, *K*}:

Using data in $D^{\left(k\right)}$, calculate

- $D_{\text{MGE},\text{1}}^{\left(k\right)}=D^{\left(k\right)}\left({\hat{\beta }}_{\text{MGE},\text{0}}\right)$ using formula (2) with $\beta ={\hat{\beta }}_{\text{MGE},\text{0}}$;
- $V_{\text{MGE},\text{1}}^{\left(k\right)}=V^{\left(k\right)}\left({\hat{\beta }}_{\text{MGE},\text{0}}\right)$ using formula (3) with $\beta ={\hat{\beta }}_{\text{MGE},\text{0}}$;
- $E_{\text{MGE},\text{1}}^{\left(k\right)}=E^{\left(k\right)}\left({\hat{\phi }}_{\text{MGE},\text{0}},{\hat{\beta }}_{\text{MGE},\text{0}}\right)$ using formula (4) with $\left(\phi ,\beta \right)=\left({\hat{\phi }}_{\text{MGE},\text{0}},{\hat{\beta }}_{\text{MGE},\text{0}}\right)$.

*Send to the CC:* $S_{1,1}^{\left(k\right)}=\left\{D_{\text{MGE},\text{1}}^{\left(k\right)},V_{\text{MGE},\text{1}}^{\left(k\right)},E_{\text{MGE},\text{1}}^{\left(k\right)}\right\}$

Step required from the CC:

Using the received sets of quantities $S_{1,1}^{\left(1\right)},\ldots ,S_{1,1}^{\left(K\right)}$, calculate

-
${\bar{D}}_{\text{MGE},\text{1}}=\sum_{k=1}^{K}w^{\left(k\right)}D_{\text{MGE},\text{1}}^{\left(k\right)}$

-
${\bar{E}}_{\text{MGE},\text{1}}=\sum_{k=1}^{K}w^{\left(k\right)}E_{\text{MGE},\text{1}}^{\left(k\right)}$

*Broadcast to nodes:*$C_{2,1}=\left\{{\bar{D}}_{\text{MGE},\text{1}},{\bar{E}}_{\text{MGE},\text{1}}\right\}$.

Step required from each node *k* ∈ {1, …, *K*}:

Using data in $D^{\left(k\right)}$, calculate

- ${\hat{\beta }}_{\text{MGE},\text{1}}^{\left(k\right)}$ that solves $D^{\left(k\right)}\left(\beta \right)+{\bar{D}}_{\text{MGE},\text{1}}-D_{\text{MGE},\text{1}}^{\left(k\right)}=0$
- ${\hat{\phi }}_{\text{MGE},\text{1}}^{\left(k\right)}$ that solves $E^{\left(k\right)}\left(\phi ,{\hat{\beta }}_{\text{MGE},\text{1}}\right)+{\bar{E}}_{\text{MGE},\text{1}}-E_{\text{MGE},\text{1}}^{\left(k\right)}=0$

*Send to the CC:* $S_{2,1}^{\left(k\right)}=\left\{{\hat{\beta }}_{\text{MGE},\text{1}}^{\left(k\right)},{\hat{\phi }}_{\text{MGE},\text{1}}^{\left(k\right)}\right\}$

Step required from the CC:

Compute

-
${\hat{\beta }}_{\text{MGE},\text{1}}=\sum_{k=1}^{K}w^{\left(k\right)}{\hat{\beta }}_{\text{MGE},\text{1}}^{\left(k\right)}$

-
${\hat{\phi }}_{\text{MGE},\text{1}}=\sum_{k=1}^{K}w^{\left(k\right)}{\hat{\phi }}_{\text{MGE},\text{1}}^{\left(k\right)}$

-
${\hat{T}}_{\beta^{⋆}}=\sum_{k=1}^{K}w^{\left(k\right)}V_{\text{MGE},\text{1}}^{\left(k\right)}$

-
${\hat{U}}_{\beta^{⋆}}=\sum_{k=1}^{K}w^{\left(k\right)}{\left(V_{\text{MGE},\text{1}}^{\left(k\right)}\right)}^{-1}$

-
${\hat{\Sigma }}_{\text{MGE},1}={\hat{\phi }}_{\text{MGE},1}\sum_{k=1}^{K}\frac{nw^{(k)2}}{n^{\left(k\right)}}\left[{\hat{U}}_{\beta^{⋆}}V_{\text{MGE},1}^{\left(k\right)}+\left(I_{p+1}-{\hat{U}}_{\beta^{⋆}}{\hat{T}}_{\beta^{⋆}}\right)\right]\;\;\times \;\left[{\hat{U}}_{\beta^{⋆}}+\left(I_{p+1}-{\hat{U}}_{\beta^{⋆}}{\hat{T}}_{\beta^{⋆}}\right){\left(V_{\text{MGE},1}^{\left(k\right)}\right)}^{-1}\right]$

Output from the CC:

Parameter estimates $R=\left\{{\hat{\beta }}_{\text{MGE},\text{1}},{\hat{\Sigma }}_{\text{MGE},\text{1}}\right\}$

Subsequently, the CC broadcasts $C_{1,1}=\left\{{\hat{\beta }}_{\text{MGE},\text{0}},{\hat{\phi }}_{\text{MGE},\text{0}}\right\}$ to each node, which is then requested to compute and transmit back the following quantities:


$$\begin{array}{c} S_{1,1}^{\left(k\right)}=\left\{D_{\text{MGE},\text{1}}^{\left(k\right)},V_{\text{MGE},\text{1}}^{\left(k\right)},E_{\text{MGE},\text{1}}^{\left(k\right)}\right\}. \end{array}$$

Here $D_{\text{MGE},\text{1}}^{\left(k\right)}=D^{\left(k\right)}\left({\hat{\beta }}_{\text{MGE},\text{0}}\right),V_{\text{MGE},\text{1}}^{\left(k\right)}=V^{\left(k\right)}\left({\hat{\beta }}_{\text{MGE},\text{0}}\right),and\;E_{\text{MGE},\text{1}}^{\left(k\right)}=E^{\left(k\right)}\left({\hat{\phi }}_{\text{MGE},\text{0}},{\hat{\beta }}_{\text{MGE},\text{0}}\right)$.

The CC aggregates the $D^{\left(k\right)}$s and the $E^{\left(k\right)}$s using averaging by calculating $\begin{array}{c} {\bar{D}}_{\text{MGE},\text{1}}=\sum_{k=1}^{K}w^{\left(k\right)}D_{\text{MGE},\text{1}}^{\left(k\right)}\;\text{and}\;{\bar{E}}_{\text{MGE},\text{1}}=\sum_{k=1}^{K}w^{\left(k\right)}E_{\text{MGE},\text{1}}^{\left(k\right)}. \end{array}$

The CC then broadcasts $C_{2,1}=\left\{{\bar{D}}_{\text{MGE},\text{1}},{\bar{E}}_{\text{MGE},\text{1}}\right\}$ to each node, which is then tasked with solving the surrogate likelihood function. Formally, they find successively the value ${\hat{\beta }}_{\text{MGE},\text{1}}^{\left(k\right)}$ and ${\hat{\phi }}_{\text{MGE},\text{1}}^{\left(k\right)}$ that solves

.$$\begin{array}{c} D^{\left(k\right)}\left(\beta \right)+{\bar{D}}_{\text{MGE},\text{1}}-D_{\text{MGE},\text{1}}^{\left(k\right)}=0\\ E^{\left(k\right)}\left(\phi ,{\hat{\beta }}_{\text{MGE},\text{1}}^{\left(k\right)}\right)+{\bar{E}}_{\text{MGE},\text{1}}-E_{\text{MGE},\text{1}}^{\left(k\right)}=0 \end{array}$$

Each node then transmits their set of local surrogate likelihood estimators to the CC:


$$S_{2,1}^{\left(k\right)}=\left\{{\hat{\beta }}_{\text{MGE},\text{1}}^{\left(k\right)},{\hat{\phi }}_{\text{MGE},\text{1}}^{\left(k\right)}\right\}.$$

Using the received sets of quantities $S_{2,1}^{\left(1\right)},\ldots ,S_{2,1}^{\left(K\right)}$, the CC aggregates them through averaging using the following formulas:


$${\hat{\beta }}_{\text{MGE},\text{1}}=\sum_{k=1}^{K}w^{\left(k\right)}{\hat{\beta }}_{\text{MGE},\text{1}}^{\left(k\right)}\;\text{and}\;{\hat{\phi }}_{\text{MGE},\text{1}}=\sum_{k=1}^{K}w^{\left(k\right)}{\hat{\phi }}_{\text{MGE},\text{1}}^{\left(k\right)}.$$

It is shown in the Multimedia Appendix 2 that $\sqrt{n}\left({\hat{\beta }}_{\text{MGE},\text{1}}-\beta^{⋆}\right)$ converges in distribution to a multivariate normal random variable with mean 0 and a variance-covariance matrix given by


$$\begin{array}{l} \Sigma_{\text{MGE},1}=\phi^{⋆}\sum_{k=1}^{K}\frac{w^{(k)2}}{p^{\left(k\right)}}\left[\sqrt{p^{\left(k\right)}}U_{\beta^{⋆}}T_{\beta^{⋆}}^{\left(k\right)}+\left(I_{p+1}-U_{\beta^{⋆}}T_{\beta^{⋆}}\right)\right]\\ \;\times \left[\sqrt{p^{\left(k\right)}}U_{\beta^{⋆}}+\left(I_{p+1}-U_{\beta^{⋆}}T_{\beta^{⋆}}\right){\left(T_{\beta^{⋆}}^{\left(k\right)}\right)}^{-1}\right] \end{array}$$

where $U_{\beta^{⋆}}=\sum_{k=1}^{K}w^{\left(k\right)}{\left(T_{\beta^{⋆}}^{\left(k\right)}\right)}^{-1}$. The latter can be consistently estimated using


$$\begin{array}{l} {\hat{\Sigma }}_{\text{MGE},1}={\hat{\phi }}_{\text{MGE},1}\sum_{k=1}^{K}\frac{nw^{(k)2}}{n^{\left(k\right)}}\left[\sqrt{\frac{n^{\left(k\right)}}{n}}{\hat{U}}_{\beta^{⋆}}V_{\text{MGE},1}^{\left(k\right)}+\left(I_{p+1}-{\hat{U}}_{\beta^{⋆}}{\hat{T}}_{\beta^{⋆}}\right)\right]\\ \;\;\;\times \left[\sqrt{\frac{n^{\left(k\right)}}{n}}{\hat{U}}_{\beta^{⋆}}+\left(I_{p+1}-{\hat{U}}_{\beta^{⋆}}{\hat{T}}_{\beta^{⋆}}\right){\left(V_{\text{MGE},1}^{\left(k\right)}\right)}^{-1}\right] \end{array}$$

where ${\hat{T}}_{\beta^{⋆}}=\sum_{k=1}^{K}w^{\left(k\right)}V_{\text{MGE},\text{1}}^{\left(k\right)}\;and\;{\hat{U}}_{\beta^{⋆}}=\sum_{k=1}^{K}w^{\left(k\right)}{\left(V_{\text{MGE},\text{1}}^{\left(k\right)}\right)}^{-1}$

### Summary of Quantities Exchanged in the Adapted Methods

Table 2 presents a summary of the quantities exchanged between the nodes and the CC in both directions. Table 2 demonstrates that the quantities involved in exchanges from the nodes to the CC consist of parameter estimates, gradients ($D^{\left(k\right)}$ vectors), Hessians ($V^{\left(k\right)}$ matrices), and real numbers ($E^{\left(k\right)}$ and $F^{\left(k\right)}$). On the other hand, the quantities shared from the CC to the nodes primarily consist of parameter estimates. Notably, methods 5 and 6 differentiate themselves by requiring the sharing of aggregated gradient vectors and Hessian matrices as well.

### Comparison of Adapted Methods

Table 3 compares the main adapted HPSA methods regarding the quantities shared between the CC and the nodes and the operational complexity of the procedures. Methods 1 and 4 require only 1 communication from the data nodes to the CC and no communication back from the CC to the nodes. These so-called one-shot methods have the lowest operational complexity. Method 4 requires the additional quantity $F_{\text{MLE}}^{\left(k\right)}$ to be transmitted from each node to the CC.

**Table 3.** Comparison of adapted methods.

Method number	Method	Information shared		Number of communications		Workflow
		From nodes to CC^a^	From CC to nodes	From nodes to CC	From CC to nodes	
1	Simple averaging	Local parameter estimates; Hessian matrix of log-likelihood (with respect to ***β*** only)	None	1	0	I in Figure 2
2	Single distributed Newton-Raphson updating	Local parameter estimates; gradient and Hessian of log-likelihood	Simple averaging aggregated estimates of parameters	2	1	II in Figure 3 with *T*=1
3	Multiple distributed Newton-Raphson updatings (with *T* Newton-Raphson updatings)	Local parameter estimates; *T* × gradient and Hessian of log-likelihood	Simple averaging aggregated estimates of parameters; (*T-1)* × Newton-updated parameter estimates	*T*+1	*T*	II in Figure 3 with T>1
4	Distributed estimating equations	Local parameter estimates; Hessian of log-likelihood	None	1	0	I in Figure 2
5	Distributed single gradient-enhanced log-likelihood	From all nodes: local parameter estimates and Hessian of log-likelihood (with respect to ***β*** only); from nodes 2 to *K*: gradient of log-likelihood; from node 1 only: gradient-enhanced parameter estimates	To all nodes: simple averaging aggregated estimates of parameters; to node 1 only: average of local gradients and Hessians	All nodes: 2	Nodes 2 to *K*: 1; node 1: 2	III in Figure 4 with *T*=1
6	Distributed multiple gradient-enhanced log-likelihood	Local parameter estimates; gradient of log-likelihood; Hessian of log-likelihood (with respect to ***β*** only); gradient-enhanced parameter estimates	Simple averaging aggregated estimates of parameters; average of local gradients and Hessians	3	2	IV in Figure 5 with *T*=1

a CC: coordinating center.

Methods 2 and 3 perform Newton-Raphson updating using some initial estimator as a basis, usually the simple averaging estimator. While method 2 requires this initial estimator to be $\sqrt{n}$-consistent, if *T* is large enough, any initial value will work for method 3 (although convergence may be slower). Both methods require $D^{\left(k\right)},V^{\left(k\right)},E^{\left(k\right)}$, and $F^{\left(k\right)}$ to be evaluated and sent to the CC *T* times, with *T*=1 for method 2. Compared to method 1, method 2 requires the additional quantities $D^{\left(k\right)}$, $E^{\left(k\right)}$, and $F^{\left(k\right)}$, and method 3 further requires these quantities to be evaluated and communicated multiple times.

Method 5 relies on an approximation of the log-likelihood function. It requires an initial estimator, usually the simple averaging estimator. This approach treats node 1 differently, making it solve the surrogate log-likelihood using aggregates from the other nodes and its own data. The CC sends the initial estimator to each node and then requires them to evaluate $D^{\left(k\right)},V^{\left(k\right)}$, and $E^{\left(k\right)}$ and send the result back to the CC once. It averages the results and then communicates them to node 1, which solves the surrogate log-likelihood and sends its results back to the CC.

Method 6 applies method 5 to every node, making each node solve the surrogate log-likelihood function using its own data before averaging the resulting local estimators.

## Discussion

### Summary of Findings

The first objective of this study (objective 1) aimed to identify and map the methodological approaches used and developed in the literature regarding HPSA. To achieve this, we conducted a scoping review, which included 41 articles following our protocol. These articles were categorized based on the types of models and communication schemes involved, as presented in Table 1. The analysis revealed that most methods included in this scoping review focused on methodological settings associated with massive data. The communication schemes of these methods were demonstrated through workflows I, II, III, and IV.

The second objective of this study (objective 2) aimed to describe the approaches that can be used for basic GLM regression analyses and identify the distributional assumptions they require. To accomplish this, we identified 6 approaches and classified them within workflows I to IV, enabling their comparison in terms of operational communication protocols within a unified framework. However, a limitation of these methods is that they assume identical node sample sizes and node covariate distributions. This assumption reduces their suitability in settings commonly encountered in health analytics, where data-collecting nodes are prone to generating different covariate distributions.

The third objective of this study (objective 3) was to present methods that relaxed these assumptions by adapting the approaches identified in objective 2 to the unequal sample sizes and nonidentical covariate sample distribution setting. In addition, we compared these methods in terms of the information shared and operational complexity. This involved adapting the quantities and estimators described in the original articles and deriving new asymptotic results with relaxed assumptions. We defined a unified framework to describe inference procedures using these methods. This unified framework encompasses common hypotheses and notation and allows for both estimation and the construction of CIs, providing detailed steps for both the data nodes and the CC.

### Challenges and Opportunities

Work pertaining to objective 1 illustrated why it is so challenging for researchers and data custodians alike to find information regarding HPSA. While the HPSA literature is very recent (all the included articles were published in 2010 or later), the literature is nonhomogeneous, and it has not come to a consensus on nomenclature. No universal terminology exists, and different terms are used in the different fields developing and applying HPSA methods. Many specific methods introduced in applied contexts are special cases of more general methods that may or may not be cited. These characteristics make finding useful and efficient keywords arduous. This required adapting our search strategy.

This difficulty is compounded by the fact that statistical inference is not the main focus of most of the HPSA literature. Most published work falls within the prediction, learning, and optimization contexts. As a result, method assumptions are rarely discussed. This can be a problem when adapting these methods for inference. Furthermore, the methodological setting is often assumed to be in the massive data context, in which data are randomly distributed between nodes. This allows the authors to make strong assumptions on node sample sizes and covariate distributions that may be unrealistic in the confidential data context using multiple data sources. These methods cannot be used directly for inference using confidential health data. While some work remains to be done when the structure of association between the covariate and the outcome is heterogeneous between nodes, we adapted widely used methods for when the distribution of covariates and sample sizes between nodes is not identical.

Table 1 illustrates how most HPSA methods are focused on parametric models. Some work has also been done for semiparametric and nonparametric regression, and some methods are introduced outside of the regression framework (although they can also be applied to regression). Many methods do not require communication of quantities from the CC to the data nodes—they only require 1 transmission from the nodes to the CC. Given the lack of awareness regarding HPSA, starting by implementing lower–operational complexity methods while providing useful results offers a promising path.

LHSs seek to improve health by generating knowledge during practice and making use of that knowledge to improve practice. This requires analyzing data from a variety of sources. HPSA methods offer a way to tackle the challenges that come with multi-jurisdictional data.

The methods can be implemented “manually” (eg, via email exchanges), but platforms enabling semiautomated distributed fittings of statistical models have been proposed in the literature [60]. As LHSs work by continuously monitoring and analyzing data rather than through cross-sectional studies only, automated platforms are necessary. Ideally, these platforms should be able to link and standardize data sources from multiple jurisdictions as well as perform HPSA. On the other hand, explicit descriptions of these methods’ algorithms and the quantities exchanged are not always easily accessible, and this complicates the evaluation of the tools by data custodians and researchers.

This is especially important as it is essential to clarify here that operating an HPSA algorithm does *not* ensure confidentiality in and of itself.

For example, it is known that sharing sample moments can compromise confidentiality. It can be shown that a set of *n* observations is uniquely determined by its first *n* sample moments [61]. This could prove problematic for methods that rely on sharing the first few moments of each node’s sample, especially if the number of observations is low, as the sample could be partially reconstructed by the CC.

The results presented in this paper contribute to this objective by clarifying the workflows and quantities exchanged in each method. Nevertheless, further analysis of the confidentiality preserved by HPSA methods is needed to fully understand the risk associated with the sharing of summary statistics, especially as more rounds of communication between the CC and data nodes are completed. The framework of differential privacy has been used to guarantee the preservation of confidentiality in a few HPSA methods, but a wider application of differential privacy to existing and popular methods has yet to be explored.

## Supplementary material

10.2196/53622 Multimedia Appendix 1 Detailed protocol for the scoping review.

10.2196/53622 Multimedia Appendix 2 Mathematical derivations pertaining to objective 3.

10.2196/53622 Checklist 1 PRISMA-ScR (Preferred Reporting Items for Systematic Reviews and Meta-Analyses extension for Scoping Reviews) checklist.

## Abbreviations

CC: coordinating center
GLM: generalized linear model
HPSA: horizontally partitioned statistical analytics
LHS: learning health system
MLE: maximum likelihood estimator
