# Correction: Distributed Statistical Analyses: A Scoping Review and Examples of Operational Frameworks Adapted to Health Analytics

**DOI:** 10.2196/71249

**Published:** 2025-02-11

**Authors:** Félix Camirand Lemyre, Simon Lévesque, Marie-Pier Domingue, Klaus Herrmann, Jean-François Ethier

**Affiliations:** 1 GRIIS, Université de Sherbrooke, 2500, boul. de l'Université, Sherbrooke, QC, J1K 2R1, Canada, 1 819 821-8000 ext 74977; 2 Département de mathématiques, Faculté des sciences, Université de Sherbrooke, Sherbrooke, QC, Canada; 3 Health Data Research Network Canada, Vancouver, BC, Canada; 4 Chaire MEIE Québec - Le numérique au service des systèmes de santé apprenants, Université de Sherbrooke, Sherbrooke, QC, Canada; 5 Département de médecine, Faculté de médecine et des sciences de la santé, Université de Sherbrooke, Sherbrooke, QC, Canada

In “Distributed Statistical Analyses: A Scoping Review and Examples of Operational Frameworks Adapted to Health Analytics” (JMIR Med Inform 2024;12:e53622) the authors noted four errors.

In subsection “Results Related to Objective 3,” paragraph “Simple Averaging,” three mathematical formulas have been corrected to include a matrix inversion as follows:

In “Textbox 1 . Algorithm 1 —simple averaging inference procedure,”

${\hat{\Sigma }}_{SA}={\hat{\phi }}_{\text{SA}}\sum_{k=1}^{K}\frac{nw^{\left(k\right)2}}{n^{\left(k\right)}}V_{\text{MLE}}^{\left(k\right)}$

has been corrected to

${\hat{\Sigma }}_{SA}={\hat{\phi }}_{\text{SA}}\sum_{k=1}^{K}\frac{nw^{\left(k\right)2}}{n^{\left(k\right)}}{\left(V_{\text{MLE}}^{\left(k\right)}\right)}^{-1}$.

In the text below Textbox 1,

$\Sigma_{SA}=\phi^{⋆}\sum_{k=1}^{K}\frac{w^{\left(k\right)2}}{p^{\left(k\right)}}T_{\beta^{⋆}}^{\left(k\right)}$

was changed to

$\Sigma_{SA}=\phi^{⋆}\sum_{k=1}^{K}\frac{w^{\left(k\right)2}}{p^{\left(k\right)}}{\left(T_{\beta^{⋆}}^{\left(k\right)}\right)}^{-1}$

and ${\hat{\Sigma }}_{SA}={\hat{\phi }}_{\text{SA}}\sum_{k=1}^{K}\frac{nw^{(k)2}}{n^{\left(k\right)}}V_{\text{MLE}}^{\left(k\right)}$

has been corrected to

${\hat{\Sigma }}_{SA}={\hat{\phi }}_{\text{SA}}\sum_{k=1}^{K}\frac{nw^{\left(k\right)2}}{n^{\left(k\right)}}{\left(V_{\text{MLE}}^{\left(k\right)}\right)}^{-1}$.

Finally, in Multimedia Appendix 2,

$\Sigma_{\text{SA}}=\phi^{⋆}\sum_{k=1}^{K}\frac{w^{\left(k\right)2}}{p^{\left(k\right)}}T_{\beta^{⋆}}^{\left(k\right)}$

has been corrected to

$\Sigma_{\text{SA}}=\phi^{⋆}\sum_{k=1}^{K}\frac{w^{(k)2}}{p^{\left(k\right)}}{\left(T_{\beta^{⋆}}^{\left(k\right)}\right)}^{-1}$.

Please note that the revised version has been attached here as Multimedia Appendix 1.

The correction will appear in the online version of the paper on the JMIR Publications website on February 11, 2025, together with the publication of this correction notice. Because this was made after submission to PubMed, PubMed Central, and other full-text repositories, the corrected article has also been resubmitted to those repositories.

## Supplementary material

10.2196/71249 Multimedia Appendix 1 Revised version of Multimedia Appendix 2: Mathematical derivations pertaining to objective 3.

