# Supplementary material for: Distributed Statistical Analyses: A Scoping Review and Examples of Operational Frameworks Adapted to Health Analytics
Source: JMIR Med Inform. 2024 Nov 14;12:e53622. doi: 10.2196/53622 (PMC11617597; doi:10.2196/53622)
Supplement: Multimedia Appendix 1 [file medinform-v12-e53622-s001.docx]

**Appendix A Detailed protocol for the scoping review**

**Research question**

1. What are the existing methods that allow to conduct statistical inference procedures from a horizontally distributed dataset?
   - *Regarding: Methods for different statistical models; Methods for various settings in terms of information shared; Methods for different needs in terms of precision of estimates.*
2. What are the characteristics of these methods to proceed to a systematic categorisation?
   - *Regarding: Type of algorithm; Settings for nodes and coordinating centre; Capacity to reach exact estimates from data pooling.*

**Methods**

The scoping review will be conducted in accordance with the methodological framework from Levac et al. [10] (based on Arksey and O’Malley [6]). The PRISMA Extension for Scoping Reviews (PRISMA-ScR) checklist was followed to ensure consistency of the process.

***Key-words***

The following keywords were identified from the **snowballing literature search**:

- *distributed algorithms* [11]
- *distributed estimation* [24]
- *distributed inference* [25]
- *distributed learning* [54]
- *distributed regression* [55] (not included in the scoping review final selection since no new estimation methods are discussed).
- *federated inference* [56] (not included in the scoping review final selection since the paper was not published when the scoping review search was launched)
- *federated estimation* [57] (not included in the scoping review final selection since the paper was not published when the scoping review search was launched)
- *federated learning* [58] (not included in the scoping study review final selection the paper focuses solely on estimation, i.e. no confidence interval computation strategies or hypothesis testing framework are discussed).
- *privacy-protecting algorithm* [36]
- *privacy-preserving algorithm* [2]
- *aggregated inference* [4] (not included in the scoping review final selection since no new estimation methods are discussed).

The following keywords will be used to add conciseness to the topic of statistical inference, to avoid screening machine-learning specific articles:

- *Statistical inference*
- *Confidence interval*
- *Statistical estimation*
- *Hypothesis tests*
- *Significant coefficient, Significance of parameter*

***Research strategies***

In collaboration with a specialist in documentary research at the Université de Sherbrooke, we have selected the following abstract and citation databases: (1) Medline, (2) Scopus, (3) MathSciNet, and (4) zbMATH. The choice of these databases was motivated by the interdisciplinary nature of the research question, which spans the fields of statistics and health.

To develop comprehensive research strategies, we combined the previously mentioned keywords and worked closely with the documentary research specialist. The most recent research was executed on May 12, 2023.

Limits and restrictions

In order to strike a balance between sensitivity and specificity in our research, given the interdisciplinary nature of the topic involving distributed data and statistical inference, we took several considerations into account.

To ensure sensitivity, we opted for interdisciplinary databases that are known to cover a wide range of relevant literature. These include Medline, Scopus, MathSciNet, and zbMATH. By selecting these databases, we aimed to capture a comprehensive set of articles that encompass both statistical and health-related aspects.

On the other hand, to maintain specificity and avoid retrieving a large number of non-relevant articles, we carefully selected keywords that were targeted and specific to our research question. Instead of relying solely on thesauri and synonym search tools, we focused on the vocabulary commonly used in the literature through an extensive overlook (snowballing) approach, particularly for the concept of distributed data. For the concept of statistical inference, we chose synonyms that specifically capture studies centred around this topic.

Furthermore, to keep the scope of our research manageable and relevant to recent developments, we limited our search to articles published since the year 2000. This restriction is justified by the emergence of distributed data in recent years, driven by advancements in technology and the availability of massive datasets. By setting this threshold, we aimed to capture any early-developed methods and approaches related to our research topic.

Overall, our research strategies were designed to strike a balance between sensitivity and specificity, ensuring that we capture a comprehensive range of relevant articles while minimizing the inclusion of non-relevant ones.

Medline search query

( ( AB ( ((("Privacy-preserving" OR "Privacy-protecting*" OR "federated" OR "Distributed" OR "aggregated") N1 ("estimation*" OR "algorithm*" OR "inference" OR "analy*" OR "regression*" OR "model*" OR "statistic*" OR "learning"))) OR TI ( ((("Privacy-preserving" OR "Privacy-protecting*" OR "federated" OR "Distributed" OR "aggregated") N1 ("estimation*" OR "algorithm*" OR "inference" OR "analy*" OR "regression*" OR "model*" OR "statistic*" OR "learning"))) ) OR SU ( ((("Privacy-preserving" OR "Privacy-protecting*" OR "federated" OR "Distributed" OR "aggregated") N1 ("estimation*" OR "algorithm*" OR "inference" OR "analy*" OR "regression*" OR "model*" OR "statistic*" OR "learning"))) )) AND ( TX ( ("statistical inference" OR "confidence interval*" OR "Statistical Estimat*" OR "hypothesis test*" OR "significant coefficient*" OR "significant parameter*")) ) )

Scopus search query

TITLE-ABS-KEY (("Privacy-preserving" OR "Privacy-protecting*" OR "federated" OR "Distributed" OR "aggregated") W/1 ("estimation*" OR "algorithm*" OR "inference" OR "analy*" OR "regression*" OR "model*" OR "statistic*" OR "learning") AND ("statistical inference" OR "confidence interval*" OR "Statistical Estimat*" OR "hypothesis test*" OR "significant coefficient*" OR "significant parameter*"))

MathSciNet search query

"Anywhere=("Privacy-preserving" OR "Privacy-protecting" OR "federated" OR "Distributed" OR "aggregated") AND Anywhere=("estimation*" OR "algorithm*" OR "inference" OR "analy*" OR "regression*" OR "model*" OR "statistic*" OR "learning") AND Anywhere=("statistical inference" OR "confidence interval*" OR "Statistical Estimat*" OR "hypothesis test*" OR "significant coefficient*" OR "significant parameter*")"

zbMATH search query

( (ti:("Privacy-preserving" | "Privacy-protecting" | "federated" | "Distributed" | "aggregated") \& ti:("estimation*" | "algorithm*" | "inference" | "analy*" | "regression*" | "model*" | "statistic*" | "learning")) | (ut:("Privacy-preserving" | "Privacy-protecting" | "federated" | "Distributed" | "aggregated") \& ut:("estimation*" | "algorithm*" | "inference" | "analy*" | "regression*" | "model*" | "statistic*" | "learning")) ) \& any:("statistical inference" | "confidence interval*" | "Statistical Estimat*" | "hypothesis test*" | "significant coefficient*" | "significant parameter*")

Grey literature

As one of the exclusion criteria is to exclude all unpublished studies, no research was conducted among grey literature.

***Selection process***

After removing duplicate references, a manual review of the selected references obtained from the databases was conducted to identify relevant articles that address the research question. In this study, a two-stage selection process was employed to ensure a thorough and systematic approach.

To ensure consistency and minimize bias, all reviewers involved in the selection process met before the commencement of the first stage of selection. This initial meeting aimed to establish a shared understanding of the inclusion criteria and research objectives. By aligning their interpretations and definitions of the inclusion criteria, the reviewers ensured a consistent approach throughout the selection process.

During the selection process, there has been a midpoint meeting among the reviewers after the completion of the first stage of selection. This meeting served as an opportunity to discuss any questions, challenges, or uncertainties that may have arisen during the initial selection. By addressing these issues collectively, the reviewers maintained consistency and addressed discrepancies in their evaluations.

Finally, at the end of the second stage of selection, the reviewers had a final meeting. This meeting allowed for a comprehensive discussion of the selected references and ensured that the final set of included articles met the predefined criteria and effectively addressed the research question.

By conducting regular meetings throughout the selection process and discussing the inclusion criteria, the reviewers aimed to maintain consistency, minimize subjectivity, and enhance the reliability of the article selection.

***Stages of the selection***

Selection 1: Titles and Abstracts

All titles and abstracts of the references identified through the research strategy were evaluated by a single author (MPD or FCL). Since this step involved a single reviewer, references that were clearly unrelated to the research question or did not meet the inclusion criteria were automatically excluded from further consideration.

The evaluation process conducted by the single author aimed to swiftly discard references that were obviously irrelevant to the research question. This initial screening helped streamline the subsequent stages of the selection process by removing references that did not align with the study’s objectives or criteria.

Selection 2: Full text

The full texts of the references selected in the first stage were reviewed by two authors (MPD and FCL). In instances where there were differing opinions between the two initial reviewers, they engaged in discussions to reach a consensus. To ensure impartiality and a final resolution, a third author (JFE) conducted a third review, overseeing the process and making the ultimate decision in cases where disagreements persisted.

Additional strategy

The list of references from all the included articles after the selection process was carefully assessed to identify any additional articles that may not have been captured during the initial screening due to specific keywords. This step aimed to ensure a comprehensive approach by exploring the reference lists of the included articles for relevant references that might have been missed in the initial search.

Through this approach, the review aimed to minimize the possibility of excluding relevant studies and to provide a comprehensive and robust synthesis of the available literature on the subject matter.

Inclusion criteria

The following criteria were utilized to guide the selection process. Exclusion was considered for a reference if it met at least one of the exclusion criteria, or if it failed to meet at least one of the inclusion criteria.

Table 4. Inclusion and Exclusion criteria.

| **Criteria Topic** | **Inclusion criteria** | **Exclusion criteria** |
| --- | --- | --- |
| 1. Horizontally distributed data | This paper/study presents a solution for performing inferential statistics on horizontally distributed data.  *Examples of papers that would not meet the criteria: the method is presented on vertically distributed data, or the method is presented on horizontally distributed studies instead of distributed* data. | - |
| 2. Inferential statistics | - | The paper/study does not specifically address inferential statistics (Confidence intervals, Hypothesis testing or Asymptotic normality result).  *e.g., the focus is not on estimation and/or confidence intervals and/or hypothesis testing.* |
| 3. Methodological contribution | - | The paper/study does not provide a new methodological contribution.  *e.g., the study is solely an application of a previously developed and presented method.* |
| 4. Discussion paper | - | The article is a discussion paper. |
| 5. Published Study | - | The paper/study has not been published. |
| 6. Encryption | - | The paper/study presents a solution for encryption or secret-sharing. |
| 7. Language | - | The full text is not available in English or French. |

***Data-charting***

A data-charting form was collaboratively developed to facilitate the extraction of relevant information from the selected studies. The extraction process was conducted manually, with two authors (MPD and FCL) independently extracting data from the first five studies. Subsequently, the authors convened to verify the adequacy of the process and ensure consistency in data extraction. The remaining studies were then divided between the two authors for data extraction.

During the data extraction phase, specific information pertaining to the research questions was identified and recorded. To account for any uncertainties or variables requiring additional review, a "To be determined" modality was included for each extracted variable. This modality serves as a reminder for a second author to review and validate the extracted data, ensuring accuracy and reliability.

Table 5. Data extraction.

| **Variable collected** | **Modalities** |
| --- | --- |
| 1. Model type | Parametric regression; Semi-Parametric regression; Non-parametric regression; Not specific to regression; To be determined |
| 2. Methodological setting | Big or Massive/Multi-machines setting; Healthcare; Other; To be determined |
| 3. Communication from coordinating centre to nodes | Yes; No; To be determined |
| 4. Equal to the pooled solution | Yes; No; Many types are discussed; To be determined |
| 5. GLM | GLM not addressed; Only linear regression is addressed; Only logistic regression is addressed; GLM are addressed (linear regression and logistic regression, and/or others); To be determined |
| 6. Type of coordinating centre | External to the nodes; One of the nodes; Both are discussed; Not mentionned; To be determined |
| 7. Specific method | *Name of the method as presented* |
