# Supplementary material for: Distributed Statistical Analyses: A Scoping Review and Examples of Operational Frameworks Adapted to Health Analytics
Source: JMIR Med Inform. 2024 Nov 14;12:e53622. doi: 10.2196/53622 (PMC11617597; doi:10.2196/53622)
Supplement: Multimedia Appendix 2 [file medinform-v12-e53622-s002.docx]

**Appendix B** **Mathematical derivations pertaining to Objective 3**

**Notations used in the Appendix**

Recall that in the current setting, there are $n$ individuals horizontally partitioned across $K$ data storage nodes. Each node’s dataset is $\mathcal{D}^{\left( k \right)}=\{\mathbf{z}_{i}^{\left( k \right)}=\left( x_{1i}^{\left( k \right)},\ldots,x_{pi}^{\left( k \right)},y_{i}^{\left( k \right)} \right)^{\top}{\}}_{i=1}^{n^{\left( k \right)}}$, where $1\leq k\leq K$ and $\mathbf{z}_{i}^{\left( k \right)}$ represents measurements on the $i^{\text{th}}$ individual at node $k$: $y_{i}^{\left( k \right)}\mathbb{\in R}$ denotes their response variable and $\left[ x_{1i}^{\left( k \right)},\ldots,x_{pi}^{\left( k \right)} \right]^{\top}\in\mathbb{R}^{p}$ denotes their covariate vector. $n^{\left( k \right)}$ is the total sample size at node $k$. The combined datasets $\mathcal{D}^{\left( 1 \right)},\ldots,\mathcal{D}^{\left( K \right)}$ make up the whole dataset without any duplicated individuals such that $\sum_{k=1}^{K} n^{\left( k \right)}=n$.

The current GLM framework assumes that there exists unknown parameters $\boldsymbol{\beta}^{\star}\in\mathbb{R}^{p+1}\mathbb{\in R}$ and $\phi^{\star}>0$, and known model-specific functions $b,c,g,h$ such that with $\mathbf{x}_{i}^{\left( k \right)}=\left[ x_{0i}^{\left( k \right)},x_{1i}^{\left( k \right)},\ldots,x_{pi}^{\left( k \right)} \right]^{\top}$ and $x_{0i}^{\left( k \right)}=1$, we have $y_{i}^{\left( k \right)}\mid\mathbf{x}_{i}^{\left( k \right)}\sim f\left( \cdot;\mathbf{x}_{i}^{\left( k \right)},\boldsymbol{\beta}^{\star},\phi^{\star} \right)$, where for any $\boldsymbol{\beta}=\left[ \beta_{0},\beta_{1},\ldots,\beta_{p} \right]^{\top}\in\mathbb{R}^{p+1}$ and $\phi$,

| $f\left( y;\mathbf{x}_{i}^{\left( k \right)},\boldsymbol{\beta},\phi\right)=exp\left[ \frac{yh\left( \boldsymbol{\beta}^{\top}\mathbf{x}_{i}^{\left( k \right)} \right)-b\left\{ h\left( \boldsymbol{\beta}^{\top}\mathbf{x}_{i}^{\left( k \right)} \right) \right\}}{\phi}+c\left( y,\phi\right) \right] ,$ |  |
| --- | --- |

where $b$ is such that $b'\left\{ h\left( \boldsymbol{\beta}^{\top}\mathbf{x}_{i}^{\left( k \right)} \right) \right\}=E\left( y_{i}^{\left( k \right)} \right)=g^{\left( -1 \right)}\left( \boldsymbol{\beta}^{\top}\mathbf{x}_{i}^{k} \right)$, with $b'\left( x \right)=\partial b\left( x \right)/\partial x$.

We also recall the definition of $\mathbf{D}^{\left( k \right)}\left( \boldsymbol{\beta} \right)\in\mathbb{R}^{p+1}$:

| $\mathbf{D}^{\left( k \right)}\left( \boldsymbol{\beta} \right)=\frac{1}{n^{\left( k \right)}}\sum_{i=1}^{n^{\left( k \right)}} \mathbf{x}_{i}^{\left( k \right)} h'\left( \boldsymbol{\beta}^{\top}\mathbf{x}_{i}^{\left( k \right)} \right)\left[ y_{i}^{\left( k \right)}-b'\{h\left( \boldsymbol{\beta}^{\top}\mathbf{x}_{i}^{\left( k \right)} \right)\} \right] ,$ |  |
| --- | --- |

as well as the one of $\mathbf{V}^{\left( k \right)}\left( \boldsymbol{\beta} \right)$:

| $\begin{matrix} \mathbf{V}^{\left( k \right)}\left( \boldsymbol{\beta} \right) & =\frac{1}{n^{\left( k \right)}}\sum_{i=1}^{n^{\left( k \right)}} \mathbf{x}_{i}^{\left( k \right)}\left( \mathbf{x}_{i}^{\left( k \right)} \right)^{\top}[h'\left( \boldsymbol{\beta}^{\top}\mathbf{x}_{i}^{\left( k \right)} \right)^{2} b''\{h\left( \boldsymbol{\beta}^{\top}\mathbf{x}_{i}^{\left( k \right)} \right)\} \\ & -h''\left( \boldsymbol{\beta}^{\top}\mathbf{x}_{i}^{\left( k \right)} \right)(y_{i}^{\left( k \right)}-b'\{h\left( \boldsymbol{\beta}^{\top}\mathbf{x}_{i}^{\left( k \right)} \right)\}] . \end{matrix}$ |  |
| --- | --- |

Finally, let us reiterate the definitions of $E^{\left( k \right)}$ in equation (4) and $F^{\left( k \right)}$ in equation (5), which are expressed as follows:

| $E^{\left( k \right)}\left( \phi,\boldsymbol{\beta} \right)=\frac{1}{n^{\left( k \right)}}\sum_{i=1}^{n^{\left( k \right)}} \left[ y_{i}^{\left( k \right)}h\left( \boldsymbol{\beta}^{\top}\mathbf{x}_{i}^{\left( k \right)} \right)-b\{h\left( \boldsymbol{\beta}^{\top}\mathbf{x}_{i}^{\left( k \right)} \right)\} \right]-\frac{\phi^{2}}{n^{\left( k \right)}}\sum_{i=1}^{n^{\left( k \right)}} \frac{\partial}{\partial\phi}c\left( y_{i}^{\left( k \right)},\phi\right) .$ |  |
| --- | --- |

and

| $F^{\left( k \right)}\left( \phi,\boldsymbol{\beta} \right)=\frac{2\phi}{n^{\left( k \right)}}\sum_{i=1}^{n^{\left( k \right)}} \frac{\partial}{\partial\phi}c\left( y_{i}^{\left( k \right)},\phi\right)+\frac{\phi^{2}}{n^{\left( k \right)}}\sum_{i=1}^{n^{\left( k \right)}} \frac{\partial^{2}}{\partial\phi^{2}}c\left( y_{i}^{\left( k \right)},\phi\right) .$ |  |
| --- | --- |

**General estimation in a pooled centralized setting**

The likelihood of the full dataset $\mathcal{D=}\cup_{i=1}^{K}\mathcal{D}^{\left( k \right)}$, in a setting where the likelihood contribution of each node would be given by the set of weights $\{w^{\left( k \right)}{\}}_{k=1}^{K}$, is given by

| $\sum_{k=1}^{K} w^{\left( k \right)}\mathcal{l}^{\left( k \right)}\left( \boldsymbol{\beta},\phi\right)=\sum_{k=1}^{K} w^{\left( k \right)}\sum_{i=1}^{n^{\left( k \right)}} \left[ \frac{y_{i}^{\left( k \right)}h\left( \boldsymbol{\beta}^{\top}\mathbf{x}_{i}^{\left( k \right)} \right)-b\left\{ h\left( \boldsymbol{\beta}^{\top}\mathbf{x}_{i}^{\left( k \right)} \right) \right\}}{\phi}+c\left( y_{i}^{\left( k \right)},\phi\right) \right] .$ | $\left( 15 \right)$ |
| --- | --- |

Pooled maximum likelihood estimates of $\boldsymbol{\beta}^{\star}$ and $\phi^{\star}$ are found by calculating a set of values $({\hat{\boldsymbol{\beta}}}_{\text{Pooled}}$, $\hat{\phi}_{\text{Pooled}})$ that maximizes (15). This is usually done in two steps. In a first step, equating the gradient with respect to the $\boldsymbol{\beta}$ parameters to 0 yields a set of equations that are independent of $\phi$ which, in our framework, are given by

| $\sum_{k=1}^{K} w^{\left( k \right)}\mathbf{D}^{\left( k \right)}\left( \boldsymbol{\beta} \right)=0 .$ |  |
| --- | --- |

As $g^{\left( -1 \right)}$ is often non-linear, iterative methods are necessary to solve the latter equations. When a solution exists and is unique (this is the case under general conditions [59]), the resulting estimator ${\hat{\boldsymbol{\beta}}}_{\text{Pooled}}$ is called the *maximum likelihood estimator*.

In a second step, using ${\hat{\boldsymbol{\beta}}}_{\text{Pooled}}$, a maximum likelihood estimator of $\phi^{\star}$ can be obtained by solving

| $\sum_{k=1}^{K} w^{\left( k \right)}E^{\left( k \right)}\left( \phi,{\hat{\boldsymbol{\beta}}}_{\text{Pooled}} \right)=0 .$ | $\left( 16 \right)$ |
| --- | --- |

The above equations can be further reduced when $b'\{h\left( \boldsymbol{\beta}^{\top}\mathbf{x}_{i}^{\left( k \right)} \right)\}=g^{\left( -1 \right)}\left( \boldsymbol{\beta}^{\top}\mathbf{x}_{i}^{\left( k \right)} \right)$, which happens when $g$ is canonical, since in this case, $h\left( x \right)\equiv x$.

When $\phi^{\star}$ is unknown, it can be estimated by differentiating the log-likelihood at $\left( {\hat{\boldsymbol{\beta}}}_{\text{Pooled}},\phi\right)$ with respect to $\phi$ and equating it to $0$. Indeed, since the likelihood equations of $\boldsymbol{\beta}$ do not involve $\phi$, it always holds that

| $\max_{\boldsymbol{\beta},\phi}\mathcal{l}\left( \boldsymbol{\beta},\phi\mathcal{,D} \right)=\max_{\phi}\mathcal{l}\left( {\hat{\boldsymbol{\beta}}}_{\text{Pooled}},\phi\mathcal{,D} \right) .$ |  |
| --- | --- |

Proceeding in this way yields the following equation for a maximum likelihood estimator of $\phi$ to satisfy:

| $\sum_{k=1}^{K} \frac{w^{\left( k \right)}}{n^{\left( k \right)}}\sum_{i=1}^{n^{\left( k \right)}} \left( y_{i}^{\left( k \right)}h\{\left( {\hat{\boldsymbol{\beta}}}_{\text{Pooled}} \right)^{\top}\mathbf{x}_{i}^{\left( k \right)})\}-b[h\{{\hat{\boldsymbol{\beta}}}_{\text{Pooled}})^{\top}\mathbf{x}_{i}^{\left( k \right)}\}] \right)=\phi^{2}\sum_{k=1}^{K} \frac{w^{\left( k \right)}}{n^{\left( k \right)}}\sum_{i=1}^{n^{\left( k \right)}} \frac{\partial}{\partial\phi}c\left( y_{i}^{\left( k \right)},\phi\right) .$ |  |
| --- | --- |

**Calculations related to unequal sample sizes and uneven between-nodes covariate distributions**

The theoretical validity of each algorithm presented in the section Results related to objective O3 relies on two main components:

1. An asymptotic normality result for the estimator of the $\boldsymbol{\beta}$ parameters involved;
2. The consistency, i.e., convergence in probability to the true value, of the estimator for the asymptotic variance-covariance matrix involved in the aforementioned asymptotic normality result. This, in turn, depends on the consistency of the estimator of $\phi$ when the latter is unknown.

Since the current paper is already quite extensive, we will provide theoretical arguments for the asymptotic normality result only, as it is arguably the most interesting from a theoretical perspective. The proof of consistency of the variance-covariance matrix is a lengthy and technical exercise that can be accomplished using our arguments in combination with standard M-estimation theorems, which can be found, for example, in [60], chapter 5.

***Conditions used to establish asymptotic normality results***

The following conditions will be used. For $\mathcal{l\in\{}0,1,2,3\}$, let

| $h_{\mathcal{l}}\left( x \right)=\frac{\partial^{\mathcal{l}}}{\partial x^{\mathcal{l}}}h\left( x \right) \left( b'\circ h \right)_{\mathcal{l}}\left( x \right)=\frac{\partial^{\mathcal{l}}}{\partial x^{\mathcal{l}}}\left( b'\circ h \right)\left( x \right) \left( b''\circ h \right)_{\mathcal{l}}\left( x \right)=\frac{\partial^{\mathcal{l}}}{\partial x^{\mathcal{l}}}\left( b''\circ h \right)\left( x \right) .$ |
| --- |

Also, in what follows, for any vector $\mathbf{a}\in\mathbb{R}^{p+1}$, one defines $\|\mathbf{a}{\|}_{\infty}=\max_{1\leq j\leq p+1}|\left[ \mathbf{a} \right]_{j}|$ and $\|\mathbf{a}{\|}_{1}=\sum_{j=1}^{p+1} |\left[ \mathbf{a} \right]_{j}|$.

**Conditions C**

1. For $k\in\{1,\ldots,K\}$, $n^{\left( k \right)}/n\to p^{\left( k \right)}>0$ as $n\to\infty$, and $K\geq2$ is finite;
2. $b$ and $h$ are three times continuously differentiable;
3. For $k\in\{1,\ldots,K\}$, $\{\left[ x_{i1}^{\left( k \right)},\ldots,x_{ip}^{\left( k \right)} \right]^{\top}{\}}_{i=1}^{n^{\left( k \right)}}$ is a set of i.i.d. random vectors with finite sixth marginal moments, i.e., $E\left\{ |\mathbf{x}_{i}^{\left( k \right)}|_{j}^{6} \right\}<\infty$, and $E\left\{ |y_{i}^{\left( k \right)}|^{4} \right\}<\infty$. Further, $\mathcal{T}_{\boldsymbol{\beta}^{\star}}^{\left( k \right)}$ is positive definite, where

| $\begin{matrix} \left[ \mathcal{T}_{\boldsymbol{\beta}^{\star}}^{\left( k \right)} \right]_{jl} & =E[x_{ij}^{\left( k \right)} x_{il}^{\left( k \right)}h'\left\{ \left( \boldsymbol{\beta}^{\star} \right)^{\top}\mathbf{x}_{i}^{\left( k \right)}{\}}^{2} b''\{h\{\left( \boldsymbol{\beta}^{\star} \right)^{\top}\mathbf{x}_{i}^{\left( k \right)}\}\} \right] . \end{matrix}$ | $\left( 17 \right)$ |
| --- | --- |

1. The $\boldsymbol{\beta}$-parameter space $\Theta\subset\mathbb{R}^{p+1}$ considered for the search of $\boldsymbol{\beta}^{\star}$ is compact, and $\boldsymbol{\beta}^{\star}$ lies in the interior of $\Theta$. Further, one has $E\{\mathbf{D}^{\left( k \right)}\left( \boldsymbol{\beta} \right)\}=0$ if and only if $\boldsymbol{\beta}=\boldsymbol{\beta}^{\star}$.
2. For $\mathcal{l\in\{}0,1,2,3\}$, $E\left\{ \Upsilon_{\mathcal{l}}^{4}\left( \mathbf{x}_{i}^{\left( k \right)} \right) \right\}<\infty$, where $\Upsilon_{\mathcal{l}}\left( \mathbf{x} \right)=\sup_{\boldsymbol{\beta}\in\Theta}|h_{\mathcal{l}}\left( \boldsymbol{\beta}^{\top}\mathbf{x} \right)|$. Moreover, for $\mathcal{l\in\{}0,1\}$, $E\left\{ \tilde{\Upsilon}_{\mathcal{l}}^{4}\left( \mathbf{x}_{i}^{\left( k \right)} \right) \right\}<\infty$ and $E\left\{ \overline{\Upsilon}_{\mathcal{l}}^{4}\left( \mathbf{x}_{i}^{\left( k \right)} \right) \right\}<\infty$, where $\tilde{\Upsilon}_{\mathcal{l}}\left( \mathbf{x} \right)=\sup_{\boldsymbol{\beta}\in\Theta}|\left( b'\circ h \right)_{\mathcal{l}}\left( \boldsymbol{\beta}^{\top}\mathbf{x} \right)|$ and $\overline{\Upsilon}_{\mathcal{l}}\left( \mathbf{x} \right)=\sup_{\boldsymbol{\beta}\in\Theta}|\left( b''\circ h \right)_{\mathcal{l}}\left( \boldsymbol{\beta}^{\top}\mathbf{x} \right)|$.

Assumption (C1) states that each data node has a non-negligible proportion of the data. Assumption (C2) imposes a smoothness condition on the known quantities involved in the definition of the GLM, enabling the use of standard theoretical arguments to derive the asymptotic normality of the estimated coefficients. It is not restrictive. The assumption (C3) that the within-node predictor distribution is the same across all individuals is made to simplify the arguments and to make them more concise. It could be relaxed in various ways, for example, by assuming equal first and second-order moments of relevant quantities instead of the entire distribution.

The compactness of $\Theta$ in Condition (C4) is used to establish that $\mathbf{D}^{\left( k \right)}\left( \boldsymbol{\beta} \right)$ and $\mathbf{V}^{\left( k \right)}\left( \boldsymbol{\beta} \right)$ are uniformly consistent across all possible values for $\boldsymbol{\beta}^{\star}$, which is a commonly used assumption in maximum likelihood estimation. The identification condition ensures that $\boldsymbol{\beta}^{\star}$ is the unique value that maximizes the expectation of the node-specific likelihood.

Assumption (C5) is a technical requirement to establish a uniform consistency result for $\mathbf{D}^{\left( k \right)}\left( \boldsymbol{\beta} \right)$ and $\mathbf{V}^{\left( k \right)}\left( \boldsymbol{\beta} \right)$. It is satisfied when the first, second, and third-order derivatives of $h$ and $b$ are bounded, as long as $E\{\parallel\mathbf{x}_{i}^{\left( k \right)}\parallel_{1}\}<\infty$. More generally, it imposes a condition on the tails of the distribution of the $\mathbf{x}_{i}^{\left( k \right)}$’s. For example, in Poisson regression, where $h\left( x \right)=x$ and $b'\left( x \right)=e^{x}$, this assumption is satisfied if $E\left( \parallel\mathbf{x}_{i}^{\left( k \right)}\parallel_{1}e^{\boldsymbol{\beta}_{\text{MAX}}^{\top}\mathbf{x}_{i}^{\left( k \right)}} \right)<\infty$, where $\boldsymbol{\beta}_{\text{MAX}}=\sup_{\boldsymbol{\beta}\in\Theta}\parallel\boldsymbol{\beta}\parallel_{\infty}\left( 1,\ldots,1 \right)^{\top}$. This condition holds, for example, when the $\mathbf{x}_{i}^{\left( k \right)}$’s are normally distributed or have compact support.

***Theory for the pooled centralized setting estimator***

Proceeding as in the proof of Lemma 5 one can show that ${\hat{\boldsymbol{\beta}}}_{\text{Pooled}}=\boldsymbol{\beta}^{\star}+o_{\mathbb{P}}\left( 1 \right)$. From there, one has, in view of Lemma 7, that

| $\begin{matrix} \sum_{k=1}^{K} w^{\left( k \right)}\mathbf{D}^{\left( k \right)}\left( \boldsymbol{\beta}^{\star} \right) & =\sum_{k=1}^{K} w^{\left( k \right)}\left\{ \mathbf{D}^{\left( k \right)}\left( \boldsymbol{\beta}^{\star} \right)-\mathbf{D}^{\left( k \right)}\left( {\hat{\boldsymbol{\beta}}}_{\text{Pooled}} \right) \right\} \\ & =-\sum_{k=1}^{K} w^{\left( k \right)}\left\{ \mathbf{V}^{\left( k \right)}\left( \boldsymbol{\beta}^{\star} \right)+o_{\mathbb{P}}\left( 1 \right) \right\}\left( \boldsymbol{\beta}^{\star}-{\hat{\boldsymbol{\beta}}}_{\text{Pooled}} \right) . \end{matrix}$ |  |
| --- | --- |

Since $\mathbf{V}^{\left( k \right)}\left( \boldsymbol{\beta}^{\star} \right)=\mathcal{T}_{\boldsymbol{\beta}^{\star}}^{\left( k \right)}+o_{\mathbb{P}}\left( 1 \right)$ (see Lemma 7), and as $\mathbf{D}^{\left( k \right)}$ is $O_{\mathbb{P}}\left( n^{-1/2} \right)$ (see Lemma 2), one obtains that

| $\sqrt{n}\left( {\hat{\boldsymbol{\beta}}}_{\text{Pooled}}-\boldsymbol{\beta}^{\star} \right)=\{\sum_{k=1}^{K} w^{\left( k \right)}\mathcal{T}_{\boldsymbol{\beta}^{\star}}^{\left( k \right)}{\}}^{-1}\sum_{k=1}^{K} w^{\left( k \right)}\sqrt{\frac{n}{n^{\left( k \right)}}}\left\{ \sqrt{n^{\left( k \right)}}\mathbf{D}^{\left( k \right)}\left( \boldsymbol{\beta}^{\star} \right) \right\}+o_{\mathbb{P}}\left( 1 \right) .$ | $\left( 18 \right)$ |
| --- | --- |

Lemma 1 implies $\sqrt{n^{\left( k \right)}}[\mathbf{D}^{\left( k \right)}\left( \boldsymbol{\beta}^{\star} \right)-E\{\mathbf{D}^{\left( k \right)}\left( \boldsymbol{\beta}^{\star} \right)\}]$ converges in distribution to a centred normal random variable with covariance matrix $\phi^{\star}\mathcal{T}_{\boldsymbol{\beta}^{\star}}^{\left( k \right)}$ for each $1\leq k\leq K$. Since the $\mathcal{D}^{\left( k \right)}$’s are mutually independent, as $K$ is finite, and because $n/n^{\left( k \right)}\to1/p^{\left( k \right)}$ as $n\to\infty$, then, in view of the above equation, Slutsky’s theorem ensures that

| $\begin{matrix} \sqrt{n}\left( {\hat{\boldsymbol{\beta}}}_{\text{Pooled}}-\boldsymbol{\beta}^{\star} \right)\to& \mathcal{N}\left( 0,\boldsymbol{\Sigma}_{\text{Pooled}} \right) \\ & \text{where} \boldsymbol{\Sigma}_{\text{Pooled}}=\left( \mathcal{T}_{\boldsymbol{\beta}^{\star}} \right)^{-1}\left\{ \phi^{\star}\sum_{k=1}^{K} \frac{{w^{\left( k \right)}}^{2}}{p^{\left( k \right)}}\mathcal{T}_{\boldsymbol{\beta}^{\star}}^{\left( k \right)} \right\}\left( \mathcal{T}_{\boldsymbol{\beta}^{\star}} \right)^{-1} , \end{matrix}$ |
| --- |

with $\mathcal{T}_{\boldsymbol{\beta}^{\star}}=\sum_{k=1}^{K} w^{\left( k \right)}\mathcal{T}_{\boldsymbol{\beta}^{\star}}^{\left( k \right)}$.

***Theory for the adapted simple averaging estimator***

Since $\sum_{k=1}^{n^{\left( k \right)}} w^{\left( k \right)}=1$, then, using the definition of ${\hat{\boldsymbol{\beta}}}_{\text{SA}}$, one has

| $\begin{matrix} \sqrt{n}\left( {\hat{\boldsymbol{\beta}}}_{\text{SA}}-\boldsymbol{\beta}^{\star} \right)=\sqrt{n}\sum_{k=1}^{K} w^{\left( k \right)}\left( {\hat{\boldsymbol{\beta}}}_{\text{MLE}}^{\left( k \right)}-\boldsymbol{\beta}^{\star} \right)=\sum_{k=1}^{K} w^{\left( k \right)}\sqrt{n/n^{\left( k \right)}}\left\{ \sqrt{n^{\left( k \right)}}\left( {\hat{\boldsymbol{\beta}}}_{\text{MLE}}^{\left( k \right)}-\boldsymbol{\beta}^{\star} \right) \right\} \end{matrix}$ |
| --- |

By Lemma 6, under Conditions (C1) to C5), it holds as $n\to\infty$ that, for all $1\leq k\leq K$, $\sqrt{n^{\left( k \right)}}\left( {\hat{\boldsymbol{\beta}}}_{\text{MLE}}^{\left( k \right)}-\boldsymbol{\beta}^{\star} \right)$ converges in distribution to a centred normal random variable with variance-covariance matrix given by $\phi^{\star}\left( \mathcal{T}_{\boldsymbol{\beta}^{\star}}^{\left( k \right)} \right)^{-1}$. Since the $\mathcal{D}^{\left( k \right)}$’s are mutually independent, as $K$ is finite, and because $n/n^{\left( k \right)}\to1/p^{\left( k \right)}$ as $n\to\infty$, then, in view of the above equation, Slutsky’s theorem ensures that

| $\sqrt{n}\left( {\hat{\boldsymbol{\beta}}}_{\text{SA}}-\boldsymbol{\beta}^{\star} \right)\to N\left( 0,\boldsymbol{\Sigma}_{SA} \right) , \text{where }\boldsymbol{\Sigma}_{SA}=\phi^{\star}\sum_{k=1}^{K} \frac{{w^{\left( k \right)}}^{2}}{p^{\left( k \right)}}\left( \mathcal{T}_{\boldsymbol{\beta}^{\star}}^{\left( k \right)} \right)^{-1} .$ |  |
| --- | --- |

***Theory for the adapted single distributed Newton-Raphson updating estimator***

Let ${\hat{\boldsymbol{\beta}}}_{\text{SNR}}$ denote the single distributed Newton-Raphson updating estimator ${\hat{\boldsymbol{\beta}}}_{\text{NR,1}}$ of $\boldsymbol{\beta}^{\star}$. One has from (10) that

| ${\hat{\boldsymbol{\beta}}}_{\text{SNR}}-{\hat{\boldsymbol{\beta}}}_{\text{SA}}=\{\sum_{k=1}^{K} w^{\left( k \right)}\mathbf{V}^{\left( k \right)}\left( {\hat{\boldsymbol{\beta}}}_{\text{SA}} \right){\}}^{-1}\sum_{k=1}^{K} w^{\left( k \right)}\mathbf{D}^{\left( k \right)}\left( {\hat{\boldsymbol{\beta}}}_{\text{SA}} \right) .$ |  |
| --- | --- |

Since ${\hat{\boldsymbol{\beta}}}_{SA}-\boldsymbol{\beta}^{\star}=O_{\mathbb{P}}\left( n^{-1/2} \right)$,

| $\mathbf{D}^{\left( k \right)}\left( {\hat{\boldsymbol{\beta}}}_{\text{SA}} \right)-\mathbf{D}^{\left( k \right)}\left( \boldsymbol{\beta}^{\star} \right)=\mathbf{V}^{\left( k \right)}\left( {\hat{\boldsymbol{\beta}}}_{\text{SA}} \right)\left( \boldsymbol{\beta}^{\star}-{\hat{\boldsymbol{\beta}}}_{\text{SA}} \right)+o_{\mathbb{P}}\left( n^{-1/2} \right) .$ | $\left( 19 \right)$ |
| --- | --- |

Hence,

| ${\hat{\boldsymbol{\beta}}}_{\text{SNR}}-{\hat{\boldsymbol{\beta}}}_{\text{SA}}=\{\sum_{k=1}^{K} w^{\left( k \right)}\mathbf{V}^{\left( k \right)}\left( {\hat{\boldsymbol{\beta}}}_{\text{SA}} \right){\}}^{-1}\sum_{k=1}^{K} w^{\left( k \right)}\mathbf{D}^{\left( k \right)}\left( \boldsymbol{\beta}^{\star} \right)+\left( \boldsymbol{\beta}^{\star}-{\hat{\boldsymbol{\beta}}}_{\text{SA}} \right)+o_{\mathbb{P}}\left( n^{-1/2} \right) .$ |
| --- |

One concludes by re-arranging terms in the preceding equation that

| ${\hat{\boldsymbol{\beta}}}_{\text{SNR}}-\boldsymbol{\beta}^{\star}=\{\sum_{k=1}^{K} w^{\left( k \right)}\mathbf{V}^{\left( k \right)}\left( {\hat{\boldsymbol{\beta}}}_{\text{SA}} \right){\}}^{-1}\sum_{k=1}^{K} w^{\left( k \right)}\mathbf{D}^{\left( k \right)}\left( \boldsymbol{\beta}^{\star} \right)+o_{\mathbb{P}}\left( n^{-1/2} \right) .$ |  |
| --- | --- |

Since Lemma 7 ensures the relationship $\sum_{k=1}^{K} w^{\left( k \right)}\mathbf{V}^{\left( k \right)}\left( {\hat{\boldsymbol{\beta}}}_{\text{SA}} \right)=\sum_{k=1}^{K} w^{\left( k \right)}\mathcal{T}_{\boldsymbol{\beta}^{\star}}^{\left( k \right)}+o_{\mathbb{P}}\left( 1 \right)$, the right-hand side of the last equation is asymptotically equivalent to the right-hand side of (18). Hence, one concludes that

| $\sqrt{n}\left( {\hat{\boldsymbol{\beta}}}_{\text{SNR}}-\boldsymbol{\beta}^{\star} \right)\mathcal{\to N}\left( 0,\boldsymbol{\Sigma}_{\mathrm{Pooled}} \right) ,$ |  |
| --- | --- |

where $\boldsymbol{\Sigma}_{\mathrm{Pooled}}$ is as above.

***Theory for the adapted multiple distributed Newton-Raphson updating estimator***

Let ${\hat{\boldsymbol{\beta}}}_{\text{MNR}}$ denote the multiple distributed Newton-Raphson updatings estimator. When iterations are conducted until convergence, the obtained estimator of $\boldsymbol{\beta}^{\star}$ is equal to ${\hat{\boldsymbol{\beta}}}_{\text{Pooled}}$. Hence,

| $\begin{matrix} \sqrt{n}\left( {\hat{\boldsymbol{\beta}}}_{\text{MNR}}-\boldsymbol{\beta}^{\star} \right)\to& \mathcal{N}\left( 0,\boldsymbol{\Sigma}_{\text{Pooled}} \right) . \end{matrix}$ |  |
| --- | --- |

***Theory for the distributed estimating equations estimator***

Let ${\hat{\boldsymbol{\beta}}}_{\text{EE}}$ denote the obtained distributed estimating equations estimator. Since it has been established above that ${\hat{\boldsymbol{\beta}}}_{\text{MLE}}^{\left( k \right)}-\boldsymbol{\beta}^{\star}=O_{\mathbb{P}}\left( n^{-1/2} \right)$ one obtains from a multivariate Taylor expansion that it holds uniformly in $k\in\{1,\ldots,K\}$ and as $n\to\infty$ that

| $\mathbf{D}^{\left( k \right)}\left( \boldsymbol{\beta}^{\star} \right)=\mathbf{D}^{\left( k \right)}\left( \boldsymbol{\beta}^{\star} \right)-\mathbf{D}^{\left( k \right)}\left( {\hat{\boldsymbol{\beta}}}_{\text{MLE}}^{\left( k \right)} \right)=-\mathbf{V}_{\text{MLE}}^{\left( k \right)}\left( \boldsymbol{\beta}^{\star}-{\hat{\boldsymbol{\beta}}}_{\text{MLE}}^{\left( k \right)} \right)+o_{\mathbb{P}}\left( n^{-1/2} \right) .$ | $\left( 20 \right)$ |
| --- | --- |

Recalling the definitions of ${\overline{\mathbf{V}}}_{\text{EE}}$ and ${\hat{\boldsymbol{\beta}}}_{\text{EE}}$ from (13) and (14) we hence have

| $\begin{matrix} {\hat{\boldsymbol{\beta}}}_{\text{EE}} & ={\overline{\mathbf{V}}}_{\text{EE}}^{-1}\sum_{k=1}^{K} w^{\left( k \right)}\mathbf{V}_{\text{MLE}}^{\left( k \right)}{\hat{\boldsymbol{\beta}}}_{\text{MLE}}^{\left( k \right)}=\boldsymbol{\beta}^{\star}+{\overline{\mathbf{V}}}_{\text{EE}}^{-1}\sum_{k=1}^{K} w^{\left( k \right)}\mathbf{D}^{\left( k \right)}\left( \boldsymbol{\beta}^{\star} \right)+o_{\mathbb{P}}\left( n^{-1/2} \right) \\ & =\boldsymbol{\beta}^{\star}+\{\sum_{k=1}^{K} w^{\left( k \right)}\mathbf{V}^{\left( k \right)}\left( {\hat{\boldsymbol{\beta}}}_{\text{MLE}} \right){\}}^{-1}\sum_{k=1}^{K} w^{\left( k \right)}\mathbf{D}^{\left( k \right)}\left( \boldsymbol{\beta}^{\star} \right)+o_{\mathbb{P}}\left( n^{-1/2} \right) \\ & =\boldsymbol{\beta}^{\star}+\{\sum_{k=1}^{K} w^{\left( k \right)}\mathcal{T}_{\boldsymbol{\beta}^{\star}}^{\left( k \right)}+o_{\mathbb{P}}\left( 1 \right){\}}^{-1}\sum_{k=1}^{K} w^{\left( k \right)}\mathbf{D}^{\left( k \right)}\left( \boldsymbol{\beta}^{\star} \right)+o_{\mathbb{P}}\left( n^{-1/2} \right) , \end{matrix}$ |  |
| --- | --- |

where, to obtain the last line, we used the fact that Lemma 7 ensures $\mathbf{V}^{\left( k \right)}\left( {\hat{\boldsymbol{\beta}}}_{\text{MLE}} \right)=\mathcal{T}_{\boldsymbol{\beta}^{\star}}^{\left( k \right)}+o_{\mathbb{P}}\left( 1 \right)$. Rearranging terms and considering $\sqrt{n}\left( {\hat{\boldsymbol{\beta}}}_{\text{EE}}-\boldsymbol{\beta}^{\star} \right)$, the corresponding right-hand side is then asymptotically equivalent to the right-hand side of (18), and one concludes

| $\begin{matrix} \sqrt{n}\left( {\hat{\boldsymbol{\beta}}}_{\text{EE}}-\boldsymbol{\beta}^{\star} \right)\to& \mathcal{N}\left( 0,\boldsymbol{\Sigma}_{\text{Pooled}} \right) . \end{matrix}$ |  |
| --- | --- |

***Theory for the distributed estimation using a single gradient-enhanced log-likelihood***

Let ${\hat{\boldsymbol{\beta}}}_{SL}^{\left( 1 \right)}$ to denote the surrogate likelihood estimator computed at node $k=1$, and recall that ${\hat{\boldsymbol{\beta}}}_{SL}^{\left( 1 \right)}$ satisfies

| $\mathbf{D}^{\left( 1 \right)}\left( {\hat{\boldsymbol{\beta}}}_{SGE,1} \right)+\sum_{k=1}^{K} w^{\left( k \right)}\mathbf{D}^{\left( k \right)}\left( {\hat{\boldsymbol{\beta}}}_{SA} \right)-\mathbf{D}^{\left( 1 \right)}\left( {\hat{\boldsymbol{\beta}}}_{SA} \right)=0 .$ |  |
| --- | --- |

As $\left( \boldsymbol{\beta}^{\star}-{\hat{\boldsymbol{\beta}}}_{\text{SA}} \right)=O_{\mathbb{P}}\left( n^{-1/2} \right)$, and since Lemma 7 guarantees that $\mathbf{V}^{\left( k \right)}\left( {\hat{\boldsymbol{\beta}}}_{\text{SA}} \right)=\mathcal{T}_{\boldsymbol{\beta}^{\star}}^{\left( k \right)}+o_{\mathbb{P}}\left( 1 \right)$, one has from Equation (19) that it holds for each $k\in\{1,\ldots,K\}$ that $\mathbf{D}^{\left( k \right)}\left( {\hat{\boldsymbol{\beta}}}_{\text{SA}} \right)=\mathbf{D}^{\left( k \right)}\left( \boldsymbol{\beta}^{\star} \right)+\mathcal{T}_{\boldsymbol{\beta}^{\star}}^{\left( k \right)}\left( \boldsymbol{\beta}^{\star}-{\hat{\boldsymbol{\beta}}}_{\text{SA}} \right)+o_{\mathbb{P}}\left( n^{-1/2} \right)$. Hence,

| $\mathbf{D}^{\left( 1 \right)}\left( {\hat{\boldsymbol{\beta}}}_{SGE,1} \right)-\mathbf{D}^{\left( 1 \right)}\left( \boldsymbol{\beta}^{\star} \right)+\sum_{k=1}^{K} w^{\left( k \right)}\mathbf{D}^{\left( k \right)}\left( \boldsymbol{\beta}^{\star} \right)+\left( \mathcal{T}_{\boldsymbol{\beta}^{\star}}-\mathcal{T}_{\boldsymbol{\beta}^{\star}}^{\left( 1 \right)} \right)\left( \boldsymbol{\beta}^{\star}-{\hat{\boldsymbol{\beta}}}_{\text{SA}} \right)=o_{\mathbb{P}}\left( n^{-1/2} \right) ,$ | $\left( 21 \right)$ |
| --- | --- |

where one recalls that $\mathcal{T}_{\boldsymbol{\beta}^{\star}}=\sum_{k=1}^{K} w^{\left( k \right)}\mathcal{T}_{\boldsymbol{\beta}^{\star}}^{\left( k \right)}$.

Next, proceeding as in the proof of Lemma 5 one can show that ${\hat{\boldsymbol{\beta}}}_{SGE,1}=\boldsymbol{\beta}^{\star}+o_{\mathbb{P}}\left( 1 \right)$. By Lemma 7, the latter result ensures that $\mathbf{V}^{\left( 1 \right)}\left( {\hat{\boldsymbol{\beta}}}_{\text{SL}} \right)=\mathcal{T}_{\boldsymbol{\beta}^{\star}}^{\left( 1 \right)}+o_{\mathbb{P}}\left( 1 \right)$. In view of this result, combining the multivariate Taylor’s theorem, the equality $\nabla_{\boldsymbol{\beta}}\mathbf{D}^{\left( k \right)}\left( \boldsymbol{\beta} \right)=-\mathbf{V}^{\left( k \right)}\left( \boldsymbol{\beta} \right)$ and the fact that $\mathbf{V}^{\left( 1 \right)}\left( \boldsymbol{\beta}^{\star} \right)=\mathcal{T}_{\boldsymbol{\beta}^{\star}}^{\left( 1 \right)}+o_{\mathbb{P}}\left( 1 \right)$ yields the relationship $\mathbf{D}^{\left( 1 \right)}\left( {\hat{\boldsymbol{\beta}}}_{SGE,1} \right)=\mathbf{D}^{\left( 1 \right)}\left( \boldsymbol{\beta}^{\star} \right)-\{\mathcal{T}_{\boldsymbol{\beta}^{\star}}^{\left( 1 \right)}+o_{\mathbb{P}}\left( 1 \right)\}\left( {\hat{\boldsymbol{\beta}}}_{SL}^{\left( 1 \right)}-\boldsymbol{\beta}^{\star} \right)$ and therefore ${\hat{\boldsymbol{\beta}}}_{SGE,1}-\boldsymbol{\beta}^{\star}=-\{\mathcal{T}_{\boldsymbol{\beta}^{\star}}^{\left( 1 \right)}+o_{\mathbb{P}}\left( 1 \right){\}}^{-1}\left( \mathbf{D}^{\left( 1 \right)}\left( {\hat{\boldsymbol{\beta}}}_{SGE,1} \right)-\mathbf{D}^{\left( 1 \right)}\left( \boldsymbol{\beta}^{\star} \right) \right)$. Moreover, in view of Lemma 6 one has

| $\begin{matrix} {\hat{\boldsymbol{\beta}}}_{\text{SA}}-\boldsymbol{\beta}^{\star}=\sum_{k=1}^{K} w^{\left( k \right)}\left( {\hat{\boldsymbol{\beta}}}_{\text{MLE}}^{\left( k \right)}-\boldsymbol{\beta}^{\star} \right)=\sum_{k=1}^{K} \frac{w^{\left( k \right)}}{\sqrt{p^{\left( k \right)}}}\left( \mathcal{T}_{\boldsymbol{\beta}^{\star}}^{\left( k \right)} \right)^{-1}\mathbf{D}^{\left( k \right)}\left( \boldsymbol{\beta}^{\star} \right)+o_{\mathbb{P}}\left( n^{-1/2} \right) . \end{matrix}$ | $\left( 22 \right)$ |
| --- | --- |

Denoting by $\mathbf{I}_{p+1}$ the $p+1$ square identity matrix, one obtains by combining the derived expression for ${\hat{\boldsymbol{\beta}}}_{SGE,1}-\boldsymbol{\beta}^{\star}$ with (21) and (22) that

| ${\hat{\boldsymbol{\beta}}}_{SL}^{\left( 1 \right)}-\boldsymbol{\beta}^{\star}=\{\mathcal{T}_{\boldsymbol{\beta}^{\star}}^{\left( 1 \right)}+o_{\mathbb{P}}\left( 1 \right){\}}^{-1}\sum_{k=1}^{K} w^{\left( k \right)}\left[ \mathbf{I}_{p+1}+\frac{1}{\sqrt{p^{\left( k \right)}}}\left( \mathcal{T}_{\boldsymbol{\beta}^{\star}}^{\left( 1 \right)}-\mathcal{T}_{\boldsymbol{\beta}^{\star}} \right)\left( \mathcal{T}_{\boldsymbol{\beta}^{\star}}^{\left( k \right)} \right)^{-1} \right]\mathbf{D}^{\left( k \right)}\left( \boldsymbol{\beta}^{\star} \right)+o_{\mathbb{P}}\left( n^{-1/2} \right) .$ |
| --- |

Since the $\mathbf{D}^{\left( k \right)}$’s are $O_{\mathbb{P}}\left( n^{-1/2} \right)$ (see Lemma 2), one deduces that

| ${\hat{\boldsymbol{\beta}}}_{SL}^{\left( 1 \right)}-\boldsymbol{\beta}^{\star}=\left( \mathcal{T}_{\boldsymbol{\beta}^{\star}}^{\left( 1 \right)} \right)^{-1}\sum_{k=1}^{K} w^{\left( k \right)}\left[ \mathbf{I}_{p+1}+\frac{1}{\sqrt{p^{\left( k \right)}}}\left( \mathcal{T}_{\boldsymbol{\beta}^{\star}}^{\left( 1 \right)}-\mathcal{T}_{\boldsymbol{\beta}^{\star}} \right)\left( \mathcal{T}_{\boldsymbol{\beta}^{\star}}^{\left( k \right)} \right)^{-1} \right]\mathbf{D}^{\left( k \right)}\left( \boldsymbol{\beta}^{\star} \right)+o_{\mathbb{P}}\left( n^{-1/2} \right) .$ | $\left( 23 \right)$ |
| --- | --- |

Therefore, $\sqrt{n}\left( {\hat{\boldsymbol{\beta}}}_{SGE,1}-\boldsymbol{\beta}^{\star} \right)$ converges in distribution to a mean 0 multivariate normal random variable with variance-covariance matrix given by

| $\begin{matrix} \Sigma_{\text{SL}}= & \phi^{\star}\left( \mathcal{T}_{\boldsymbol{\beta}^{\star}}^{\left( 1 \right)} \right)^{-1}\left\{ \sum_{k=1}^{K} \frac{{w^{\left( k \right)}}^{2}}{p^{\left( k \right)}}\left( \mathbf{A}_{\boldsymbol{\beta}^{\star}}^{\left( k \right)} \right)^{\top}\mathcal{T}_{\boldsymbol{\beta}^{\star}}^{\left( k \right)}\mathbf{A}_{\boldsymbol{\beta}^{\star}}^{\left( k \right)} \right\}\left( \mathcal{T}_{\boldsymbol{\beta}^{\star}}^{\left( 1 \right)} \right)^{-1} \\ & \text{where} \mathbf{A}_{\boldsymbol{\beta}^{\star}}^{\left( k \right)}=\sqrt{p^{\left( k \right)}}\mathbf{I}_{p+1}+\left( \mathcal{T}_{\boldsymbol{\beta}^{\star}}^{\left( 1 \right)}-\mathcal{T}_{\boldsymbol{\beta}^{\star}} \right)\left( \mathcal{T}_{\boldsymbol{\beta}^{\star}}^{\left( k \right)} \right)^{-1} . \end{matrix}$ |  |
| --- | --- |

If two iterations are executed, one first uses the fact that

| $\mathbf{D}^{\left( 1 \right)}\left( {\hat{\boldsymbol{\beta}}}_{SGE,2} \right)-\mathbf{D}^{\left( 1 \right)}\left( \boldsymbol{\beta}^{\star} \right)+\sum_{k=1}^{K} w^{\left( k \right)}\mathbf{D}^{\left( k \right)}\left( \boldsymbol{\beta}^{\star} \right)+\left( \mathcal{T}_{\boldsymbol{\beta}^{\star}}-\mathcal{T}_{\boldsymbol{\beta}^{\star}}^{\left( 1 \right)} \right)\left( \boldsymbol{\beta}^{\star}-{\hat{\boldsymbol{\beta}}}_{SGE,1} \right)=o_{\mathbb{P}}\left( n^{-1/2} \right) .$ | $\left( 24 \right)$ |
| --- | --- |

From the last equation, an application of the multivariate Taylor expansion combined with Lemma 2 and Lemma 7 ensures ${\hat{\boldsymbol{\beta}}}_{SGE,2}=\boldsymbol{\beta}^{\star}+O_{\mathbb{P}}\left( n^{-1/2} \right)$. Hence, one obtains that

| $\begin{matrix} & {\hat{\boldsymbol{\beta}}}_{SGE,2}-\boldsymbol{\beta}^{\star} \\ & =(\mathcal{T}_{\boldsymbol{\beta}^{\star}}^{\left( 1 \right)})^{-1}\left( \mathcal{T}_{\boldsymbol{\beta}^{\star}}^{\left( 1 \right)}-\mathcal{T}_{\boldsymbol{\beta}^{\star}} \right)\left( {\hat{\boldsymbol{\beta}}}_{SGE,1}-\boldsymbol{\beta}^{\star} \right)+(\mathcal{T}_{\boldsymbol{\beta}^{\star}}^{\left( 1 \right)})^{-1}\sum_{k=1}^{K} w^{\left( k \right)}\mathbf{D}^{\left( k \right)}\left( \boldsymbol{\beta}^{\star} \right)+o_{\mathbb{P}}\left( n^{-1/2} \right) . \end{matrix}$ | $\left( 25 \right)$ |
| --- | --- |

With $\mathbf{S}=\left( \mathcal{T}_{\boldsymbol{\beta}^{\star}}^{\left( 1 \right)} \right)^{-1}$ and $\mathbf{U}=\mathcal{T}_{\boldsymbol{\beta}^{\star}}^{\left( 1 \right)}-\mathcal{T}_{\boldsymbol{\beta}^{\star}}$, the last equation expresses as

| $\begin{matrix} {\hat{\boldsymbol{\beta}}}_{SGE,2}-\boldsymbol{\beta}^{\star}=\mathbf{SU}\left( {\hat{\boldsymbol{\beta}}}_{SGE,1}-\boldsymbol{\beta}^{\star} \right)+\mathbf{S}\sum_{k=1}^{K} w^{\left( k \right)}\mathbf{D}^{\left( k \right)}\left( \boldsymbol{\beta}^{\star} \right)+o_{\mathbb{P}}\left( n^{-1/2} \right) . \end{matrix}$ |  |
| --- | --- |

Since from (23) one has

| ${\hat{\boldsymbol{\beta}}}_{SGE,1}-\boldsymbol{\beta}^{\star}=\sum_{k=1}^{K} w^{\left( k \right)}\left[ \mathbf{S}+\frac{1}{\sqrt{p^{\left( k \right)}}}\mathbf{SUS} \right]\mathbf{D}^{\left( k \right)}\left( \boldsymbol{\beta}^{\star} \right)+o_{\mathbb{P}}\left( n^{-1/2} \right) .$ |  |
| --- | --- |

it follows that

| $\begin{matrix} {\hat{\boldsymbol{\beta}}}_{SGE,2}-\boldsymbol{\beta}^{\star}=\sum_{k=1}^{K} w^{\left( k \right)}\left[ \mathbf{S}+\mathbf{SUS}+\frac{1}{\sqrt{p^{\left( k \right)}}}\left( \mathbf{SU} \right)^{2}\mathbf{S} \right]\mathbf{D}^{\left( k \right)}\left( \boldsymbol{\beta}^{\star} \right)+o_{\mathbb{P}}\left( n^{-1/2} \right) . \end{matrix}$ |  |
| --- | --- |

Hence, in general, the asymptotic distribution of ${\hat{\boldsymbol{\beta}}}_{SGE,2}-\boldsymbol{\beta}^{\star}$ does not match that of ${\hat{\boldsymbol{\beta}}}_{SGE,1}-\boldsymbol{\beta}^{\star}$ in (23).

***Theory for the distributed estimation using multiple gradient-enhanced log-likelihoods***

Let ${\hat{\boldsymbol{\beta}}}_{MGE,1}^{\left( k \right)}$ denote the surrogate likelihood estimator computed at node $k$. Proceeding as we did in the last section to derive (23), one can show that it holds for all $k\in\{1,\ldots,K\}$ that as $n\to\infty$,

| $\begin{matrix} {\hat{\boldsymbol{\beta}}}_{MGE,1}^{\left( k \right)}-\boldsymbol{\beta}^{\star} & =\left( \mathcal{T}_{\boldsymbol{\beta}^{\star}}^{\left( k \right)} \right)^{-1}\sum_{k'=1}^{K} w^{\left( k' \right)}\left[ \mathbf{I}_{p+1}+\frac{1}{\sqrt{p^{\left( k' \right)}}}\left( \mathcal{T}_{\boldsymbol{\beta}^{\star}}^{\left( k \right)}-\mathcal{T}_{\boldsymbol{\beta}^{\star}} \right)\left( \mathcal{T}_{\boldsymbol{\beta}^{\star}}^{\left( k' \right)} \right)^{-1} \right]\mathbf{D}^{\left( k' \right)}\left( \boldsymbol{\beta}^{\star} \right)+o_{\mathbb{P}}\left( n^{-1/2} \right) \\ & =\sum_{k'=1}^{K} w^{\left( k' \right)}\left[ \left( \mathcal{T}_{\boldsymbol{\beta}^{\star}}^{\left( k \right)} \right)^{-1}+\frac{1}{\sqrt{p^{\left( k' \right)}}}\left( \mathbf{I}_{p+1}-\left( \mathcal{T}_{\boldsymbol{\beta}^{\star}}^{\left( k \right)} \right)^{-1}\mathcal{T}_{\boldsymbol{\beta}^{\star}} \right)\left( \mathcal{T}_{\boldsymbol{\beta}^{\star}}^{\left( k' \right)} \right)^{-1} \right]\mathbf{D}^{\left( k' \right)}\left( \boldsymbol{\beta}^{\star} \right)+o_{\mathbb{P}}\left( n^{-1/2} \right) \end{matrix}$ |
| --- |

Therefore, letting $\mathcal{U}_{\boldsymbol{\beta}^{\star}}=\sum_{k=1}^{K} w^{\left( k \right)}\left( \mathcal{T}_{\boldsymbol{\beta}^{\star}}^{\left( k \right)} \right)^{-1}$, one obtains that

| $\begin{matrix} & {\hat{\boldsymbol{\beta}}}_{MGE,1}-\boldsymbol{\beta}^{\star}=\sum_{k=1}^{K} w^{\left( k \right)}\left( {\hat{\boldsymbol{\beta}}}_{MGE,1}^{\left( k \right)}-\boldsymbol{\beta}^{\star} \right) \\ & =\sum_{k=1}^{K} w^{\left( k \right)}\left[ \mathcal{U}_{\boldsymbol{\beta}^{\star}}+\frac{1}{\sqrt{p^{\left( k \right)}}}\left( \mathbf{I}_{p+1}-\mathcal{U}_{\boldsymbol{\beta}^{\star}}\mathcal{T}_{\boldsymbol{\beta}^{\star}} \right)\left( \mathcal{T}_{\boldsymbol{\beta}^{\star}}^{\left( k \right)} \right)^{-1} \right]\mathbf{D}^{\left( k \right)}\left( \boldsymbol{\beta}^{\star} \right)+o_{\mathbb{P}}\left( n^{-1/2} \right) . \end{matrix}$ |
| --- |

Therefore, $\sqrt{n}\left( {\hat{\boldsymbol{\beta}}}_{MGE,1}-\boldsymbol{\beta}^{\star} \right)$ converges in distribution to a mean 0 multivariate normal random variable with variance-covariance matrix given by

| $\begin{matrix} \boldsymbol{\Sigma}_{\text{MGE,1}}= & \phi^{\star}\sum_{k=1}^{K} \frac{{w^{\left( k \right)}}^{2}}{p^{\left( k \right)}}\left[ \sqrt{p^{\left( k \right)}}\mathcal{U}_{\boldsymbol{\beta}^{\star}}\mathcal{T}_{\boldsymbol{\beta}^{\star}}^{\left( k \right)}+\left( \mathbf{I}_{p+1}-\mathcal{U}_{\boldsymbol{\beta}^{\star}}\mathcal{T}_{\boldsymbol{\beta}^{\star}} \right) \right] \\ & \times\left[ \sqrt{p^{\left( k \right)}}\mathcal{U}_{\boldsymbol{\beta}^{\star}}+\left( \mathbf{I}_{p+1}-\mathcal{U}_{\boldsymbol{\beta}^{\star}}\mathcal{T}_{\boldsymbol{\beta}^{\star}} \right)\left( \mathcal{T}_{\boldsymbol{\beta}^{\star}}^{\left( k \right)} \right)^{-1} \right] . \end{matrix}$ |  |
| --- | --- |

**Auxiliary results**

The following lemma transfers the conditions on the marginal moments imposed in (C3) into a condition on $E\left\{ \|\mathbf{x}{\|}_{1}^{4} \right\}$ that is used in the proof of Lemma 2.

**Lemma 1**. *Denote by* $\mathbf{x}$ *a* $p+1$ *dimensional random vector such that* $E\left\{ \left[ |\mathbf{x}| \right]_{j}^{6} \right\}<\infty$ *for all* $1\leq j\leq p+1$*. Then* $E\left\{ \|\mathbf{x}{\|}_{1}^{4} \right\}<\infty$*.*

*Proof.* Note first that for a multiindex $\boldsymbol{\alpha}\in\mathbb{N}^{p+1}$ such that $\|\boldsymbol{\alpha}{\|}_{1}=4$ we have essentially $5$ possibilities for $\boldsymbol{\alpha}$: There is one non-zero element $\left[ \boldsymbol{\alpha} \right]_{j_{1}}=4$ at position $j_{1}$, there are two non-zero elements $\left[ \boldsymbol{\alpha} \right]_{j_{1}}$ and $\left[ \boldsymbol{\alpha} \right]_{j_{2}}$, $j_{1}\neq j_{2}$, in $\boldsymbol{\alpha}$ where we either have $\left[ \boldsymbol{\alpha} \right]_{j_{1}}=3$ and $\left[ \boldsymbol{\alpha} \right]_{j_{2}}=1$ or $\left[ \boldsymbol{\alpha} \right]_{j_{1}}=\left[ \boldsymbol{\alpha} \right]_{j_{2}}=2$, there are three non-zero elements $\left[ \boldsymbol{\alpha} \right]_{j_{1}}=2$, $\left[ \boldsymbol{\alpha} \right]_{j_{2}}=\left[ \boldsymbol{\alpha} \right]_{j_{3}}=1$, $j_{1}\neq j_{2}\neq j_{3}$, in $\boldsymbol{\alpha}$, and lastly there are four non-zero elements $\left[ \boldsymbol{\alpha} \right]_{j_{1}}=\left[ \boldsymbol{\alpha} \right]_{j_{2}}=\left[ \boldsymbol{\alpha} \right]_{j_{3}}=\left[ \boldsymbol{\alpha} \right]_{j_{4}}=1$ for $j_{1}\neq j_{2}\neq j_{3}\neq j_{4}$. Concerning the expectation of $|\mathbf{x}|^{\boldsymbol{\alpha}}$ for a random vector $\mathbf{x}$ we have by applying the (generalized) Hölder inequality for the five cases that

| $\begin{matrix} E\left\{ \vert\mathbf{x}\vert^{\boldsymbol{\alpha}} \right\}=\left\{ \begin{matrix} E\left\{ \left[ \vert\mathbf{x}\vert\right]_{j_{1}}^{4} \right\}, \\ E\left\{ \left[ \vert\mathbf{x}\vert\right]_{j_{1}}^{3}\left[ \vert\mathbf{x}\vert\right]_{j_{2}} \right\}\leq\sqrt{E\left\{ \left[ \vert\mathbf{x}\vert\right]_{j_{1}}^{6} \right\}}\sqrt{E\left\{ \left[ \vert\mathbf{x}\vert\right]_{j_{2}}^{2} \right\}}, \\ E\left\{ \left[ \vert\mathbf{x}\vert\right]_{j_{1}}^{2}\left[ \vert\mathbf{x}\vert\right]_{j_{2}}^{2} \right\}\leq\sqrt{E\left\{ \left[ \vert\mathbf{x}\vert\right]_{j_{1}}^{4} \right\}}\sqrt{E\left\{ \left[ \vert\mathbf{x}\vert\right]_{j_{2}}^{4} \right\}}, \\ E\left\{ \left[ \vert\mathbf{x}\vert\right]_{j_{1}}^{2}\left[ \vert\mathbf{x}\vert\right]_{j_{2}}\left[ \vert\mathbf{x}\vert\right]_{j_{3}} \right\}\leq\sqrt[3]{E\left\{ \left[ \vert\mathbf{x}\vert\right]_{j_{1}}^{6} \right\}}\sqrt[3]{E\left\{ \left[ \vert\mathbf{x}\vert\right]_{j_{2}}^{3} \right\}}\sqrt[3]{E\left\{ \left[ \vert\mathbf{x}\vert\right]_{j_{3}}^{3} \right\}}, \\ E\left\{ \left[ \vert\mathbf{x}\vert\right]_{j_{1}}\left[ \vert\mathbf{x}\vert\right]_{j_{2}}\left[ \vert\mathbf{x}\vert\right]_{j_{3}}\left[ \vert\mathbf{x}\vert\right]_{j_{4}} \right\}\leq\sqrt[4]{E\left\{ \left[ \vert\mathbf{x}\vert\right]_{j_{1}}^{4} \right\}}\sqrt[4]{E\left\{ \left[ \vert\mathbf{x}\vert\right]_{j_{2}}^{4} \right\}}\sqrt[4]{E\left\{ \left[ \vert\mathbf{x}\vert\right]_{j_{3}}^{4} \right\}}\sqrt[4]{E\left\{ \left[ \vert\mathbf{x}\vert\right]_{j_{4}}^{4} \right\}}. \end{matrix} \right. \end{matrix}$ |
| --- |

Given that $E\left\{ \left[ |\mathbf{x}| \right]_{j}^{6} \right\}<\infty$ implies also $E\left\{ \left[ |\mathbf{x}| \right]_{j}^{\mathcal{l}} \right\}<\infty$ for $1\mathcal{\leq l\leq}5$, we see that $E\left\{ |\mathbf{x}|^{\boldsymbol{\alpha}} \right\}<\infty$ for every multiindex $\boldsymbol{\alpha}$ with $\|\boldsymbol{\alpha}{\|}_{1}=4$. Applying the multinomial theorem to $\|\mathbf{x}{\|}_{1}^{4}$ now shows that

| $\begin{matrix} E\left\{ \Vert\mathbf{x}{\Vert}_{1}^{4} \right\}=E\left\{ \sum_{\Vert\boldsymbol{\alpha}{\Vert}_{1}=4} \binom{4}{\boldsymbol{\alpha}}\vert\mathbf{x}\vert^{\boldsymbol{\alpha}} \right\}=\sum_{\Vert\boldsymbol{\alpha}{\Vert}_{1}=4} \binom{4}{\boldsymbol{\alpha}}E\left\{ \vert\mathbf{x}\vert^{\boldsymbol{\alpha}} \right\}<\infty. \end{matrix}$ |  |
| --- | --- |

 ◻

**Lemma 2**. *Under Conditions (C2)–(C5), it holds that*

| $\sup_{\boldsymbol{\beta}\in\Theta}\vert\sqrt{n^{\left( k \right)}}[\mathbf{D}^{\left( k \right)}\left( \boldsymbol{\beta} \right)-E\{\mathbf{D}^{\left( k \right)}\left( \boldsymbol{\beta} \right)\}]=O_{\mathbb{P}}\left( 1 \right)$ | $(26)$ |
| --- | --- |

*for all* $k\in\{1,\ldots,K\}$*.*

*Proof.* Let $\boldsymbol{\psi}_{i}^{\left( k \right)}\left( \boldsymbol{\beta} \right)\in\mathbb{R}^{p+1}$ such that $\boldsymbol{\psi}_{i}^{\left( k \right)}\left( \boldsymbol{\beta} \right)=\mathbf{x}_{i}^{\left( k \right)}h'\left( \boldsymbol{\beta}^{\top}\mathbf{x}_{i}^{\left( k \right)} \right)\left( y_{i}^{\left( k \right)}-b'\{h\left( \boldsymbol{\beta}^{\top}\mathbf{x}_{i}^{\left( k \right)} \right)\} \right)$. In this notation we have $\left( n^{\left( k \right)} \right)^{-1}\sum_{i=1}^{n^{\left( k \right)}} \boldsymbol{\psi}_{i}^{\left( k \right)}\left( \boldsymbol{\beta} \right)=\mathbf{D}^{\left( k \right)}\left( \boldsymbol{\beta} \right)$. For any $\boldsymbol{\beta}_{1},\boldsymbol{\beta}_{2}\in\Theta$ one has

| $\begin{matrix} \boldsymbol{\psi}_{i}^{\left( k \right)}\left( \boldsymbol{\beta}_{1} \right)-\boldsymbol{\psi}_{i}^{\left( k \right)}\left( \boldsymbol{\beta}_{2} \right)= & \mathbf{x}_{i}^{\left( k \right)} \left\{ h'\left( \boldsymbol{\beta}_{1}^{\top}\mathbf{x}_{i}^{\left( k \right)} \right)-h'\left( \boldsymbol{\beta}_{2}^{\top}\mathbf{x}_{i}^{\left( k \right)} \right) \right\}\left( y_{i}^{\left( k \right)}-b'\{h\left( \boldsymbol{\beta}_{1}^{\top}\mathbf{x}_{i}^{\left( k \right)} \right)\} \right) \\ & +\mathbf{x}_{i}^{\left( k \right)}h'\left( \boldsymbol{\beta}_{2}^{\top}\mathbf{x}_{i}^{\left( k \right)} \right)\left( b'\{h\left( \boldsymbol{\beta}_{2}^{\top}\mathbf{x}_{i}^{\left( k \right)} \right)\}-b'\{h\left( \boldsymbol{\beta}_{1}^{\top}\mathbf{x}_{i}^{\left( k \right)} \right)\} \right) . \end{matrix}$ |
| --- |

Hence,

| $\begin{matrix} \left\Vert\boldsymbol{\psi}_{i}^{\left( k \right)}\left( \boldsymbol{\beta}_{1} \right)-\boldsymbol{\psi}_{i}^{\left( k \right)}\left( \boldsymbol{\beta}_{2} \right) \right\Vert_{\infty}\leq& \Vert\mathbf{x}_{i}^{\left( k \right)}{\Vert}_{\infty} \left\vert h'\left( \boldsymbol{\beta}_{1}^{\top}\mathbf{x}_{i}^{\left( k \right)} \right)-h'\left( \boldsymbol{\beta}_{2}^{\top}\mathbf{x}_{i}^{\left( k \right)} \right) \right\vert\left[ \vert y_{i}^{\left( k \right)}\vert+\vert b'\{h\left( \boldsymbol{\beta}_{1}^{\top}\mathbf{x}_{i}^{\left( k \right)} \right)\}\vert\right] \\ & +\Vert\mathbf{x}_{i}^{\left( k \right)}{\Vert}_{\infty}\vert h'\left( \boldsymbol{\beta}_{2}^{\top}\mathbf{x}_{i}^{\left( k \right)} \right)\vert\left\vert b'\{h\left( \boldsymbol{\beta}_{2}^{\top}\mathbf{x}_{i}^{\left( k \right)} \right)\}-b'\{h\left( \boldsymbol{\beta}_{1}^{\top}\mathbf{x}_{i}^{\left( k \right)} \right)\} \right\vert. \end{matrix}$ |
| --- |

Since by Condition (C2) $h'$ is differentiable, then, recalling the definition of $\Upsilon_{\mathcal{l}}$ in Condition (C5), one deduces from the mean-value theorem and the dual version of the Cauchy–Schwarz inequality $|\mathbf{y}^{\top}\mathbf{x}|\leq\|\mathbf{y}{\|}_{\infty}\|\mathbf{x}{\|}_{1}$ that

| $\begin{matrix} \vert h'\left( \boldsymbol{\beta}_{1}^{\top}\mathbf{x}_{i}^{\left( k \right)} \right)-h'\left( \boldsymbol{\beta}_{2}^{\top}\mathbf{x}_{i}^{\left( k \right)} \right)\vert& \leq\Upsilon_{2}\left( \mathbf{x}_{i}^{\left( k \right)} \right)\parallel\boldsymbol{\beta}_{1}-\boldsymbol{\beta}_{2}\parallel_{\infty}\parallel\mathbf{x}_{i}^{\left( k \right)}\parallel_{1} . \end{matrix}$ |  |
| --- | --- |

Recalling the definition of $\tilde{\Upsilon}_{\mathcal{l}}$ in Condition (C5) one similarly has

| $\begin{matrix} \vert b'\{h\left( \boldsymbol{\beta}_{2}^{\top}\mathbf{x}_{i}^{\left( k \right)} \right)\}-b'\{h\left( \boldsymbol{\beta}_{1}^{\top}\mathbf{x}_{i}^{\left( k \right)} \right)\vert\leq\tilde{\Upsilon}_{1}\left( \mathbf{x}_{i}^{\left( k \right)} \right)\parallel\boldsymbol{\beta}_{1}-\boldsymbol{\beta}_{2}\parallel_{\infty}\parallel\mathbf{x}_{i}^{\left( k \right)}\parallel_{1} . \end{matrix}$ |  |
| --- | --- |

As one also has $|h'\left( \boldsymbol{\beta}_{2}^{\top}\mathbf{x}_{i}^{\left( k \right)} \right)|\leq\Upsilon_{1}\left( \mathbf{x}_{i}^{\left( k \right)} \right)$ and $|b'\{h\left( \boldsymbol{\beta}_{1}^{\top}\mathbf{x}_{i}^{\left( k \right)} \right)\}|\leq\tilde{\Upsilon}_{0}\left( \mathbf{x}_{i}^{\left( k \right)} \right)$, the above equations imply with $\|\mathbf{x}_{i}^{\left( k \right)}{\|}_{\infty}\leq\|\mathbf{x}_{i}^{\left( k \right)}{\|}_{1}$ that

| $\begin{matrix} \left\Vert\boldsymbol{\psi}_{i}^{\left( k \right)}\left( \boldsymbol{\beta}_{1} \right)-\boldsymbol{\psi}_{i}^{\left( k \right)}\left( \boldsymbol{\beta}_{2} \right) \right\Vert_{\infty} & \leq\parallel\boldsymbol{\beta}_{1}-\boldsymbol{\beta}_{2}\parallel_{\infty}m\left( y_{i}^{\left( k \right)},\mathbf{x}_{i}^{\left( k \right)} \right) , \end{matrix}$ |  |
| --- | --- |

where we set

| $\begin{matrix} m\left( y_{i}^{\left( k \right)},\mathbf{x}_{i}^{\left( k \right)} \right)=\Vert\mathbf{x}_{i}^{\left( k \right)}{\Vert}_{1}^{2}\left( \Upsilon_{2}\left( \mathbf{x}_{i}^{\left( k \right)} \right)\left( \vert y_{i}^{\left( k \right)}\vert+\tilde{\Upsilon}_{0}\left( \mathbf{x}_{i}^{\left( k \right)} \right) \right)+\Upsilon_{1}\left( \mathbf{x}_{i}^{\left( k \right)} \right)\tilde{\Upsilon}_{1}\left( \mathbf{x}_{i}^{\left( k \right)} \right) \right). \end{matrix}$ |  |
| --- | --- |

Using first the Hölder and then twice the Minkowski (triangle) inequality we now have

| $\begin{matrix} E\left\{ m\left( y_{i}^{\left( k \right)},\mathbf{x}_{i}^{\left( k \right)} \right) \right\} & \leq\sqrt{E\left\{ \Vert\mathbf{x}_{i}^{\left( k \right)}{\Vert}_{1}^{4} \right\}}\sqrt{E\left\{ \left( \Upsilon_{2}\left( \mathbf{x}_{i}^{\left( k \right)} \right)\left( \vert y_{i}^{\left( k \right)}\vert+\tilde{\Upsilon}_{0}\left( \mathbf{x}_{i}^{\left( k \right)} \right) \right)+\Upsilon_{1}\left( \mathbf{x}_{i}^{\left( k \right)} \right)\tilde{\Upsilon}_{1}\left( \mathbf{x}_{i}^{\left( k \right)} \right) \right)^{2} \right\}} \\ & \leq\sqrt{E\left\{ \Vert\mathbf{x}_{i}^{\left( k \right)}{\Vert}_{1}^{4} \right\}}\left( \sqrt{E\left\{ \left( \Upsilon_{2}\left( \mathbf{x}_{i}^{\left( k \right)} \right)\left( \vert y_{i}^{\left( k \right)}\vert+\tilde{\Upsilon}_{0}\left( \mathbf{x}_{i}^{\left( k \right)} \right) \right) \right)^{2} \right\}} \right. \\ & \left. +\sqrt{E\left\{ \left( \Upsilon_{1}\left( \mathbf{x}_{i}^{\left( k \right)} \right)\tilde{\Upsilon}_{1}\left( \mathbf{x}_{i}^{\left( k \right)} \right) \right)^{2} \right\}} \right) \\ & \leq\sqrt{E\left\{ \Vert\mathbf{x}_{i}^{\left( k \right)}{\Vert}_{1}^{4} \right\}}\left( \sqrt{E\left\{ \left( \Upsilon_{2}\left( \mathbf{x}_{i}^{\left( k \right)} \right)\vert y_{i}^{\left( k \right)}\vert\right)^{2} \right\}} \right. \\ & \left. +\sqrt{E\left\{ \left( \Upsilon_{2}\left( \mathbf{x}_{i}^{\left( k \right)} \right)\tilde{\Upsilon}_{0}\left( \mathbf{x}_{i}^{\left( k \right)} \right) \right)^{2} \right\}}+\sqrt{E\left\{ \left( \Upsilon_{1}\left( \mathbf{x}_{i}^{\left( k \right)} \right)\tilde{\Upsilon}_{1}\left( \mathbf{x}_{i}^{\left( k \right)} \right) \right)^{2} \right\}} \right). \end{matrix}$ |
| --- |

Concerning the individual terms in the parentheses we have by the (generalized) Hölder’s inequality with $1/2=1/4+1/4$ that

| $\begin{matrix} \sqrt{E\left\{ \left( \Upsilon_{2}\left( \mathbf{x}_{i}^{\left( k \right)} \right)\vert y_{i}^{\left( k \right)}\vert\right)^{2} \right\}} & \leq\sqrt[4]{E\left\{ \left( \Upsilon_{2}\left( \mathbf{x}_{i}^{\left( k \right)} \right) \right)^{4} \right\}}\sqrt[4]{E\left\{ \left( \vert y_{i}^{\left( k \right)}\vert\right)^{4} \right\}}, \\ \sqrt{E\left\{ \left( \Upsilon_{2}\left( \mathbf{x}_{i}^{\left( k \right)} \right)\tilde{\Upsilon}_{0}\left( \mathbf{x}_{i}^{\left( k \right)} \right) \right)^{2} \right\}} & \leq\sqrt[4]{E\left\{ \left( \Upsilon_{2}\left( \mathbf{x}_{i}^{\left( k \right)} \right) \right)^{4} \right\}}\sqrt[4]{E\left\{ \left( \tilde{\Upsilon}_{0}\left( \mathbf{x}_{i}^{\left( k \right)} \right) \right)^{4} \right\}}, \\ \sqrt{E\left\{ \left( \Upsilon_{1}\left( \mathbf{x}_{i}^{\left( k \right)} \right)\tilde{\Upsilon}_{1}\left( \mathbf{x}_{i}^{\left( k \right)} \right) \right)^{2} \right\}} & \leq\sqrt[4]{E\left\{ \left( \Upsilon_{1}\left( \mathbf{x}_{i}^{\left( k \right)} \right) \right)^{4} \right\}}\sqrt[4]{E\left\{ \left( \tilde{\Upsilon}_{1}\left( \mathbf{x}_{i}^{\left( k \right)} \right) \right)^{4} \right\}}. \end{matrix}$ |  |
| --- | --- |

Given these estimates we then have

| $\begin{matrix} E\left\{ m\left( y_{i}^{\left( k \right)},\mathbf{x}_{i}^{\left( k \right)} \right) \right\} & \leq\sqrt{E\left\{ \Vert\mathbf{x}_{i}^{\left( k \right)}{\Vert}_{1}^{4} \right\}}\left( \sqrt[4]{E\left\{ \Upsilon_{2}^{4}\left( \mathbf{x}_{i}^{\left( k \right)} \right) \right\}}\left( \sqrt[4]{E\left\{ \vert y_{i}^{\left( k \right)}\vert^{4} \right\}}+\sqrt[4]{E\left\{ \tilde{\Upsilon}_{0}^{4}\left( \mathbf{x}_{i}^{\left( k \right)} \right) \right\}} \right) \right. \\ & \left. +\sqrt[4]{E\left\{ \Upsilon_{1}^{4}\left( \mathbf{x}_{i}^{\left( k \right)} \right) \right\}}\sqrt[4]{E\left\{ \tilde{\Upsilon}_{1}^{4}\left( \mathbf{x}_{i}^{\left( k \right)} \right) \right\}} \right). \end{matrix}$ |
| --- |

In view of the last equation, Condition (C5) in combination with Condition (C3) and Lemma 1 ensures $E\{m\left( y_{i}^{\left( k \right)},\mathbf{x}_{i}^{\left( k \right)} \right)\}=O\left( 1 \right)$. Given that the arguments so far are build on the $\|\cdot{\|}_{\infty}$ norm, conditions (C3) and (C4) now show that each component

| $\begin{matrix} {}_{j}=\left[ \mathbf{x}_{i}^{\left( k \right)} \right]_{j}h'\left( \boldsymbol{\beta}^{\top}\mathbf{x}_{i}^{\left( k \right)} \right)\left( y_{i}^{\left( k \right)}-b'\{h\left( \boldsymbol{\beta}^{\top}\mathbf{x}_{i}^{\left( k \right)} \right)\} \right) \end{matrix}$ |  |
| --- | --- |

of $\boldsymbol{\psi}_{i}^{\left( k \right)}\left( \boldsymbol{\beta} \right)$ has the property

| $\begin{matrix} E\left\{ \vert\left[ \boldsymbol{\psi}_{i}^{\left( k \right)}\left( \boldsymbol{\beta}_{\mathbf{1}} \right) \right]_{j}-\left[ \boldsymbol{\psi}_{i}^{\left( k \right)}\left( \boldsymbol{\beta}_{\mathbf{2}} \right) \right]_{j}\vert\right\}\leq E\left\{ m\left( y_{i}^{\left( k \right)},\mathbf{x}_{i}^{\left( k \right)} \right) \right\}<\infty. \end{matrix}$ |  |
| --- | --- |

This allows to apply Theorem 19.5 in [60] (see example 19.7) to conclude that each component in bounded in probability. Combining this with [61], Lemma 1.4.3 shows that the same is true when considering all components in $\boldsymbol{\psi}_{i}^{\left( k \right)}\left( \boldsymbol{\beta} \right)$ simultaneously. This finally shows that (26) holds. ◻

**Lemma 3**. *Under Conditions (C2)–C5), it holds as* $n\to\infty$ *that* $\sqrt{n^{\left( k \right)}}\mathbf{D}^{\left( k \right)}\left( \boldsymbol{\beta}^{\boldsymbol{\star}} \right)$ *converges in distribution to a centred normal random variable with covariance matrix* $\phi^{\star}\mathcal{T}_{\boldsymbol{\beta}^{\star}}^{\left( k \right)}$*.*

*Proof.* To prove the Lemma we use the Cramér-Wold device. That is, we show that, for any constant $\mathbf{a}\in\mathbb{R}^{p+1}$, the random variable $\sqrt{n^{\left( k \right)}}\mathbf{a}^{\top}\mathbf{D}^{\left( k \right)}\left( \boldsymbol{\beta}^{\boldsymbol{\star}} \right)$ converges in distribution to a centred normal random variable, with variance $\phi^{\star}\mathbf{a}^{\top}\mathcal{T}_{\boldsymbol{\beta}^{\star}}^{\left( k \right)}\mathbf{a}$. To do this, first note that as $E\left( y_{i}^{\left( k \right)}\mid\mathbf{x}_{i}^{\left( k \right)} \right)=b'[h\{\left( \boldsymbol{\beta}^{\star} \right)^{\top}\mathbf{x}_{i}^{\left( k \right)}\}]$, we have $E\{\mathbf{a}^{\top}\mathbf{D}^{\left( k \right)}\left( \boldsymbol{\beta}^{\star} \right)\}=0$ and

| $\begin{matrix} \mathrm{var}\left\{ \sqrt{n^{\left( k \right)}}\mathbf{a}^{\top}\mathbf{D}^{\left( k \right)}\left( \boldsymbol{\beta}^{\star} \right) \right\} & =\frac{1}{n^{\left( k \right)}}\sum_{i=1}^{n^{\left( k \right)}} E\left\{ (\mathbf{a}^{\top}\mathbf{x}_{i}^{\left( k \right)})^{2} h'\left( \boldsymbol{\beta}^{\top}\mathbf{x}_{i}^{\left( k \right)} \right)^{2}\mathrm{var}\left( y_{i}^{\left( k \right)}\mid\mathbf{x}_{i}^{\left( k \right)} \right) \right\} \\ & =\phi^{\star}E\left\{ (\mathbf{a}^{\top}\mathbf{x}_{1}^{\left( k \right)})^{2} h'\left( \boldsymbol{\beta}^{\top}\mathbf{x}_{1}^{\left( k \right)} \right)^{2}b''[h\{\left( \boldsymbol{\beta}^{\star} \right)^{\top}\mathbf{x}_{1}^{\left( k \right)}\}] \right\} \\ & =\phi^{\star}\mathbf{a}^{\top}\left[ E\left\{ \mathbf{x}_{1}^{\left( k \right)}(\mathbf{x}_{1}^{\left( k \right)})^{\top} h'\left( \boldsymbol{\beta}^{\top}\mathbf{x}_{1}^{\left( k \right)} \right)^{2}b''[h\{\left( \boldsymbol{\beta}^{\star} \right)^{\top}\mathbf{x}_{1}^{\left( k \right)}\}] \right\} \right]\mathbf{a} \\ & =\phi^{\star}\mathbf{a}^{\top}\mathcal{T}_{\boldsymbol{\beta}^{\star}}^{\left( k \right)}\mathbf{a} . \end{matrix}$ |
| --- |

To obtain the second line, we used the fact that $\mathrm{var}\left( y_{i}^{\left( k \right)}\mid\mathbf{x}_{i}^{\left( k \right)} \right)=\phi^{\star}b''[h\{\left( \boldsymbol{\beta}^{\star} \right)^{\top}\mathbf{x}_{i}^{\left( k \right)}\}]$ and the assumption that the $\mathbf{x}_{i}^{\left( k \right)}$’s are i.i.d. for a given $k$. For the third line, we used the equality $\left( \mathbf{a}^{\top}\mathbf{x}_{1}^{\left( k \right)} \right)^{2}=\mathbf{a}^{\top}\mathbf{x}_{1}^{\left( k \right)}\left( \mathbf{x}_{1}^{\left( k \right)} \right)^{\top}\mathbf{a}$.

As the $\left( y_{i}^{\left( k \right)},\mathbf{x}_{i}^{\left( k \right)} \right)$’s are i.i.d. random variables, it follows $\sqrt{n^{\left( k \right)}}\mathbf{a}^{\top}\mathbf{D}^{\left( k \right)}\left( \boldsymbol{\beta}^{\star} \right)$ is itself a sum of i.i.d. random variables, with mean $0$ and finite (constant) variance. Therefore, an application of the Lindeberg-Lévy central limit theorem ensures that $\sqrt{n^{\left( k \right)}}\mathbf{a}^{\top}\mathbf{D}^{\left( k \right)}\left( \boldsymbol{\beta}^{\boldsymbol{\star}} \right)$ converges in law to a centred normal distribution with variance $\phi^{\star}\mathbf{a}^{\top}\mathcal{T}_{\boldsymbol{\beta}^{\star}}^{\left( k \right)}\mathbf{a}$. An application of Cramér-Wold theorem concludes the proof of the Lemma. ◻

The Lemma below can be proven using similar arguments, so their proofs are omitted.

**Lemma 4**. *Under Conditions (C1) to (C5), it holds as* $n\to\infty$ *that*

| $\sup_{\boldsymbol{\beta}\in\Theta}\vert\sqrt{n^{\left( k \right)}}[\mathbf{V}^{\left( k \right)}\left( \boldsymbol{\beta} \right)-E\{\mathbf{V}^{\left( k \right)}\left( \boldsymbol{\beta} \right)\}]=O_{\mathbb{P}}\left( 1 \right)$ | $\left( 27 \right)$ |
| --- | --- |

For all $k\in\{1,\ldots,K\}$.

The next lemma establishes the consistency of ${\hat{\boldsymbol{\beta}}}_{MLE}^{\left( k \right)}$.

**Lemma 5**. *Under Conditions (C1) to (C5), it holds as* $n\to\infty$ *that* ${\hat{\boldsymbol{\beta}}}_{\text{MLE}}^{\left( k \right)}=\boldsymbol{\beta}^{\star}+o_{\mathbb{P}}\left( 1 \right)$ *for all* $1\leq k\leq K$*.*

To prove the Lemma, the goal is the apply Theorem 5.9 in [60] with $\theta\equiv\boldsymbol{\beta}$, $\Psi_{n}\equiv\mathbf{D}^{\left( k \right)}$ and $\Psi\equiv E\mathbf{D}^{\left( k \right)}$. To do this, it is required to verify that (a) it holds as $n\to\infty$ that

| $\sup_{\boldsymbol{\beta}\in\Theta}\vert\mathbf{D}^{\left( k \right)}\left( \boldsymbol{\beta} \right)-E\{\mathbf{D}^{\left( k \right)}\left( \boldsymbol{\beta} \right)\}\vert=o_{\mathbb{P}}\left( 1 \right) \text{for all }k\in\{1,\ldots,K\},$ |  |
| --- | --- |

and (b) that for every $\epsilon>0$, $\inf_{\boldsymbol{\beta}:\parallel\boldsymbol{\beta}-\boldsymbol{\beta}^{\star}\parallel>\epsilon}\parallel E\{\mathbf{D}^{\left( k \right)}\left( \boldsymbol{\beta} \right)\}\parallel>0=\parallel E\{\mathbf{D}^{\left( k \right)}\left( \boldsymbol{\beta}^{\star} \right)\}\parallel$.

That (a) holds follows from the fact that under the Lemma’s condition, Lemma 2 applies. That (b) holds follows from the fact that under Condition (C2) the mapping $\boldsymbol{\beta}\to E\{\mathbf{D}^{\left( k \right)}\left( \boldsymbol{\beta} \right)\}$ is continuous, and that under Condition (C4) one has $E\{\mathbf{D}^{\left( k \right)}\left( \boldsymbol{\beta} \right)\}=0$ if and only if $\boldsymbol{\beta}=\boldsymbol{\beta}^{\star}$. Hence, Theorem 5.9 applies, thereby ensuring that ${\hat{\boldsymbol{\beta}}}_{\text{MLE}}^{\left( k \right)}=\beta^{\star}+o_{\mathbb{P}}\left( 1 \right)$. The fact that it holds for all $1\leq k\leq K$ follows from the fact that under Condition (C1) $K$ is finite.

The last three Lemmas ensure the following result.

**Lemma 6**. *Under Conditions (C1) to (C5), it holds as* $n\to\infty$ *that, for all* $1\leq k\leq K$*,* $\sqrt{n^{\left( k \right)}}\left( {\hat{\boldsymbol{\beta}}}_{\text{MLE}}^{\left( k \right)}-\boldsymbol{\beta}^{\star} \right)=\sqrt{n^{\left( k \right)}}\left( \mathcal{T}_{\boldsymbol{\beta}^{\star}}^{\left( k \right)} \right)^{-1}\mathbf{D}^{\left( k \right)}\left( \boldsymbol{\beta}^{\star} \right)+o_{\mathbb{P}}\left( 1 \right) .$ *Consequently,* $\sqrt{n^{\left( k \right)}}\left( {\hat{\boldsymbol{\beta}}}_{\text{MLE}}^{\left( k \right)}-\boldsymbol{\beta}^{\star} \right)$ *converges in distribution to a centred normal random variable with variance-covariance matrix given by* $\phi^{\star}\left( \mathcal{T}_{\boldsymbol{\beta}^{\star}}^{\left( k \right)} \right)^{-1}$*.*

See Theorem 5.21 in [60].

**Lemma 7**. *Under Conditions (C1) to (C5), it holds as* $n\to\infty$ *that, for all* $1\leq k\leq K$*, and any* $\hat{\boldsymbol{\beta}}$ *such that* $\hat{\boldsymbol{\beta}}=\boldsymbol{\beta}^{\star}+o_{\mathbb{P}}\left( 1 \right)$*,* $\mathbf{V}^{\left( k \right)}\left( \hat{\boldsymbol{\beta}} \right)=\mathcal{T}_{\boldsymbol{\beta}^{\star}}^{\left( k \right)}+o_{\mathbb{P}}\left( 1 \right) .$

Let $\Psi\left( \boldsymbol{\beta} \right)=E\{\mathbf{V}^{\left( k \right)}\left( \boldsymbol{\beta} \right)\}$. Lemma 4 implies that

| $\mathbf{V}^{\left( k \right)}\left( \hat{\boldsymbol{\beta}} \right)=\Psi\left( \hat{\boldsymbol{\beta}} \right)+o_{\mathbb{P}}\left( 1 \right) .$ |  |
| --- | --- |

Since it is assumed that $\hat{\boldsymbol{\beta}}=\boldsymbol{\beta}^{\star}+o_{\mathbb{P}}\left( 1 \right)$, and as $\Psi\left( \boldsymbol{\beta}^{\star} \right)=\mathcal{T}_{\boldsymbol{\beta}^{\star}}^{\left( k \right)}$, the result follows from the continuous mapping theorem.
